# Insight into selectivity of photocatalytic methane oxidation to formaldehyde on tungsten trioxide

**DOI:** 10.1038/s41467-024-49138-8

**Published:** 2024-06-01

**Authors:** Yingying Fan, Yuheng Jiang, Haiting Lin, Jianan Li, Yuanjiang Xie, Anyi Chen, Siyang Li, Dongxue Han, Li Niu, Zhiyong Tang

**Affiliations:** 1 Center for Advanced Analytical Science, Guangzhou Key Laboratory of Sensing Materials and Devices, Guangdong Engineering Technology Research Center for Photoelectric Sensing Materials and Devices, c/o School of Chemistry and Chemical Engineering, Guangzhou University, Guangzhou, 510006 P. R. China; 2 Chinese Academy of Science (CAS) Key Laboratory of Nanosystem and Hierarchy Fabrication, CAS Center for Excellence in Nanoscience, National Center for Nanoscience and Technology, 100190 Beijing, PR China; 3 Center for Nanochemistry, Peking University, 100871 Beijing, PR China; 4 Guangdong Provincial Key Laboratory of Psychoactive Substances Monitoring and Safety, Anti-Drug Technology Center of Guangdong Province, Guangzhou, 510230 PR China; 5 School of Chemical Engineering and Technology, Sun Yat-sen University, Zhuhai, 519082 P. R. China

**Keywords:** Photocatalysis, Photocatalysis, Catalytic mechanisms

## Abstract

Tungsten trioxide (WO_3_) has been recognized as the most promising photocatalyst for highly selective oxidation of methane (CH_4_) to formaldehyde (HCHO), but the origin of catalytic activity and the reaction manner remain controversial. Here, we take {001} and {110} facets dominated WO_3_ as the model photocatalysts. Distinctly, {001} facet can readily achieve 100% selectivity of HCHO via the active site mechanism whereas {110} facet hardly guarantees a high selectivity of HCHO along with many intermediate products via the radical way. In situ diffuse reflectance infrared Fourier transform spectroscopy, electron paramagnetic resonance and theoretical calculations confirm that the competitive chemical adsorption between CH_4_ and H_2_O and the different CH_4_ activation routes on WO_3_ surface are responsible for diverse CH_4_ oxidation pathways. The microscopic mechanism elucidation provides the guidance for designing high performance photocatalysts for selective CH_4_ oxidation.

## Abstract (web summary)

Tungsten trioxide (WO_3_) is considered the most promising photocatalyst for the highly selective oxidation of methane to formaldehyde, though the origins of its catalytic activity and reaction mechanism are still debated. Here, the authors use WO_3_ with {001} and {110} facets as model photocatalysts, demonstrating that the photocatalytic oxidation of methane to formaldehyde in WO_3_ depends on the surface structure, leading to different formaldehyde selectivity.

## Introduction

Photocatalytic CH_4_ oxidation using semiconductor and solar light enables green synthesis of one-carbon (C1) oxygenates like methanol (CH_3_OH) and HCHO to supply the key feedstock for chemicals production^1,2^. Compared with CH_3_OH, the photocatalytic CH_4_ oxidation to HCHO is more scientifically challenging. This is because, in contrast to one step conversion of CH_4_ to CH_3_OH, the preparation of HCHO from CH_4_ oxidation generally needs to undergo multiple intermediates conversion^1,3,4^_,_ and moreover HCHO is easily overoxidized to carbon dioxide (CO_2_)^3^.

**Fig. 1:**
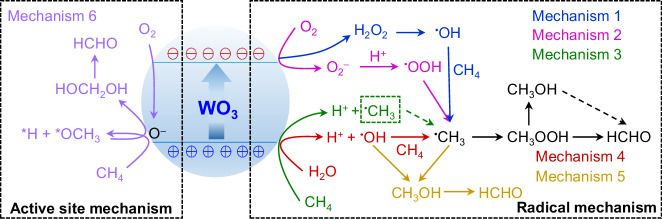
Multiple reaction pathways of photocatalytic CH_4_ oxidation to HCHO. Reported oxidation of CH_4_ to HCHO on WO_3_ photocatalyst following six types of reaction mechanisms.

To date, numerous semiconductor photocatalysts for CH_4_ oxidation to HCHO have been examined, such as titanium dioxide^5,6^, zinc oxide^1,7^ and WO_3_^8–10^. Amongst, WO_3_ is the only catalyst reported to be capable of generating HCHO with unity selectivity^9,10^. Unfortunately, due to the complex reaction process, diverse mechanisms on CH_4_ oxidation to HCHO in WO_3_ system have been proposed (Fig. 1)^8,9,11–14^, which in turn provide the confused guidance for the photocatalyst design. Generally, we classify the reaction mechanisms in Fig. 1 into two pathways. Mechanisms 1–5 represent the radical processes of HCHO formation through CH_4_ → CH_3_OOH → CH_3_OH → HCHO^12,15^. Owing to the excessive existence of intermediates (CH_3_OOH, CH_3_OH), the radical reaction processes are not conducive to the highly selective production of HCHO. Alternatively, mechanism 6 involves the active site, where CH_4_ is oxidized by lattice-O of WO_3_ to directly make HCHO^9^. Apparently, the selectivity of HCHO in mechanism 6 approaches 100%. However, the crucial factors that drive photocatalysts, not limited to WO_3_, following the desirable reaction mechanism remain largely unexplored.

In this work, we aim at understanding the origin of activity and selectivity of WO_3_ photocatalysts upon CH_4_ oxidation to HCHO. To simplify the investigation, all the reactions are performed under the identical reaction condition using WO_3_ with the same crystal structure as photocatalysts. Such prerequisite guarantees that the distinct reaction performance only correlates with the surface coordination environment of WO_3_. Therefore, we intentionally select the WO_3_ samples with same crystal structure but enclosed by different facets as the candidates to inspect the surface effect.

## Results and discussion

### Synthesis and characterization of catalysts

**Fig. 2:**
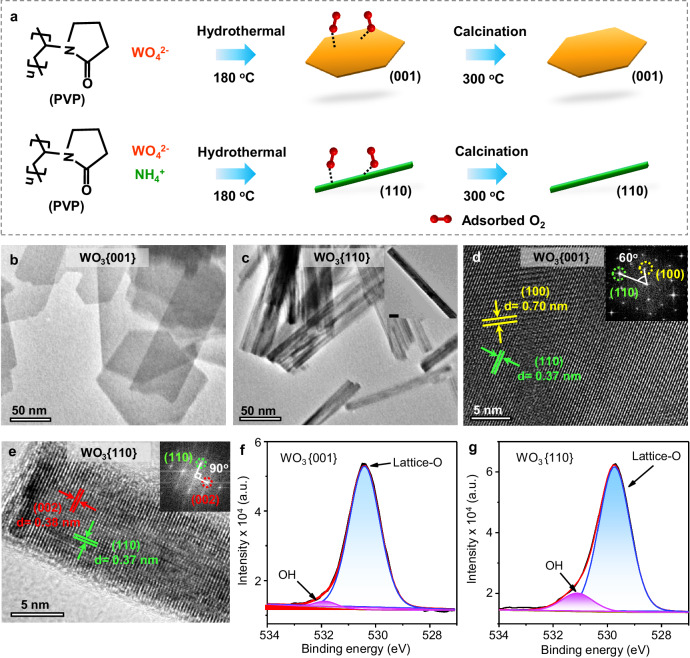
Synthesis and characterization of the photocatalysts. **a** Scheme of preparation process of WO_3_{001} and WO_3_{110}. **b** TEM images of WO_3_{001} and (**c**) WO_3_{110}. The insert is single WO_3_{110} nanowire with the scale bar as 10 nm. **d** HRTEM images of WO_3_{001} and (**e**) WO_3_{110} with the inset FFT images. **f** High-resolution O1*s* XPS spectra of WO_3_{001} and (**g**) WO_3_{110} after 300 ^o^C calcination.

The WO_3_ photocatalysts enclosed by {001} and {110}, named as WO_3_{001} and WO_3_{110}, were synthesized by 180 ^o^C hydrothermal treatment of Na_2_WO_4_•2H_2_O followed by calcination at 300 ^o^C (Fig. 2a). It is noted that the hydrothermal process with polyvinylpyrrolidone (PVP) as capping agent or ammonium ion (NH_4_^+^) as the directing agent facilitates the formation of {001} and {110} facets of WO_3_, respectively. Furthermore, characterization results of temperature-programmed desorption of O_2_ (O_2_-TPD), X-ray photoelectron spectroscopy (XPS) and Fourier-transform infrared spectroscopy (FTIR) confirm that the calcination at 300 ^o^C results in full removal of the chemical adsorbed O_2_ and the capping PVP or NH_4_^+^ from the catalyst surface (Supplementary Figs. 1–3). As shown in Fig. 2b, c, as-prepared WO_3_{001} and WO_3_{110} are of nanosheet and nanowire morphology, respectively. Their dominant surfaces are scrutinized by high-resolution transmission electron microscope (HRTEM) imaging with fast Fourier transformations (FFT). Figure 2d presents the clear lattice fringes of (110) (d = 0.37 nm) and (100) (d = 0.70 nm), suggesting that the electron beam transmits perpendicular to the exposed {001} crystal surface of WO_3_ nanosheet (Supplementary Fig. 4). Likewise, perpendicularly to {110} surface (Fig. 2e), lattice fringes of (110) (d = 0.37 nm) and (002) (d = 0.38 nm) are discerned in parallel with the long and short sides of WO_3_ nanowire. To further get the information on the surface composition, the high-resolution O1*s* XPS spectra of WO_3_{001} (Fig. 2f) and WO_3_{110} (Fig. 2g) are recorded, in which only lattice-O (ca. 530.4 eV)^16–18^ and OH species (ca. 531.8 eV)^19,20^ are distinguished without adsorbed O_2_. The OH species results from the surface hydroxide^17,18,21^, and the absence of chemisorbed O_2_ is consistent with the O_2_-TPD test (Supplementary Fig. 2b)^2,22^. Finally, the X-ray diffraction (XRD) patterns indicate that both WO_3_{001} and WO_3_{110} possess the hexagonal crystal structure (Supplementary Fig. 5).

### Photocatalytic CH_4_ oxidation performance

**Fig. 3:**
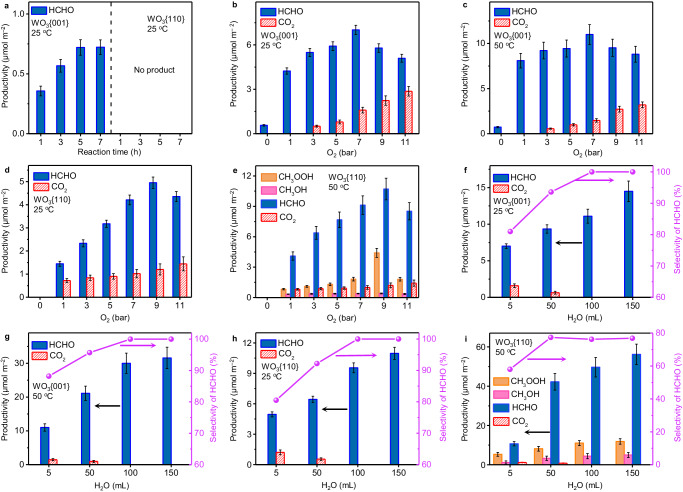
Photocatalytic oxidation of CH_4_ under different conditions. **a** Photocatalytic CH_4_ oxidation performance on WO_3_{001} and WO_3_{110} in pure CH_4_ atmospheres with reaction time prolonging at 25 ^o^C. **b** Photocatalytic CH_4_ oxidation performance on WO_3_{001} at 25 ^o^C and (**c**) 50 ^o^C with variation of O_2_ amounts. **d** Photocatalytic CH_4_ oxidation performance on WO_3_{110} at 25 ^o^C and (**e**) 50 ^o^C with variation of O_2_ amount. **f** Photocatalytic CH_4_ oxidation performance on WO_3_{001} at 25 ^o^C and (**g**) 50 ^o^C with variation of H_2_O amount. **h** Photocatalytic CH_4_ oxidation performance on WO_3_{110} at 25 ^o^C and (**i**) 50 ^o^C with variation of H_2_O amount. Reaction condition: (**a**–**e**) 10 mg catalyst, 3 h reaction time, Xenon light 150 mW cm^–2^, 5 mL H_2_O, pressures of CH_4_ + O_2_ = 20 bar; (**f**–**i**) 10 mg catalyst, 3 h reaction time, Xenon light 150 mW cm^–2^, 7 bar O_2_ + 13 bar CH_4_ (WO_3_{001}), 9 bar O_2_ + 11 bar CH_4_ (WO_3_{110}). Error bars indicate standard deviations.

Following the previous reports^1,3,4^, we carried out the photocatalytic CH_4_ oxidation with aqueous solution in a high-pressure reactor, where the total pressure of CH_4_ and O_2_ was maintained at 20 bar and the volume of H_2_O was fixed at 5 mL. Reaction temperature was controlled at 25 ^o^C or 50 ^o^C using water circulation to investigate the temperature effect. Before the photocatalytic experiments, the nitrogen adsorption–desorption isotherm curves were utilized to calculate the multipoint Brunauer–Emmett–Teller specific surface area of WO_3_{001} (Supplementary Fig. 6a–c) and WO_3_{110} (Supplementary Fig. 6d–f), being 15.70 m^2^ g^–1^ and 16.63 m^2^ g^–1^, respectively. We notice that the ratio of the specific surface area (0.94) of WO_3_{001}/WO_3_{110} is similar to that of the geometric surface area (~1, Supplementary Figs. 7–9). Thus, we take the specific surface area of WO_3_{001}and WO_3_{110} for catalytic activity comparison. The WO_3_{001} and WO_3_{110} samples were first subjected to pure CH_4_ atmosphere to reveal their intrinsic oxidation property. Only HCHO without other liquid products is produced on WO_3_{001} at both 25 ^o^C (Fig. 3a, Supplementary Fig. 10) and 50 ^o^C (Supplementary Fig. 11), indicating that CH_4_ is directly oxidized to HCHO not through other intermediates. The absence of CO_2_ is possibly ascribed to the low concentration of HCHO, which is not enough to be overoxidized (Supplementary Fig. 12). With the reaction time prolonging, the productivity of HCHO does not increase after 5 h (0.72 μmol m^–2^). Since H_2_O and WO_3_{001} are sole oxygen sources in pure CH_4_ atmosphere, the O-atom of HCHO must originate from one of them. If the abundant H_2_O is the oxygen source, the HCHO formation from CH_4_ oxidation will not stop at 5 h. Thus, we speculate that the finite surface lattice-O from WO_3_{001} provides the O-atom of HCHO and limits it production in CH_4_ atmosphere as previously reported^9,10^. It is worth mentioning that during the CH_4_ oxidation on WO_3_{001}, no H_2_ product is detected (Supplementary Fig. 13). As for WO_3_{110} system, no product is found in CH_4_ atmosphere at both 25 ^o^C (Fig. 3a, Supplementary Figs. 14, 15) and 50 ^o^C (Supplementary Fig. 11). Thus, we deduce that the lattice-O of WO_3_{110} cannot oxidize CH_4_ to HCHO.

The effect of O_2_ on CH_4_ oxidation over WO_3_{001} and WO_3_{110} was revealed in the mixed CH_4_ + O_2_ atmosphere. Compared to the reaction of WO_3_{001} in pure CH_4_ atmosphere, the participation of 7 bar O_2_ brings ca. ninefold (7.05 μmol m^–2^ at 25 ^o^C, Fig. 3b and Supplementary Fig. 16) and 15-fold (11.04 μmol m^–2^ at 50 ^o^C, Fig. 3c and Supplementary Fig. 17) enhancement in the yield of HCHO. Benefitting from the rise of reaction temperature, the total yield is also increased by 1.45 times at 7 bar O_2_ from 8.68 μmol m^–2^ (25 ^o^C) to 12.57 μmol m^–2^ (50 ^o^C). The increased HCHO production is likely caused by the sustainable renewal of lattice-O with the added O_2_ for CH_4_ oxidation^9,10^, and the elevation of reaction temperature promotes this process. The descended HCHO production after 7 bar O_2_ may be attributed to the reduction in partial pressure of CH_4_ and the overoxidation to CO_2_ (Supplementary Figs. 18–20). As comparison, with the addition of O_2_, HCHO is also produced in WO_3_{110} system with the highest productivity of 4.96 μmol m^–2^ (Fig. 3d, Supplementary Figs. 21, 22) at 25 ^o^C. To check if any other intermediates are formed, the reaction temperature is elevated to 50 ^o^C and many types of liquid products including CH_3_OOH, CH_3_OH and HCHO are discerned (Fig. 3e, Supplementary Figs. 23–25). At 9 bar of O_2_, the yield of HCHO reaches a maximum value of 10.71 μmol m^–2^ (50 ^o^C). The maximum total yield on WO_3_{110} is improved by 2.73 times by reaction temperature increasing from 25 ^o^C to 50 ^o^C, which is considerably higher than WO_3_{001}. Evidently, the HCHO formation on WO_3_{110} experiences the process of CH_4_ → CH_3_OOH → CH_3_OH → HCHO or CH_4_ → CH_3_OOH → HCHO. The descended liquid products on WO_3_{110} upon O_2_ pressure of larger than 9 bar are also assigned to the reduction in partial pressure of CH_4_ and the overoxidation to CO_2_. The distinct effect of reaction temperature on CH_4_ oxidation performance between WO_3_{001} and WO_3_{110} is attributed to their different kinetic properties of Arrhenius and non-Arrhenius behaviors, respectively, which are discussed detailedly in the reaction kinetics analysis part in supplementary information. The higher activation energy of CH_4_ oxidation on WO_3_{110} than WO_3_{001} leads to lower catalytic performance at 25 ^o^C. However, the non-Arrhenius behavior on WO_3_{110} makes its reaction rate highly depend on the reaction temperature, thus the maximum productivity over WO_3_{110} surpasses WO_3_{001} at 50 ^o^C. Besides, the promoted reaction rate of WO_3_{110} at 50 ^o^C also accelerates the formation of intermediates, contributing to the appearance of CH_3_OOH and CH_3_OH signals.

The solvent volume has considerable influence on the photocatalytic selectivity and activity. With H_2_O volume increasing from 5 to 150 mL at the fixed pressure of 7 bar O_2_ and 13 bar CH_4_ in WO_3_{001} system, the productivity of HCHO increases to 14.49 μmol m^–2^ at 25 ^o^C (Fig. 3f, Supplementary Fig. 26) or 31.59 μmol m^–2^ at 50 ^o^C (Fig. 3g, Supplementary Fig. 27), possibly resulting from the improved dissolution of CH_4_ in H_2_O solvent^7^. Besides, the CO_2_ signal diminishes gradually and eventually disappears in 150 mL H_2_O (Supplementary Fig. 28, 25 ^o^C and Supplementary Fig. 29, 50 ^o^C), leading to the 100% selectivity of HCHO product. The disappearance of CO_2_ signal is attributed to the reduced concentration of HCHO as previously reported (Supplementary Figs. 26, 27)^3,7^. While for WO_3_{110}, all the yields of CH_3_OOH, CH_3_OH and HCHO grows with H_2_O volume increasing. Despite the selectivity of HCHO approaching 100% at 25 ^o^C in 150 mL H_2_O (Fig. 3h, Supplementary Figs. 30, 31), it is only 73.73% when the reaction temperature rises to 50 ^o^C (Fig. 3i, Supplementary Figs. 32–35). This result correlates with the non-Arrhenius dependence over WO_3_{110} involved with radical mechanism. Besides, the HCHO selectivity enhancement with H_2_O volume increasing from 5 to 150 mL for both WO_3_ {001} and WO_3_{110} in our work does not involve the change of reaction mechanism, which is described in supplementary information (Supplementary Figs. 36, 37). Both cyclic test and long-term reaction for CH_4_ oxidation on WO_3_{001} and WO_3_{110} reveal their excellent photocatalytic stability (Supplementary Figs. 38–45). Additional verification experiments were also accomplished. The measured quantum efficiency values of both WO_3_{001} and WO_3_{110} follow the their diffuse reflectance spectra (Supplementary Figs. 46–48 and Supplementary Table 1), implying that the oxidation of CH_4_ to HCHO on WO_3_{001} and WO_3_{110} involves photocatalytic reaction process. The contrast experiments in the absence of light, catalyst or CH_4_ do no acquire the products (Supplementary Table 2).

### Mechanism investigation

**Fig. 4:**
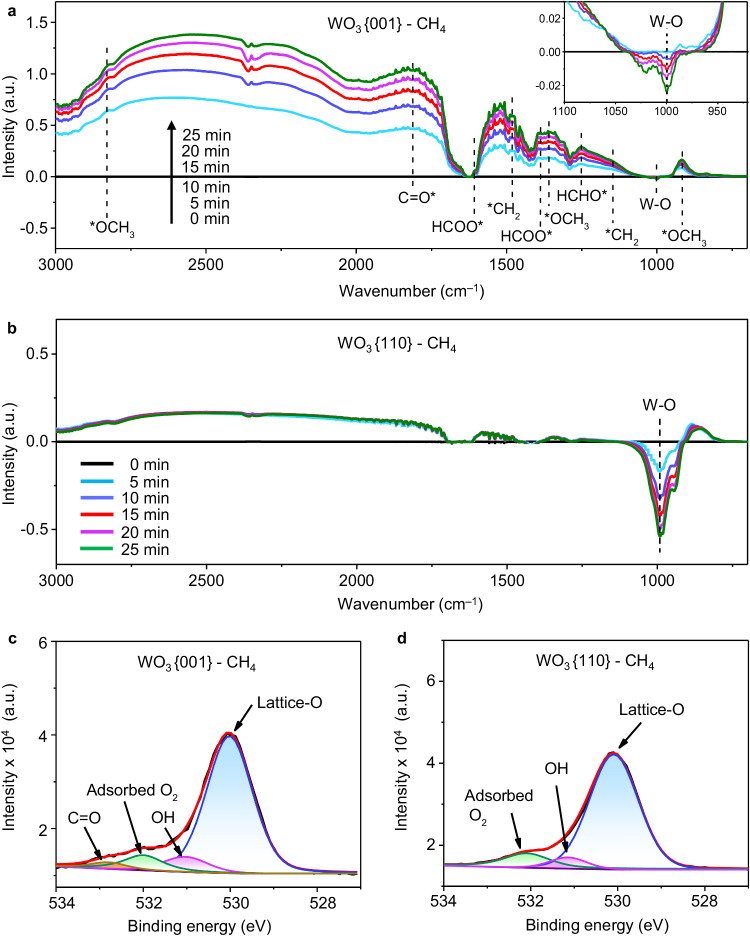
Investigation on the role of lattice-O in CH_4_ oxidation. In situ DRIFTS spectra of (**a**) WO_3_{001} and (**b**) WO_3_{110} in CH_4_ atmosphere under different light irradiation time without H_2_O addition. Here, * denotes an adsorption site on surface. The inset is the magnified W-O peak. **c** High-resolution O1*s* XPS spectra of WO_3_{001} and (**d**) WO_3_{110} after reaction in CH_4_ atmosphere.

In situ diffuse reflectance infrared Fourier transform spectroscopies (DRIFTS) performed in pure CH_4_ atmosphere with or without H_2_O addition are used to investigate the active site mechanism on WO_3_{001} for CH_4_ oxidation. Figure 4a, b shows that in pure CH_4_ atmosphere without H_2_O addition, no peak is observed prior to light irradiation (0 min, black curves) on both WO_3_{001} and WO_3_{110}. Once the light turns on, a series of peaks emerge on WO_3_ {001} (Fig. 4a). The peaks at 917, 1363 and 2830 cm^–1^ are attributed to the stretching vibration of C-H bond in the adsorbed *OCH_3_ species^2^, while the ones at 1149 and 1478 cm^–1^ are assigned to the vibration of adsorbed *CH_2_ species^16,17^. Both *OCH_3_ and *CH_2_ are believed as the crucial intermediates upon the direct CH_4_ oxidation to HCHO^9^. The peaks at 1251 and 1810 cm^–1^ are ascribed to the adsorbed HCHO* and C = O* species, further verifying the HCHO formation^18,19^. Note that the adsorbed HCOO* at 1381 and 1594 cm^–1^ might be the intermediate for overoxidation to CO_2_^2^. Noteworthily, the consumption of lattice-O is evidenced by continuous descending of the W-O peak at 989 cm^–1^ below zero baseline^20,21^. Clearly, CH_4_ is steadily oxidized to HCHO by lattice-O of WO_3_{001}. As comparison, no rise of carboxyl peaks on WO_3_{110} is found along with irradiation time whereas a mass of lattice-O is lost (Fig. 4b). This result discloses that HCHO cannot be generated through CH_4_ oxidation by lattice-O of WO_3_{110}, which is consistent with the catalytic experiments (Fig. 3a). Besides, no new peak is observed in both WO_3_{001}and WO_3_{110} systems after addition of H_2_O (Supplementary Fig. 49) with CH_4_ atmosphere, indicating that H_2_O molecule does not involve in CH_4_ oxidation. The causes of lattice-O consumption on WO_3_{001} and WO_3_{110} as well as their quantitative comparison are explained in detail in the theoretical calculation section and the ^•^OH radical analysis section see below. Moreover, the O1*s* XPS spectra of WO_3_{001} (Fig. 4c) and WO_3_{110} (Fig. 4d) after reaction in CH_4_ atmosphere were recorded to quantitatively measure the change of lattice-O. The intensity of lattice-O peaks is reduced by 25% (WO_3_{001}) and 35% (WO_3_{110}) after reaction in CH_4_ atmosphere (Supplementary Table 3), which is in accordance with the DRIFTS results. And the C = O peak (533.46 eV) is merely detected on WO_3_{001} but not on WO_3_{110}, presenting the HCHO production. Finally, the appearance of adsorbed O_2_ peak is attributed to surface oxygen vacancy, which is formed by the removal of surface lattice-O.

**Fig. 5:**
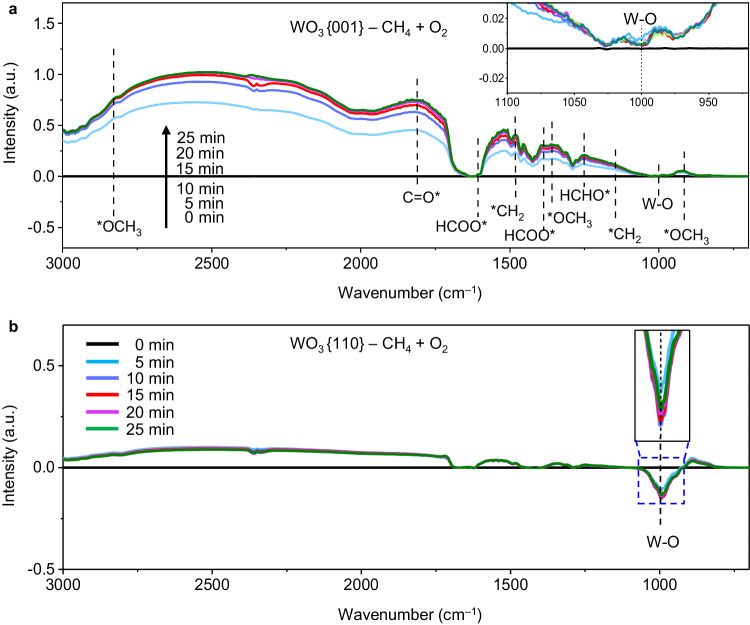
Investigation on the role of O_2_ in CH_4_ oxidation. In situ DRIFTS spectra of (**a**) WO_3_{001} and (**b**) WO_3_{110} in the mixed CH_4_ + O_2_ atmosphere under different light irradiation time without H_2_O addition. Here, * denotes an adsorption site on surface. The insets highlight the magnified W-O peak.

To explore the role of O_2_ in CH_4_ oxidation on WO_3_{001} and WO_3_{110}, in situ DRIFTS experiments were also conducted in the mixed CH_4_ + O_2_ atmosphere without (Fig. 5a, b) or with H_2_O addition (Supplementary Fig. 50a and 50b). Similar signals on WO_3_{001} emerge as that in CH_4_ atmosphere, indicating that there is no new surface reaction pathway (Fig. 5a and Supplementary Fig. 50a). Exceptionally, the W-O peak raises above the zero baseline (insets), signifying that the consumed lattice-O of WO_3_{001} is replenished adequately in O_2_ atmosphere. The regeneration of lattice-O is also confirmed by the O1*s* XPS of WO_3_{001} (Supplementary Fig. 51a). Such timely supplement of lattice-O guarantees the sustaining oxidation of CH_4_ to HCHO. For WO_3_{110} sample, similar signals are also found between CH_4_ + O_2_ atmosphere (Fig. 5b) and pure CH_4_ atmosphere (Fig. 4b) in absence of H_2_O. The negative W-O signal indicates that the lost lattice-O in WO_3_{110} could not be totally repaired in O_2_ atmosphere, which is also validated by 13.9% decrease in O1*s* XPS peak intensity (Supplementary Fig. 51b and Supplementary Table 4). After H_2_O addition in CH_4_ + O_2_ atmosphere, signals of *OCH_3_, C = O*, *CH_2_ and HCHO* appear on WO_3_{110} (Supplementary Fig. 50b). This result indicates that the photocatalytic CH_4_ oxidation reaction over WO_3_{110} is implemented through a radical process, and the addition of H_2_O enables the radical reaction pathway happening.

**Fig. 6:**
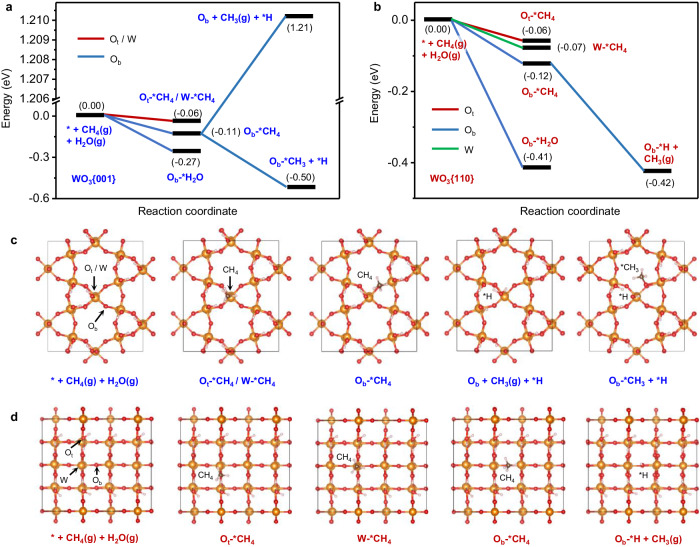
Elucidation of different CH_4_ oxidation pathways based on DFT calculation. Energy diagrams of CH_4_ and H_2_O adsorption as well as CH_4_ activation on the surface of (**a**) WO_3_{001} and (**b**) WO_3_{110} at the active sites of O_b_ (blue line), O_t_ (red line) and W (green line). Atomic configurations for the corresponding steps in the simulation of (**c**) WO_3_{001} and (**d**) WO_3_{110} (red – O, orange – W, gray – C, white – H).

To elucidate the different CH_4_ oxidation process on WO_3_{001} and WO_3_{110}, density functional theory (DFT) calculations were performed to examine their abilities responsible for CH_4_ and H_2_O adsorption as well as activation. Bridging O (O_b_), terminal O (O_t_) and W atoms are taken as the adsorption sites of CH_4_, respectively. It turns out that both WO_3_{001} (Fig. 6a, c) and WO_3_{110} (Fig. 6b, d) prefer CH_4_ adsorption on O_b_ sites (O_b_-*CH_4_) instead of O_t_ (O_t_-*CH_4_) and W sites (W-*CH_4_). To be specific for WO_3_{001}, through CH_4_ activation, a *CH_3_ group is formed and firmly adsorbed on its O_b_ site to generating *OCH_3_ group. Such species is confirmed by the rising *OCH_3_ signals in in situ DRIFTS spectra of WO_3_{001} in CH_4_ or CH_4_ + O_2_ atmospheres under light irradiation (Figs. 4a, 5a, Supplementary Figs. 49a, 50a). The positive energy of O_b_ + CH_3_(g) + *H (Δ*E* = 1.21 eV) means that the *CH_3_ group is hardly desorbed from WO_3_{001} surface. This deduction is proved by the EPR test of WO_3_{001} in CH_4_ or CH_4_ + O_2_ atmosphere, where no ^•^CH_3_ radical is observed (Supplementary Fig. 52a). Alternatively, the ^•^OH radical is derived from the adsorption of H_2_O molecules at O_b_ site (O_b_-*H_2_O, Fig. 6a and Supplementary Fig. 53a) with subsequent oxidation, which is not involved in CH_4_ oxidation as proved by in situ DRIFTS spectra before (Figs. 4a and 5a) and after H_2_O addition (Supplementary Figs. 49a and 50a). Thus, the CH_4_ oxidation process of WO_3_{001} for HCHO generation goes through an active site mechanism rather than a radical mechanism. To explore whether the H_2_O oxidation on WO_3_{001} surface affects the CH_4_ oxidation mechanism through O_b_ site consumption, the ^•^OH formation mechanism is investigated. According to previous reports, the formation of ^•^OH radical via H_2_O oxidation can be divided into two ways: one is that both O_b_ and photohole participate in H_2_O oxidation to generate one oxygen vacancy and two ^•^OH radicals (O_b_ + h^+^ + H_2_O → V_o_ + 2^•^OH)^23–25^; the other is simply hole oxidizing H_2_O to produce one H^+^ cation and one ^•^OH radical without O_b_ consumption (h^+^ + H_2_O → H^+^ + ^•^OH)^23,26^. As shown in Supplementary Fig. 54a and 55a, the positive energy of V_o_ + 2OH(g) (Δ*E* = 0.36 eV) on WO_3_{001} reveals that the ^•^OH radical formation is not through the process of O_b_ + h^+^ + H_2_O → V_o_ + 2^•^OH. While the negative energy of *H + OH(g) (Δ*E* = −0.91 eV, Supplementary Figs. 54a and 55b) confirms that the ^•^OH radical is generated through h^+^ + H_2_O → H^+^ + ^•^OH process without O_b_ consumption. Therefore, the H_2_O oxidation on WO_3_{001} surface does not change the CH_4_ oxidation process as no O_b_ is consumed.

As for WO_3_{110}, Fig. 6b uncovers that after CH_4_ activation on O_b_ site of (O_b_-*H + CH_3_(g)), the adsorbed *CH_3_ group is hardly formed and easily desorbed to form CH_3_(g) leaving O_b_-H group. Thus, the active site mechanism of CH_4_ oxidation by lattice-O does not occur on WO_3_{110}. Due to the higher surface energy of WO_3_{110} (4.13 J m^–2^)^22,27^, the O_b_-H is also easily desorbed to form oxygen vacancy, which is similar to the H_2_O oxidation to form oxygen vacancy and ^•^OH radical discussed later. This is consistent with in situ DRIFTS results in CH_4_ atmosphere without H_2_O addition, where no CH_4_ oxidation is discerned only with the detected loss of W-O signal (Fig. 4b). Actually, in an aqueous environment, due to the large difference of energy between the adsorbed H_2_O and CH_4_ molecules on the surface of WO_3_{110} (Fig. 6b and Supplementary Fig. 53b), O_b_ preferentially adsorbs H_2_O molecules (O_b_-*H_2_O) to block the adsorption of CH_4_ (O_b_-*CH_4_). And as displayed in Supplementary Figs. 54b and 55c, the adsorbed H_2_O molecule is easily oxidized to ^•^OH radical through O_b_ + h^+^ + H_2_O → V_o_ + 2^•^OH (Δ*E* = −1.24 eV, V_o_ + 2OH(g)). During this process, O_b_ is consumed along with oxygen vacancy generation, further excluding the possibility of CH_4_ oxidation at O_b_ active site. This is proved by the signal of W-O consumption in absence of CH_4_ activation in in situ DRIFTS spectra of WO_3_{110} in CH_4_ atmospheres with H_2_O addition (Supplementary Fig. 49b). Thereby, only ^•^OH radical without ^•^CH_3_ radical is observed in pure CH_4_ atmosphere for WO_3_{110} aqueous system (Supplementary Fig. 52b). Altogether, the CH_4_ oxidation process on WO_3_{110} for HCHO generation follows a radical mechanism instead of an active site mechanism. We note that the large difference in the energy for H_2_O molecules adsorption on surfaces of WO_3_{001} and WO_3_{110} stems from the number of hydrogen bonds formed. On WO_3_{001} surface, H_2_O molecule is adsorbed through one hydrogen bond, while on WO_3_{110} surface, two hydrogen bonds are formed after H_2_O adsorption. Therefore, WO_3_{110} has a higher adsorption capacity for H_2_O molecules than WO_3_{001}.

**Fig. 7:**
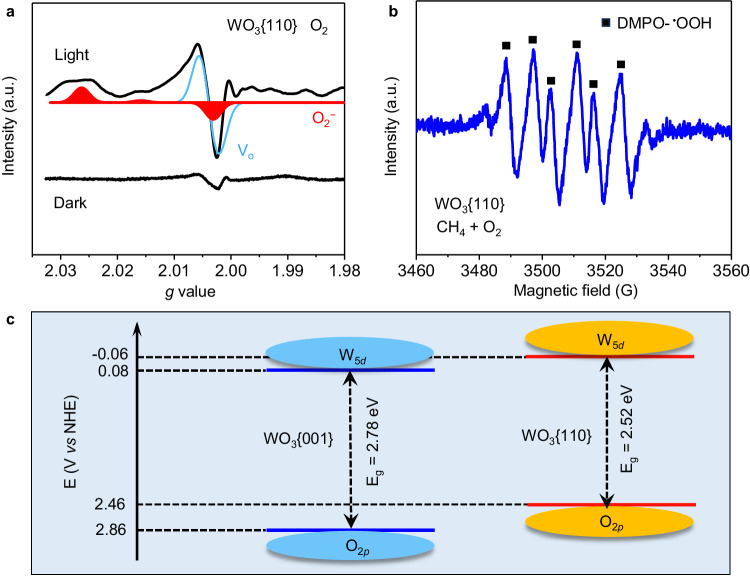
Determination of reactive radical species. **a** EPR spectra of WO_3_{110} in O_2_ atmosphere at 77 K temperature. The WO_3_{110} is the recycled sample after CH_4_ oxidation reaction without O_2_. **b** EPR spectrum of WO_3_{110} under light irradiation for 80 s with CH_4_ and O_2_ dissolved in methanol. DMPO is added to the reaction mixture as the radical trapping agent. The WO_3_{110} is the recycled sample after CH_4_ oxidation reaction without O_2_. **c** Band energy diagrams of WO_3_{001} and WO_3_{110}.

The reactive radical species in WO_3_{110} system is monitored by EPR spectroscopy. In pure O_2_ atmosphere, three signals at g = 2.027, 2.017 and 2.003 appear on WO_3_{110} surface upon photoirradiation (Fig. 7a). These three signals of orthorhombic symmetry are the characteristic hallmarks of surface-dwelling O_2_^−^ anions^2,5^, which are stabilized at the W sites (Eqs. (1)–(4)). The surface-dwelling O_2_^−^ anion is capable of breaking CH_4_ molecule to form ^•^CH_3_ radical and ^•^OOH radical (Eq. (5)) with the regeneration of oxygen vacancy (Eq. (6))^5,28^, which are proved by the 5,5-dimethyl-1-pyrroline N-oxide (DMPO) -^•^CH_3_ (Supplementary Fig. 52b) and DMPO-^•^OOH (Fig. 7b and Supplementary Fig. 56) signals in EPR spectra^29^, respectively. Finally, as-formed ^•^CH_3_ radical combines with ^•^OOH radical to produce CH_3_OOH (Eq. (7)) that is unstable and decomposed to CH_3_OH and HCHO (Eqs. (8), (9))^7^. Although ^•^OH radical was also detected, it was mainly been quenched and not involved in oxygenates production, which has been explained in supplementary information (Supplementary Figs. 57–68). As previous reports^5,30,31^, CH_3_OOH can be spontaneously decomposed into HCHO, whereas the conversion of CH_3_OOH into CH_3_OH is an electron reduction process. Thus, Eq. (9) becomes the major pathway of HCHO formation. This is the reason why HCHO is the main product in WO_3_{110} system. Altogether, the rich oxygen vacancies in WO_3_{110} facilitate the formation of O_2_^−^ anion, then promoting HCHO generation via the radical way. On the contrary, neither O_2_^−^ anion (Supplementary Fig. 69a) nor ^•^CH_3_ (Supplementary Fig. 69b) and ^•^OOH (Supplementary Fig. 69c) radicals are observed in the WO_3_{001} system, excluding involvement of the radical process in HCHO formation. The redox potential energy for the intermediate formation over WO_3_{110} is provided in supplementary information (Supplementary Note 3).

1 $${{{{{{\rm{W}}}}}}}^{6+}-{{{{{{\rm{O}}}}}}}^{2-}+{{{{{\rm{hv}}}}}}\to {{{{{{\rm{W}}}}}}}^{5+}-{{{{{{\rm{O}}}}}}}^{-}$$

2 $${{{{{{\rm{W}}}}}}}^{5+}-{{{{{{\rm{O}}}}}}}^{-}+{{{{{{\rm{H}}}}}}}_{2}{{{{{\rm{O}}}}}}\to {{{{{{\rm{W}}}}}}}^{5+}{{\ldots }}{{{{{{\rm{OH}}}}}}}^{-}+{\,\!}^{\bullet }{{{{{\rm{OH}}}}}}$$

3 $${{{{{{\rm{W}}}}}}}^{5+}\cdots {{{{{\rm{OH}}}}}}\to {{{{{{\rm{W}}}}}}}^{5+}-{{{{{{\rm{V}}}}}}}_{{{{{{\rm{o}}}}}}}+{\,\!}^{\bullet }{{{{{\rm{OH}}}}}}$$

4 $${{{{{{\rm{W}}}}}}}^{5+}-{{{{{{\rm{V}}}}}}}_{{{{{{\rm{o}}}}}}}+{{{{{{\rm{O}}}}}}}_{2}\to {{{{{{\rm{W}}}}}}}^{6+}-{{{{{{\rm{O}}}}}}}_{2}^{-}$$

5 $${{{{{{\rm{W}}}}}}}^{6+}-{{{{{{\rm{O}}}}}}}_{2}^{-}+{{{{{{\rm{CH}}}}}}}_{4}+{{{{{{\rm{h}}}}}}}^{+}\to {{{{{{\rm{W}}}}}}}^{6+}-{{{{{{\rm{V}}}}}}}_{{{{{{\rm{o}}}}}}}+{\,\!}^{\bullet }{{{{{\rm{OOH}}}}}}+{\,\!}^{{\bullet }}{{{{{{\rm{CH}}}}}}}_{3}$$

6 $${{{{{{\rm{W}}}}}}}^{6+}-{{{{{{\rm{V}}}}}}}_{{{{{{\rm{o}}}}}}}+{{{{{{\rm{e}}}}}}}^{-}\to {{{{{{\rm{W}}}}}}}^{5+}-{{{{{{\rm{V}}}}}}}_{{{{{{\rm{o}}}}}}}$$

7 $${\,\!}^{\bullet }{{{{{{\rm{CH}}}}}}}_{3}+{\,\!}^{\bullet }{{{{{\rm{OOH}}}}}}\to {{{{{{\rm{CH}}}}}}}_{3}{{{{{\rm{OOH}}}}}}$$

8 $${{{{{{\rm{CH}}}}}}}_{3}{{{{{\rm{OOH}}}}}}+{2{{{{{\rm{W}}}}}}}^{5+}{{\cdots }}{{{{{\rm{OH}}}}}}+{2{{{{{\rm{e}}}}}}}^{-}\to {2{{{{{\rm{W}}}}}}}^{5+}-{{{{{{\rm{O}}}}}}}^{-}+{{{{{{\rm{CH}}}}}}}_{3}{{{{{\rm{OH}}}}}}+{{{{{{\rm{H}}}}}}}_{2}{{{{{\rm{O}}}}}}$$

9 $${{{{{{\rm{CH}}}}}}}_{3}{{{{{\rm{OOH}}}}}}\to {{{{{\rm{HCHO}}}}}}+{{{{{{\rm{H}}}}}}}_{2}{{{{{\rm{O}}}}}}$$

The energy band potential is also responsible for the distinct CH_4_ oxidation mechanism between WO_3_{001} and WO_3_{110}. The energy band structure of both WO_3_{001} (Fig. 7c and Supplementary Fig. 70) and WO_3_{110} (Fig. 7c and Supplementary Fig. 71) is established with the valence band energy of 2.86 V and 2.46 V *vs* normal hydrogen electrode (NHE) and the conduction band energy of 0.08 V and −0.06 V *vs* NHE, respectively. The formation potential of O_2_^−^ anion from O_2_ reduction is reported to be −0.046 V *vs* NHE^23^, which is lower than −0.06 V of WO_3_{110} but higher than 0.08 V of WO_3_{001} (Fig. 7c). Therefore, WO_3_{110} rather than WO_3_{001} favors the formation of O_2_^−^ anion, leading to the generation of HCHO through the radical way. Alternatively, it is known that the top of valence band of WO_3_ is mainly composed of O2*p* orbitals^32,33^, and the obvious photocurrent under irradiation (Supplementary Fig. 72) on both WO_3_{001} and WO_3_{110} manifests that their lattice-O is activated through loosing electron. The more positive valence band of WO_3_{001} than WO_3_{110} could contribute to the preferential oxidation of CH_4_ to HCHO by lattice-O.

**Fig. 8:**
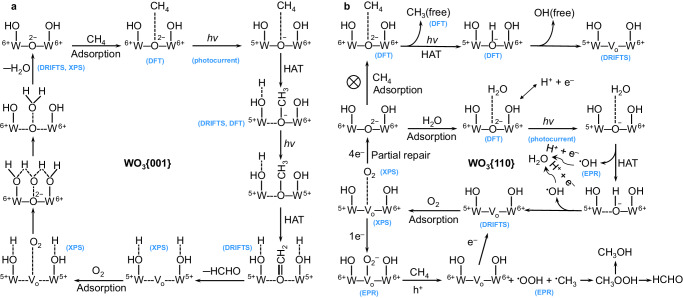
Proposed reaction mechanism. **a** The schematic illustration of the proposed mechanism for photocatalytic oxidation of CH_4_ on WO_3_{001} and (**b**) WO_3_{110}. V_o_ is the oxygen vacancy.

Armed with the above results, we attain the insight into the photocatalytic mechanisms of WO_3_{001} (Fig. 8a) and WO_3_{110} (Fig. 8b) toward CH_4_ oxidation. In both cases of WO_3_{001} and WO_3_{110}, the CH_4_ molecules are preferentially attached to the lattice-O_b_, which are disclosed by the DFT calculations (Fig. 6a, b). It is known that the valence band maximum of WO_3_ is mainly composed of O2*p* orbitals, meanwhile the conduction band minimum is mainly constituted by W5*d* orbitals^9,34^. Under light irradiation, the photoelectron from the valence band of WO_3_ is excited to the conduction band, that is, from O2*p* orbitals to W5*d* orbitals. This excitation makes the valence state of O_b_ change from O^2–^ to O^–^ and W atom from W^6+^ to W^5+^. Therein, the O^–^ is the photohole (h^+^) and the W^5+^ is photoelectron (e^–^)^34–36^. After the CH_4_ adsorption at the O_b_ site, the O^–^ (h^+^) of WO_3_{001} is able to insert into the C-H bond of CH_4_ molecule and sequentially forms the *OCH_3_ and *CH_2_ species, as shown in in situ DRIFTS spectra of WO_3_{001} in CH_4_ and CH_4_ + O_2_ atmospheres (Figs. 4a, 5a). Meanwhile the left H atom from CH_4_ is abstracted by adjacent -OH on W site via hydrogen atom transfer (HAT) process, as revealed by DFT results (O_b_-*CH_3_ + *H, Fig. 6a, c). Finally, the *OCH_2_ species is desorbed to form HCHO molecule. During this process, the O^–^ (h^+^) is consumed and e^–^ makes the W partially reduced (W^5+^), which is inspected by the high-resolution W4*f* XPS spectra (Supplementary Fig. 73). The consumed O_b_ atom becomes an oxygen vacancy that is conducive to the adsorption of O_2_ molecule, as observed by the XPS spectra after photocatalytic reaction in pure CH_4_ atmosphere (Fig. 4c). The adsorbed O_2_ molecule is then reduced by photoelectrons (e^–^) from W^5+^ to fix the depleted O_b_ atom, which is confirmed by the XPS spectra after CH_4_ oxidation in CH_4_ + O_2_ atmosphere (Supplementary Fig. 51a, Supplementary Table 4) and in situ DRIFTS spectra of WO_3_{001} in CH_4_ + O_2_ atmosphere (Fig. 5a and Supplementary Fig. 50a). As a result, the e^–^ is consumed. The above process involves the HCHO formation and the utilization route of photogenerated electron-hole pairs of WO_3_{001}.

As for WO_3_{110} (Fig. 8b), though the adsorbed CH_4_ molecule may be activated by lattice-O_b_, the adsorbed *CH_3_ group is hardly formed as revealed by the DFT calculation (Fig. 6b, O_b_-*H + CH_3_(g)). Moreover, the large adsorption energy of H_2_O (Fig. 6b, O_b_-*H_2_O) at the O_b_ site further inhibits the above CH_4_ activation process. Upon H_2_O oxidation at the O_b_ site, the oxygen vacancy is formed by releasing ^•^OH radical (Fig. 4b, Supplementary Figs. 52b and 54b). With O_2_ addition, the adsorbed O_2_ molecule at the site of oxygen vacancy can be reduced to repair the left lattice-O atom or generate O_2_^−^ anion by the photoelectron from the conduction band of WO_3_{110}. Compared with the four-electron oxygen reduction to repair lattice-O, the one-electron oxygen reduction in O_2_^−^ generation pathway has lower kinetic energy barrier. Therefore, for WO_3_{110}, the consumed lattice-O of WO_3_{110} is only partially repaired, and O_2_ is mainly involved in the formation of O_2_^−^ anion. The O_2_^−^ anion activates CH_4_ molecule to produce ^•^CH_3_ (Supplementary Fig. 52b) and ^•^OOH (Fig. 7b) radicals. Through combination between ^•^CH_3_ and ^•^OOH radicals, CH_3_OOH is generated followed by decomposition to CH_3_OH and HCHO. Also, the difference in the reaction mechanism between this work and the previous works^3,37,38^ is discussed in detail (Supplementary Note 2 and Supplementary Fig. 74).

**Fig. 9:**
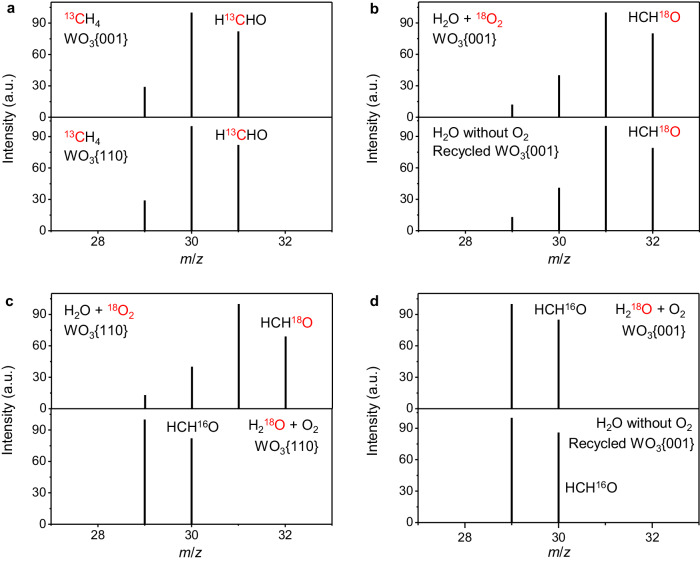
Trace of HCHO elements. **a** GC-MS spectra of HCHO obtained in WO_3_{001} and WO_3_{110} system using ^13^CH_4_ as carbon isotope. **b** GC-MS spectra of HCHO obtained in WO_3_{001} system using ^18^O_2_ as oxygen isotope and the recycled WO_3_{001} as photocatalyst without O_2_ addition. **c** GC-MS spectra of HCHO obtained in WO_3_{110} system using ^18^O_2_ or H_2_^18^O as oxygen isotope. **d** GC-MS spectra of HCHO obtained in WO_3_{001} system using H_2_^18^O as oxygen isotope and the recycled WO_3_{001} as photocatalyst without O_2_ addition.

To trace the carbon and oxygen sources of HCHO product, isotope tests were carried out. Employing ^13^CH_4_ as reactant (Supplementary Fig. 75), NMR experiments show that only the peak of HO^13^CH_2_OH at 81.9 ppm is detected on WO_3_{001} (CH_4_ atmosphere or the mixed CH_4_ and O_2_ atmosphere at 25 ^o^C) and WO_3_{110} (the mixed CH_4_ and O_2_ atmosphere at 25 ^o^C). Note that HOCH_2_OH is the diol structure of HCHO in aqueous solution, verifying that the C-atom in HCHO comes from CH_4_. The carbon source of HCHO in both WO_3_{001} and WO_3_{110} (Fig. 9a and Supplementary Fig. 76) systems was also inspected by gas chromatograph-mass spectrometer (GC-MS). The H^13^CHO peaks (*m/z* = 31) via ^13^CH_4_ oxidation proves that the C-atom of HCHO is from CH_4_. The origin of O-atoms in HCHO was traced by isotope labeling experiments with ^18^O_2_ and H_2_^18^O. In both WO_3_{001} (Fig. 9b, Supplementary Fig. 77) and WO_3_{110} (Fig. 9c, Supplementary Fig. 78) systems, HCH^18^O peaks (*m/z* = 32) are found taking ^18^O_2_ as reactant while H_2_^18^O makes no difference (Fig. 9d, Supplementary Fig. 79 and Fig. 9c, Supplementary Fig. 78), indicating that the O-atom of HCHO originates from O_2_. After the CH_4_ oxidation reaction in ^18^O_2_ atmosphere or taking H_2_^18^O as solvent, the WO_3_{001} photocatalyst was recycled and put into another CH_4_ oxidation system without O_2_ addition and with H_2_O as solvent. Taking the recycled WO_3_{001} from ^18^O_2_ atmosphere as photocatalyst, the clear signal of HCH^18^O confirms that the lattice-O atom of WO_3_{001} participates in the formation of HCHO and can be supplemented by O_2_. On the contrary, only HCHO without ^18^O labelling is found taking the recycled WO_3_{001} from H_2_^18^O solvent as photocatalyst, uncovering that the consumed lattice-O of WO_3_{001} cannot be repaired by H_2_O. Additionally, we note that no product is found in pure CH_4_ atmosphere using recycled WO_3_{110} as photocatalyst without O_2_ addition, which is reasonable considering that the consumed lattice-O of WO_3_{110} is hardly repaired. Based on the oxygen isotope experiments, we conclude that the O-atoms of HCHO products on WO_3_{001} and WO_3_{110} are both from O_2_ rather than H_2_O. Besides, for WO_3_{001} system, O_2_ is involved in the formation of HCHO by repairing the lattice-O. The different oxygen source analysis of CH_3_OH between this work and the previous work^38^ is provided in supplementary information (Supplementary Note 2). The missing peak signal (*m/z* = 28) of HCHO is analyzed in supplementary information (Supplementary Figs. 80–85).

In summary, we in-depth explore the HCHO formation mechanism from photocatalytic CH_4_ oxidation in WO_3_ system. The high oxidation potential, satisfied adsorption of activated CH_4_ molecule and low surface energy of WO_3_{001} confer the lattice-O to directly oxidize CH_4_ to HCHO without intermediates and ensure the 100% selectivity of HCHO through the active site oxidation mechanism. While, the WO_3_{110} with preferential activation of H_2_O and rich oxygen vacancy conforms to the free radical oxidation mechanism, possibly giving rise to a low selectivity of HCHO. This work not only provides pivotal insight into competitive catalytic pathways involved with the active site mechanism and the radical mechanism, but also opens the avenue towards optimizing the performance of important photocatalytic reactions, including but not limited to CH_4_ oxidation.

## Methods

### Photocatalyst preparation

Photocatalysts WO_3_{001} and WO_3_{110} were prepared through simple hydrothermal methods with subsequent calcination treatments.

For WO_3_{001}, Na_2_WO_4_.2H_2_O (2.7 g) and PVP (0.4 g) were dissolved in 50 mL water, then CH_3_COOH solution (8 mL) was added with continuous stirring for 30 min. After that, the suspension was transferred to a 100 mL Teflon-lined stainless-steel autoclave and treated under hydrothermal condition at 180 ^o^C for 12 h. The obtained powers were washed with deionized water until the pH = 7. After drying at 80 ^o^C overnight, the samples were calcinated at 300 ^o^C for 3 h with a heating rate of 3 ^o^C min^–1^. Finally, the desired WO_3_{001} was obtained.

For WO_3_{110}, Na_2_WO_4_.2H_2_O (2.7 g), PVP (0.4 g) and CH_3_COONH_4_ (0.4 g) were dissolved in 50 mL water, then CH_3_COOH solution (8 mL) was added with continuous stirring for 30 min. After that, the suspension was transferred to a 100 mL Teflon-lined stainless-steel autoclave and treated under hydrothermal condition at 180 ^o^C for 12 h. The obtained powers were washed with deionized water until the pH = 7. After drying at 80 ^o^C overnight, the samples were calcinated at 300 ^o^C for 3 h with a heating rate of 3 ^o^C min^–1^. Finally, the desired WO_3_{110} was obtained.

### Characterization

TEM and HRTEM were carried out using an FEI Tecnai G2 F20 electron microscope that was operated at 200 kV. The crystal structures were characterized through XRD patterns, which were obtained using a D/MAX-TTRIII (CBO) and Xeuss small-/wide-angle X-ray scattering (SAXS/WAXS) system with Cu Kα radiation (λ = 1.542 Å) operating at 50 kV and 300 mA. XPS experiments were carried out using an X-ray photoelectron spectrometer (EscaLab 250Xi, Thermo Scientific) and the spectra were calibrated with the C 1 s peak at 284.8 eV. UV-visible diffuse reflectance spectra (UV-Vis DRS) taking BaSO_4_ as the internal reference sample were recorded using a Hitachi U-3010 UV-visible spectrometer. A Mott-Schottky plot was obtained from samples in 1 M Na_2_SO_4_ solution at a frequency of 1500 Hz, prepared using a CHI 760E electrochemical workstation. The electron paramagnetic resonance (EPR) spectra were recorded at 9.43 GHz using a Bruker EMX spectrometer. In the case of the EPR test of O_2_^–^ anion: 25 mg photocatalysts were loaded into a quartz tube and gas of O_2_ or mixed O_2_ and CH_4_ was introduced for 20 min. Then, the EPR tests were carried out with or without Xenon light irradiation at a liquid nitrogen temperature (77 K). For the EPR test of oxygen vacancy: 25 mg photocatalysts were loaded into a quartz tube and tested at room temperature without light irradiation. For the EPR test of ^•^CH_3_ radical: 25 mg photocatalysts were loaded into a quartz tube with 5,5-dimethyl-1-pyrroline-1-oxide (DMPO) as the radical trapping agent in aqueous solution, then the test was carried out in the mixed O_2_ and CH_4_ atmosphere at room temperature with or without light irradiation, respectively. In situ DRIFTS tests were conducted by Thermo Scientific Nicolet IS52 in CH_4_ / CH_4_ + O_2_ atmosphere, with or without H_2_O addition, with or without light irradiation. Atomic force microscopy (AFM) was performed on Bruker Dimension Icon.

### Photocatalytic oxidation of CH_4_

The photocatalytic oxidation of CH_4_ was performed in a stainless-steel autoclave with a quartz glass window on the top. All the photocatalysis experiments were carried out at room temperature along with 25 ^o^C or 50 ^o^C cooling water and a fixed pressure of 20 bar. In a typical experiment, the photocatalyst sample (10 mg) was weighted and added in the center of the reactor with specified amounts of deionized water. 20 bar CH_4_ was inflated into reactor for the anaerobic reaction. Different ratio of CH_4_ and O_2_ with a total pressure of 20 bar was mixed and added in reactor for the aerobic reaction. A Xenon lamp (excitation wavelengths 300-700 nm, irradiation intensity of 150 mW cm^–2^, CEAULIOHT) or a light-emitting diode monochromatic light source (Perfectlight) was used to initiate the photocatalytic reactions. For ^18^O- and D-isotope tests, to prevent the exchange of -OH between HCHO and H_2_O in aqueous solution, H_2_O, H_2_^18^O and D_2_O was added in the form of steam, respectively, with HCHO as the gaseous product for GC-MS tests.

### Product analysis

Analysis of the oxygenated liquid product was carried out using NMR spectroscopy. The ^1^H NMR and ^13^C NMR spectra were recorded using a Bruker AVANCE III HD 400 MHz NMR spectrometer. The amount of HCHO was quantified using the acetylacetone colour-development method. Gaseous products were qualitatively and quantitatively determined by gas chromatography (GC) tests with flame ionization detector (FID) and thermal conductivity detector (TCD). Test condition of GC: inlet temperature 100 ^o^C, nitrogen as carrier gas with 0.1 MPa, column temperature of 60 ^o^C, FID temperature of 100 ^o^C, TCD bridge current of 60 mA.

The GC-mass spectrum (GC-MS) was performed on SHIMADZU with the SH-PolarWax column. Test condition: inlet temperature 180 ^o^C, splitless inlet, helium as carrier gas, linear speed of 25.5 cm s^–1^, column temperature of 40 ^o^C with 120 ^o^C pretreatment, GC-MS ion source temperature of 200 ^o^C.

### Reporting summary

Further information on research design is available in the Nature Portfolio Reporting Summary linked to this article.

## Supplementary information

Supplementary Information

Peer Review File

Reporting Summary

## Data Availability

All data supporting the findings of this study are available within the paper, supplementary information files or are available from the corresponding authors upon request.
