# Supplementary material for: Insight into selectivity of photocatalytic methane oxidation to formaldehyde on tungsten trioxide
Source: Nat Commun. 2024 Jun 1;15:4679. doi: 10.1038/s41467-024-49138-8 (PMC11535020; doi:10.1038/s41467-024-49138-8)
Supplement: Supplementary file 1 — Supplementary Information [file 41467_2024_49138_MOESM1_ESM.pdf]

Supplementary Information for Insight into selectivity of photocatalytic methane oxidation to formaldehyde on tungsten trioxide

Yingying Fan[1, 6], Yuheng Jiang[2, 3, 6], Haiting Lin[1, 6], Jianan Li[1], Yuanjiang Xie[1], Anyi Chen[1], Siyang Li[2], Dongxue Han[1, 4*], Li Niu[1, 5*], Zhiyong Tang[2*]

[1] Center for Advanced Analytical Science, Guangzhou Key Laboratory of Sensing Materials and Devices, Guangdong Engineering Technology Research Center for Photoelectric Sensing Materials and Devices, c/o School of Chemistry and Chemical Engineering, Guangzhou University, Guangzhou 510006, P. R. China.

[2] Chinese Academy of Science (CAS) Key Laboratory of Nanosystem and Hierarchy Fabrication, CAS Center for Excellence in Nanoscience, National Center for Nanoscience and Technology, Beijing 100190, PR China

[3] Center for Nanochemistry, Peking University, Beijing 100871, PR China

[4] Guangdong Provincial Key Laboratory of Psychoactive Substances Monitoring and Safety, Anti-Drug Technology Center of Guangdong Province, Guangzhou 510230, PR China

[5] School of Chemical Engineering and Technology, Sun Yat-sen University, Zhuhai 519082, P. R. China.

[6] The authors contributed equally: Yingying Fan, Yuheng Jiang, Haiting Lin

*e-mail: dxhan@gzhu.edu.cn; lniu@gzhu.edu.cn; zytang@nanoctr.cn

Supplementary Note 1

Supplementary Methods

Chemicals. Sodium tungstate dihydrate ($Na_2WO_4.2H_2O$), polyvinylpyrrolidone (PVP, Fw = 58000), acetic acid (CH_3COOH), ammonium acetate (CH_3COONH_4), deuterium oxide (D_2O), barium sulfate ($BaSO_4$), sodium sulfate (Na_2SO_4) and dimethyl sulfoxide (DMSO) were all obtained from Innochem. CH_4 and O_2 gases were purchased from KODI Corporation. Isotopic gases of $^{13}CH_4$ and $^{18}O_2$ were brought from LION Biology Corporation. Deionized water with a resistivity of 18.2 MΩ cm^{-1} was used in all experiments. All chemicals were used as received and without further purification.

The principle for photocatalyst preparation.

A simple hydrothermal method was used for WO_3\{001\} and WO_3\{110\} photocatalysts preparation (Fig. 2a). Adjusting the pH value of Na_2WO_4 solution by acetic acid facilitated the decomposition of tungsten source into WO_3 substance. As a capping agent, PVP could coordinate to W^{6+} ion of WO_3 (001) plane through its oxygen atom (O) of pyrrolidone group at high temperature[1-2], directing the anisotropic growth of WO_3 nanosheet perpendicular to the <001> crystal axis. Rinsing thoroughly with water, the WO_3\{001\} nanosheet was obtained. According to the previous reports[1, 3-4], the NH_4^+ cation was inclined to stabilize the surface of (110) probably by interaction with terminal O^{2-} ion, thus WO_3\{110\} with terminal crystal oxygen atoms was prepared. Finally, the calcination at 300 ºC was applied for both samples to fully remove the chemical adsorbed O_2 and the capping PVP or NH_4^+ from the catalyst surfaces.

Preparation of 0.25% (v/v) acetylacetone solution. Ammonium acetate (25 g) was dissolved in water (10 mL), and then acetylacetone (0.25 mL) and acetic acid (3 mL) were added in sequence. After that, deionized water was added to dilute the solution into 100 mL. With the pH adjusted to 6, the solution was finally prepared and stored at 2-5 ºC for up to one month.

Acetylacetone colour-development method. A portion of the product solution (1.5 mL) was mixed with as-prepared 0.25% (v/v) acetylacetone solution (1 mL) before being heated for 5 min in boiling water. Afterwards, the colour of the mixed solution turned

into yellow. Through absorbance detection at 413 nm, the HCHO concentration was obtained.

Computational details. The corresponding correction factors and the formula for "Energy" evaluation are provided as follows.

The spin-polarized density functional theory calculations were performed using Vienna ab initio Simulation Package (VASP) v.5.4.4[5-6]. Exchange-correlation interactions were described by the Perdew-Burke-Ernzerhof (PBE) functional[7] within the generalized gradient approximation (GGA). Projected augmented wave (PAW) method[8-9] was applied to describe the electron-ion interactions. Valence electrons were taken into account by using a plane-wave basis set with a kinetic energy cutoff of 400 eV. Gaussian smearing method with a width of 0.02 eV was chosen to determine the partial occupancies of Kohn-Sham orbitals. The electronic energy was considered self-consistent when the energy change was smaller than 10^{-5} eV. The force convergence tolerance was set to be 0.05 eV $Å^{-1}$ when conducting geometry optimization. The DFT-D3 method introduced by Grimme et al[10] was adopted to describe van de Waals interactions. The Brillouin zone sampling was achieved by using Monkhorst-Pack meshes of 2×2×1. A 15 Å vacuum space along the z direction was added to avoid the interaction between the two neighboring images.

The "Energy" (ΔE) was from the following equations based on previous reports[11-17]:

$$\Delta E = \Delta E_{\mathrm{DFT}} - neU_{\mathrm{g}} \quad \text{(S1)}$$

$$\Delta E_{\mathrm{DFT}} = E_{\mathrm{im/sub}} - E_0 = E_{\mathrm{im/sub}} - (E_{\mathrm{slab}} + E_{\mathrm{gas}}) \quad \text{(S2)}$$

where ΔE_{DFT} was the computed reaction energy obtained from VASP, $E_{\mathrm{im/sub}}$ was the computed energy of the optimized intermediate/substrate system, and $E_0 = E_{\mathrm{slab}} + E_{\mathrm{gas}}$ was the computed energy of the initial state that is set to 0. n was the number of photoelectrons left on the model by oxygen vacancy formation, e was the elementary charge, and U_{g} was the bandgap of the semiconductor. The latter term $-neU_{\mathrm{g}}$ in the formula is introduced to compensate the energy of V_o formation. This was because upon formation of a neutral oxygen vacancy, one photoelectron was supposed to undergo trapping[11-12]. The trapped photoelectron could not be described properly by GGA because of the self-interaction error in DFT[11, 18]. To solve this problem, the $-neU_{\mathrm{g}}$ correction should be applied[11-14].

Reaction kinetics analysis. The different effects of reaction temperature on CH_4 oxidation productivity and selectivity over WO_3\{001\} and WO_3\{110\} are attributed to their different kinetic properties, which could be further explained by their distinct reaction mechanisms. According to the previous reports, the CH_4 oxidation rate coefficient (k) of WO_3\{001\} with active site mechanism followed an Arrhenius behavior[19-23] (equation S3), while the k* of WO_3\{110\} with radical mechanism obeyed a non-Arrhenius dependence[24-28] (equation S4). For both Arrhenius and non-Arrhenius behaviors, the reaction rate constant increase with the reaction temperature:

$$k = A \exp(-E_a/RT) \quad \text{(S3)}$$

$$k^* = A^*T^n \exp(-E_a^*/RT) \text{ or } k^* = A^*T^6 \exp(-E_a^*/RT) \quad \text{(S4)}$$

where k and k^* represent the rate constants, A and A^* are the preexponential factors, T is the reaction temperature, E_a and E_a^* are the activation energy, R is the molar gas constant, and n is as high as 6.

According to the productivity of CH_4 oxidation within 3 h reaction time, the k values of WO_3\{001\} was calculated to be 2.89 μmol m^{-2} h^{-1} (25 °C) and 4.19 μmol m^{-2} h^{-1} (50 °C), then the E_a was calculated to be 11.89 kJ mol^{-1} based on equation S3. Similarly, the k^* values of WO_3\{110\} were 2.06 μmol m^{-2} h^{-1} (25 °C) and 5.63 μmol m^{-2} h^{-1} (50 °C), respectively, and the corresponding E_a^* was calculated to be 16.71 kJ mol^{-1}. The higher value of E_a^* than E_a gave rise to the lower CH_4 oxidation performance of WO_3\{110\} (6.19 μmol m^{-2} with 9 bar O_2, Fig. 3d) than WO_3\{001\} (8.68 μmol m^{-2} with 7 bar O_2, Fig. 3b) at 25 °C. However, non-Arrhenius behavior of k^* highly depended on T, thus the maximum productivity over WO_3\{110\} reached 16.88 μmol m^{-2} at 50 °C (Fig. 3e), obviously higher than WO_3\{001\} with 12.57 μmol m^{-2} (Fig. 3c). The promoted reaction rate k^* of WO_3\{110\} at 50 °C also accelerated the formation of intermediate products, resulting in the appearance of CH_3OOH and CH_3OH signals.

Supplementary Note 2

Comparison and analysis with previous works (1)

The different reaction mechanism between this work and the reported studies (*Luo, L. et al. Nat. Commun. 14, 2690 (2023)*, *Fan, Y. et al. J. Mater. Chem. A 8, 13277–13284 (2020)* and *Fan, Y. et al. Nat. Sustain. 4, 509–515 (2021)*)[29-31] is primarily associated with different reactive surface environment. In *Luo, L. et al. Nat. Commun. 14, 2690 (2023)*[29], single-atom Cu and $W^{\delta+}$ co-modified WO_3 ($Cu_{0.029}$-def-WO_3) is used as the photocatalyst for CH_4 oxidation. Therein, single-atom Cu exists in the form of Cu^{2+} bonding to two lattice-O adjacent to W, and serves as the electron acceptor. $W^{\delta+}$ is the reduced state of W^{6+} in WO_3, which is induced by the oxygen vacancy. Based on previous reports[32-34], O_2 is easily adsorbed at $W^{\delta+}$ and reduced to repair oxygen vacancy or generate reactive oxygen species (such as O_2^{-}). However, for the photocatalyst of $Cu_{0.029}$-def-WO_3 in this report of *Luo, L. et al. Nat. Commun. 14, 2690 (2023)*, since the adjacent Cu^{2+} acts as the electron acceptor, it abstracts the electrons from $W^{\delta+}$, causing $W^{\delta+}$ to become a hole acceptor $W^{(\delta+1)+}$. As-formed $W^{(\delta+1)+}$ then acts as the oxidation site for H_2O to generate $^{\bullet}OH$ and H^+ rather than the O_2 reduction site. Therefore, a large number of adsorbed $^{\bullet}OH$ is formed and becomes the main reactive species for CH_4 activation and oxidation to form HCHO. Meanwhile, the simulation result reveals that as-reduced Cu^+ can adsorb O_2 and the bond length of adsorbed O_2 is largely stretched to 1.41 Å in the molecular form. Due to the low oxidation states of the Cu^+, this stretched O_2 is further readily protonated by the adsorbed H_2O on $W^{(\delta+1)+}$ active site, which is reflected by the DMPO-$^{\bullet}$OOH signal with six prominent characteristic signals. Noteworthily, without single-atom Cu modification, O_2 is mainly reduced to DMPO-$^{\bullet}O_2^{-}$ with four-fold peaks on def-WO_3 and WO_3, which is consistent with our results. Therefore, no steady O_2^{-} over $Cu_{0.029}$-def-WO_3 can be formed to activate and oxidize CH_4. The above mechanism may be also applicable to Au modified ZnO sample and q-$BiVO_4$ nanoparticles in our own work[30-31], where Au acts as an electron acceptor to abstract the photoelectrons from Zn atom of ZnO, and V element of q-$BiVO_4$ can share the loading of photoelectrons from Bi element.

While in our manuscript, because no external electron acceptor is added, the W atom is the electron acceptor as the conduction band minimum of WO_3 is mainly constituted by W5*d* orbitals. For pristine WO_3\{110\}, H_2O molecules are easily adsorbed on lattice-O of WO_3\{110\} and oxidized to produce $^{\bullet}OH$ radicals with the

generation of oxygen vacancy and reduced W atom (W^{5+}). Therefore, during the photocatalytic process, WO_3{110} is also gradually enriched with oxygen vacancy defect, which is similar to def-WO_3 in *Luo, L. et al. Nat. Commun. 14, 2690 (2023)*. Subsequently, the oxygen vacancy site adsorbs O_2, which undergoes one-electron reduction to O_2^- or 4-electron reduction to repair lattice-O. Owing to the slow dynamic characteristics of 4-electron reduction, O_2 is more easily reduced to O_2^- by one electron with a minor reduction potential (-0.046 V *vs* NHE). Besides, similar to def-WO_3 in *Luo, L. et al. Nat. Commun. 14, 2690 (2023)*, the generated O_2^- will not be directly protonated by adjacent surface -OH groups and the evident signal of DMPO-$^{\bullet}O_2^-$ is found in absence of CH_4 addition (Supplementary Fig. 74). This guarantees its reactivity for CH_4 activation and oxidation, which is proved by the signal of DMPO-$^{\bullet}$OOH with CH_4 addition (Fig. 7b). Even if we assume that the $^{\bullet}$OH can activate CH_4 in our WO_3{110} system, it must be the adsorbed $^{\bullet}$OH according to the reports from *Luo, L. et al. Nat. Commun. 14, 2690 (2023)*, *Fan, Y. et al. J. Mater. Chem. A 8, 13277–13284 (2020)* and *Fan, Y. et al. Nat. Sustain. 4, 509–515 (2021)*). Based on the previous reports[35-37], the free $^{\bullet}$OH radicals are inclined to be adsorbed at the cation sites of metal oxide such as Ti_{5c} sites of TiO_2, $W^{\delta+}$ sites of W-doped $Ni(OH)_2$ and $Bi^{\delta+}$ sites of BiOCl, where the structure of adsorbed $^{\bullet}$OH radical is equal to the terminal -OH group. For our WO_3{110} surface, the terminal -OH already exists (Fig. 2g), so additional adsorption of $^{\bullet}$OH radical results in the presence of two -OH groups at a W site. Two adjacent -OH groups inevitably combine and shed one H_2O molecule, i.e. $^{\bullet}$OH radical is quenched by the surface -OH group (Supplementary Fig. 58a). In brief, unlike the previous works of *Luo, L. et al. Nat. Commun. 14, 2690 (2023)*, *Fan, Y. et al. J. Mater. Chem. A 8, 13277–13284 (2020)* and *Fan, Y. et al. Nat. Sustain. 4, 509–515 (2021)*, the different surface structure of WO_3{110} makes $^{\bullet}$OH radicals unable to activate and oxidize CH_4 molecule. Analogously, the terminal -OH groups also exist on the surface of WO_3{001}, thus the $^{\bullet}$OH radicals cannot activate and oxidize CH_4 in WO_3{001} systems.

Comparison and analysis with previous work (2)

The different oxygen source of CH_3OH between this work and the previous work (*Fan, Y. et al. J. Mater. Chem. A 8, 13277–13284 (2020)*) is mainly attributed to their different active sites for CH_4 oxidation. The detailed analysis is as follows. In the work of *Fan, Y. et al. J. Mater. Chem. A 8, 13277–13284 (2020)*, the photocatalyst is the Au

nanoparticles modified ZnO, such as $Au_{0.75}/ZnO$, where subscript 0.75 represents the mass ratio (0.75 wt%) between Au and ZnO nanoparticles. Known from the literatures[38-41], Au nanoparticles are considered as the active sites for CH_4 oxidation to form CH_3OH through the combination between $^{\bullet}CH_3$ and $^{\bullet}OH$ (or $^{\bullet}OOH$). In our work of *Fan, Y. et al. J. Mater. Chem. A 8, 13277–13284 (2020)*, $^{\bullet}OH$ radical is largely produced with 80% from H_2O oxidation and 20% from O_2 reduction. Meanwhile, no $^{\bullet}OOH$ signal is detected, indicating that O_2 is not reduced to O_2^-. This is because O_2 is mainly adsorbed in the side-on form[38] on the surface of Au nanoparticles, conducive to breaking O-O bond of O_2 forming $^{\bullet}OH$ radicals rather than O_2^-. Thus, on the surface of $Au_{0.75}/ZnO$, CH_3OH is generated by the combination of $^{\bullet}CH_3$ and $^{\bullet}OH$ radicals, and the $^{\bullet}OH$ radical is mainly from H_2O oxidation with a small part from O_2. Nevertheless, for $WO_3\{110\}$ in our present work, the generated amount of $^{\bullet}OH$ radical is trace and O_2 is reduced to O_2^-, which can oxidize CH_4 to CH_3OOH. The CH_3OH in $WO_3\{110\}$ system is generated by reduction of as-formed CH_3OOH. Altogether, the O-source of photocatalytic products by $WO_3\{110\}$ system is from O_2.

Supplementary Note 3

According to equation 1-9 for CH_4 oxidation on WO_3\{110\}, the CH_4 oxidation to HCHO and CH_3OH involves many intermediates including $^{\bullet}OH$ from H_2O oxidation ($^{\bullet}OH/H_2O$ = 2.38 V *vs* NHE)[38, 42], $^{\bullet}OOH$ from O_2 reduction ($O_2/^{\bullet}OOH$ = -0.046 V *vs* NHE[38, 42], and $^{\bullet}CH_3$ from CH_4 activation ($^{\bullet}CH_3/CH_4$ = 2.06 V *vs* NHE)[38, 43-44], which all locate within the band energy potential of WO_3\{110\}.

Supplementary Figures

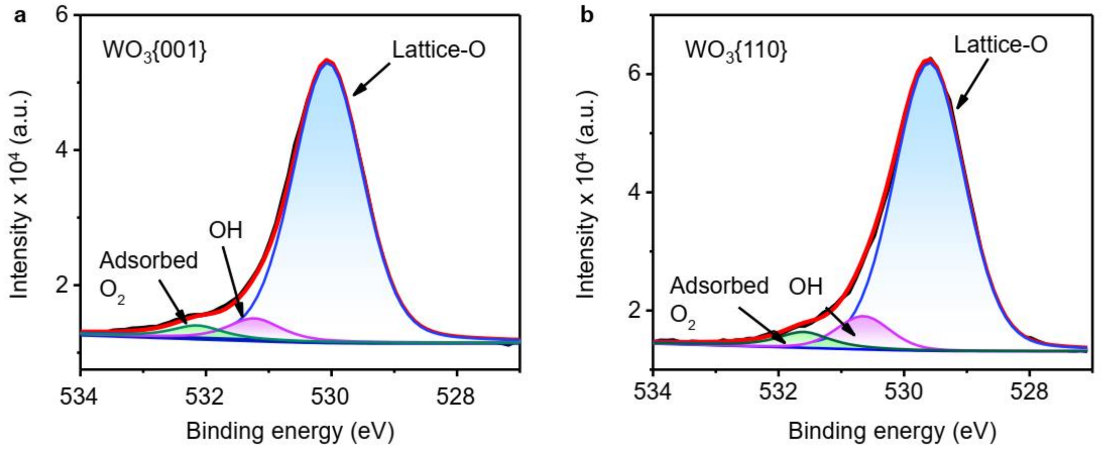

Supplementary Fig. 1 High-resolution O1*s* XPS spectra of (a) WO_3{001} and (b) WO_3{110} before calcination.

As shown in O1*s* XPS spectra, both WO_3{001} (Supplementary Fig. 1a) and WO_3{110} (Supplementary Fig. 1b) possess the characteristic peaks of surface adsorbed O_2 before calcination.

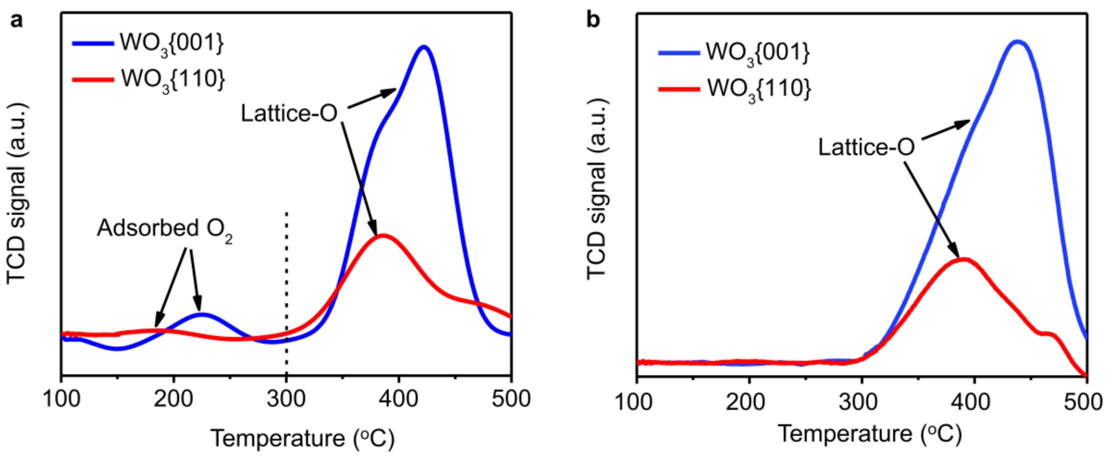

Supplementary Fig. 2 O_2-TPD spectra of WO_3\{001\} and WO_3\{110\} (a) before and (b) after calcination treatment at 300 ºC.

To obtain the surface free of O_2 adsorption, the temperature-programmed desorption of O_2 experiments (O_2-TPD) were conducted. As displayed in Supplementary Fig. 2a, the peaks ranging from 100 °C to 300 °C in the spectra are ascribed to the desorption of chemisorbed O_2 molecules[45-46], indicating that a temperature of 300 ºC can fully remove the adsorbed O_2 molecules from catalyst surface.

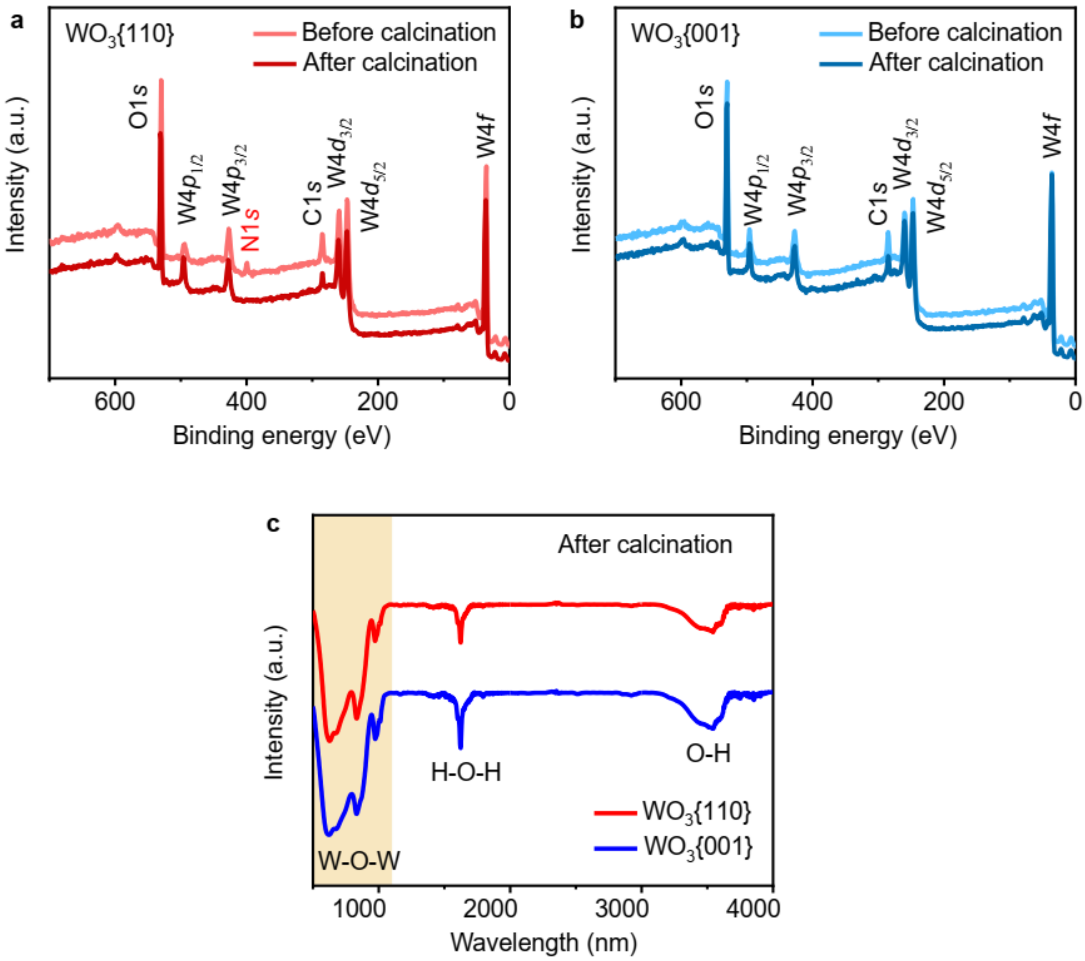

Supplementary Fig. 3 XPS spectra of (a) WO_3 {110} and (b) WO_3 {001} before and after calcination. (c) FTIR spectra of WO_3 {110} and WO_3 {001} after calcination.

The successful removal of capping and directing agents, that is PVP and NH_4^+, is proved by XPS and FTIR spectra. As shown in Supplementary Fig. 3a, compared to the samples before calcination, the N1*s* XPS peak disappears after calcination, indicating that the adsorbed NH_4^+ is removed on the surface of WO_3 {110}. No N1*s* peak is observed on WO_3 {001} before and after calcination (Supplementary Fig. 3b), because no NH_4^+ is added in the synthesis of WO_3 {001}. The C1*s* XPS peaks of both WO_3 {110} and WO_3 {001} are attributed to the contamination, which is unavoidable in XPS testing[47]. In order to investigate whether PVP remains, the samples were characterized by FTIR spectra on WO_3 {110} and WO_3 {001} after calcination. No organic group is observed in Supplementary Fig. 3c, manifesting that no PVP is left on the surface of both WO_3 {110} and WO_3 {001}.

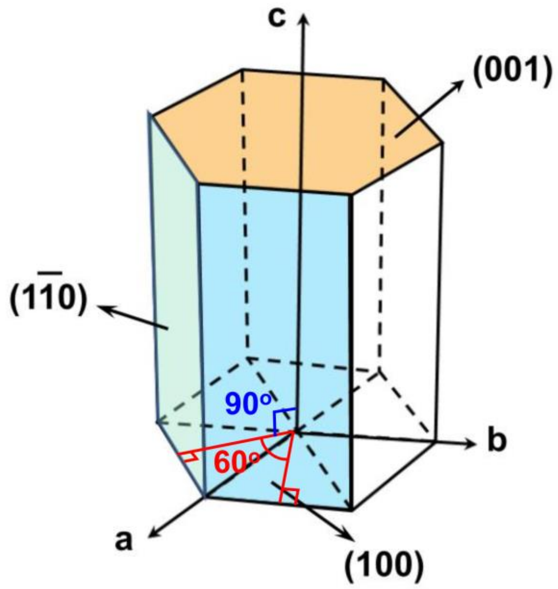

Supplementary Fig. 4 Geometrical configuration of hexagonal WO_3 crystal.

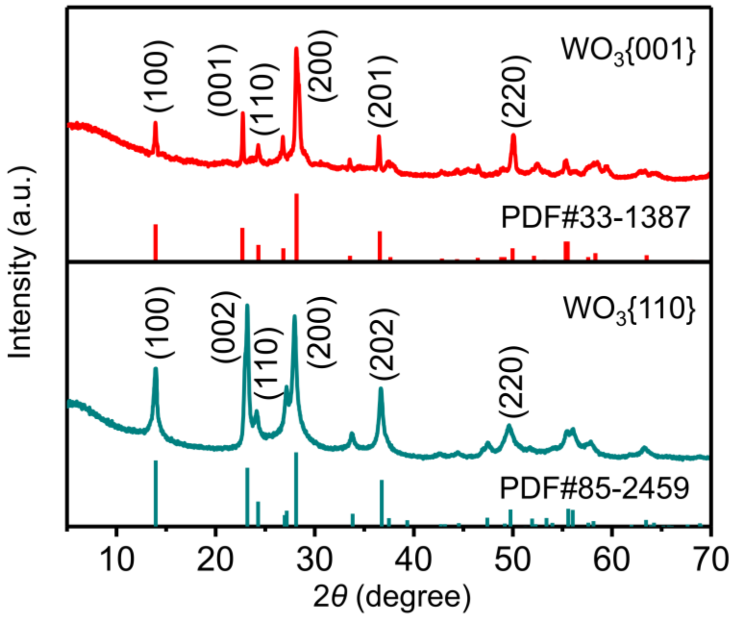

Supplementary Fig. 5 XRD patterns of WO_3 {001} and WO_3 {110}.

The successful preparation of WO_3 {001} and WO_3 {110} is verified by their characteristic XRD patterns[1, 45, 48], where the hexagonal XRD diffraction spectra of WO_3 {001} and WO_3 {110} belong to PDF#33-1387 and PDF#85-2459, respectively.

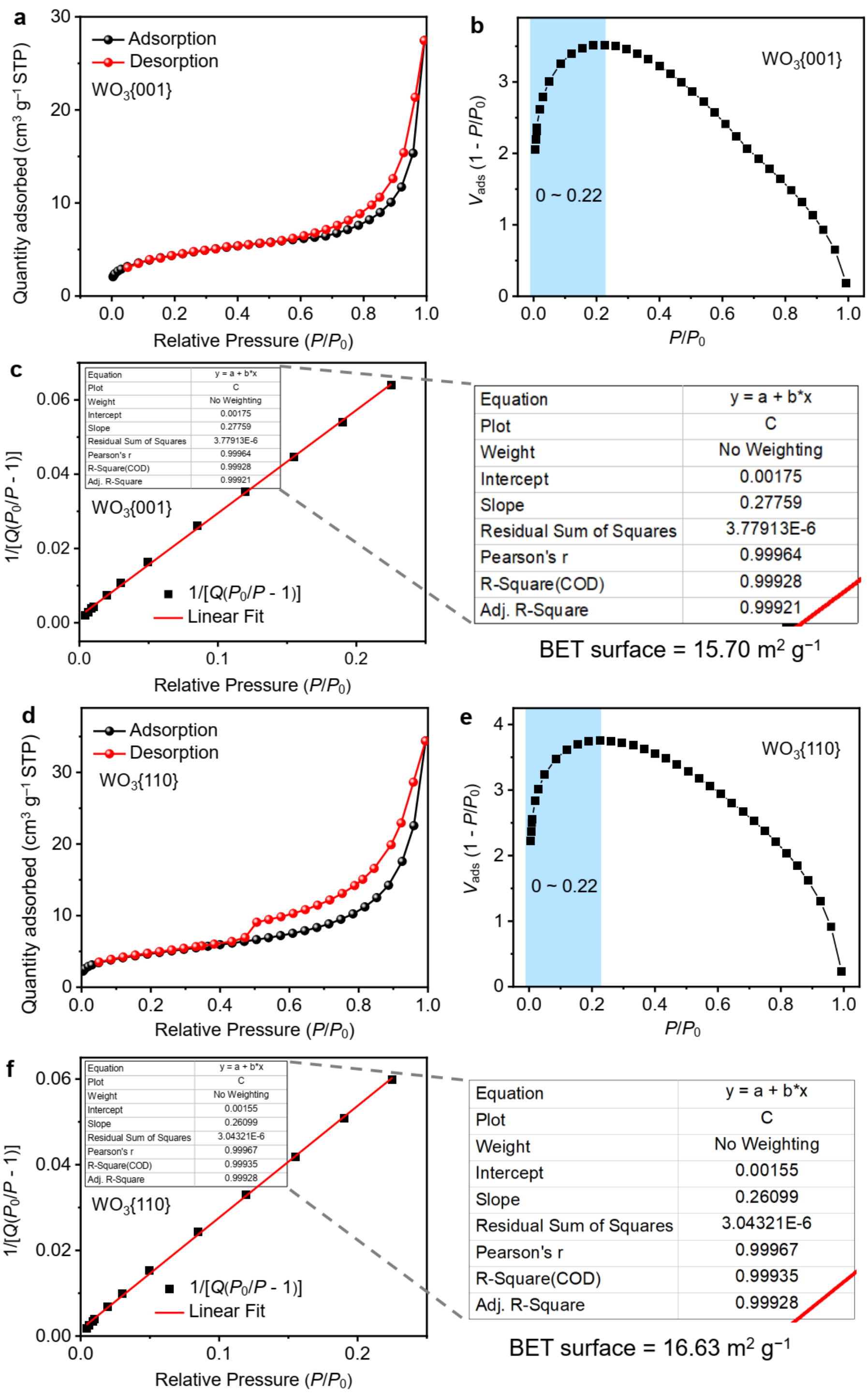

Supplementary Fig. 6 (a) N_2 adsorption–desorption isotherm curves of WO_3 {001}, and (b) the corresponding curve of $V_{ads}(1–P/P_0)$ *vs* P/P_0 to determine the low-pressure region. (c) Linear fit of $P/[V_{ads}(P_0-P)]$ (i.e. $1/[Q(P_0/P-1)]$, $Q = V_{ads}$) against P/P_0 under low-pressure region for WO_3 {001}. (d) N_2 adsorption–desorption isotherm curves of WO_3 {110}, and (e) the corresponding curve of $V_{ads}(1–P/P_0)$ *vs* P/P_0 to determine the low-pressure region. (f) Linear fit of $P/[V_{ads}(P_0-P)]$ (i.e. $1/[Q(P_0/P-1)]$, $Q = V_{ads}$) against P/P_0 under low-pressure region for WO_3 {110}.

The calculation process of BET surface area including the linear fitting is as follows. BET surface area is known to be derived from N_2 adsorption/desorption isotherm and calculated by the BET isothermal equation in general form as follows.

$$\frac{P}{V_{ads}(P_0 - P)} = \frac{C - 1}{V_m C}\left(\frac{P}{P_0}\right) + \frac{1}{V_m C} \tag{S5}$$

where V_{ads} is the volume of gas adsorbed at P; V_m is the monolayer volume of N_2 molecules on the surface of catalyst; P is the pressure of N_2 (at equilibrium); P_0 is the saturation pressure of N_2; C is the adsorption coefficient. Using P/P_0 as the horizontal coordinate and $P/[V_{ads}(P_0-P)]$ as the vertical coordinate, the V_m and C are obtained. Then, surface area can be calculated by V_m.

As shown in Supplementary Fig. 6a and 6d, during N_2 adsorption and desorption tests, despite a wide range of P/P_0 from 0.00 to 1.00 has been tested, only low-pressure region is used in the BET surface area calculation. This is because the low-pressure of P/P_0 ensures the monolayer adsorption of N_2, guaranteeing the accurate calculation of photocatalyst surface area. Whereas, the high-pressure of P/P_0 would cause the N_2 multilayer adsorption, which cannot be used in photocatalyst surface area calculation. To determine the low-pressure region, the curves of $V_{ads}(1-P/P_0)$ *vs* P/P_0 are drawn (Supplementary Fig. 6b and 6e). For both WO_3\{001\} and WO_3\{110\}, we can observe that the term $V_{ads}(1-P/P_0)$ continuously increases with P/P_0 in the range of 0 to 0.22. This means that in this low-pressure range, N_2 is adsorbed in form of monolayer (V_m) and the C is positive. Thus, in the range (P/P_0) of 0 to 0.22, the linear fit is performed between $P/[V_{ads}(P_0-P)]$ (i.e. $1/[Q(P_0/P-1)]$, $Q = V_{ads}$) and P/P_0 (Supplementary Fig. 6c and 6f). Through the intercept and slope values of the fitted lines, the V_m value is calculated to be 3.60 cm^3 g^{-1} STP (WO_3\{001\}) and 3.82 cm^3 g^{-1} STP (WO_3\{110\}) while C value is calculated to be 159.62 (WO_3\{001\}) and 169.38 (WO_3\{110\}). Therein, STP is the condition of standard temperature and pressure. Under the STP condition, each cm^3 of N_2 molecules paves into a monolayer and occupies an area of 4.354 m^2. Thus, the BET surface area of WO_3\{001\} = 3.60 cm^3 g^{-1} * 4.354 m^2 cm^{-3} = 15.70 m^2 g^{-1} and the BET surface area of WO_3\{110\} = 3.82 cm^3 g^{-1} * 4.354 m^2 cm^{-3} = 16.63 m^2 g^{-1}. This is the reason why WO_3\{001\} and WO_3\{110\} are equipped with different N_2 absorption and desorption curves, but possess the similar specific surface area.

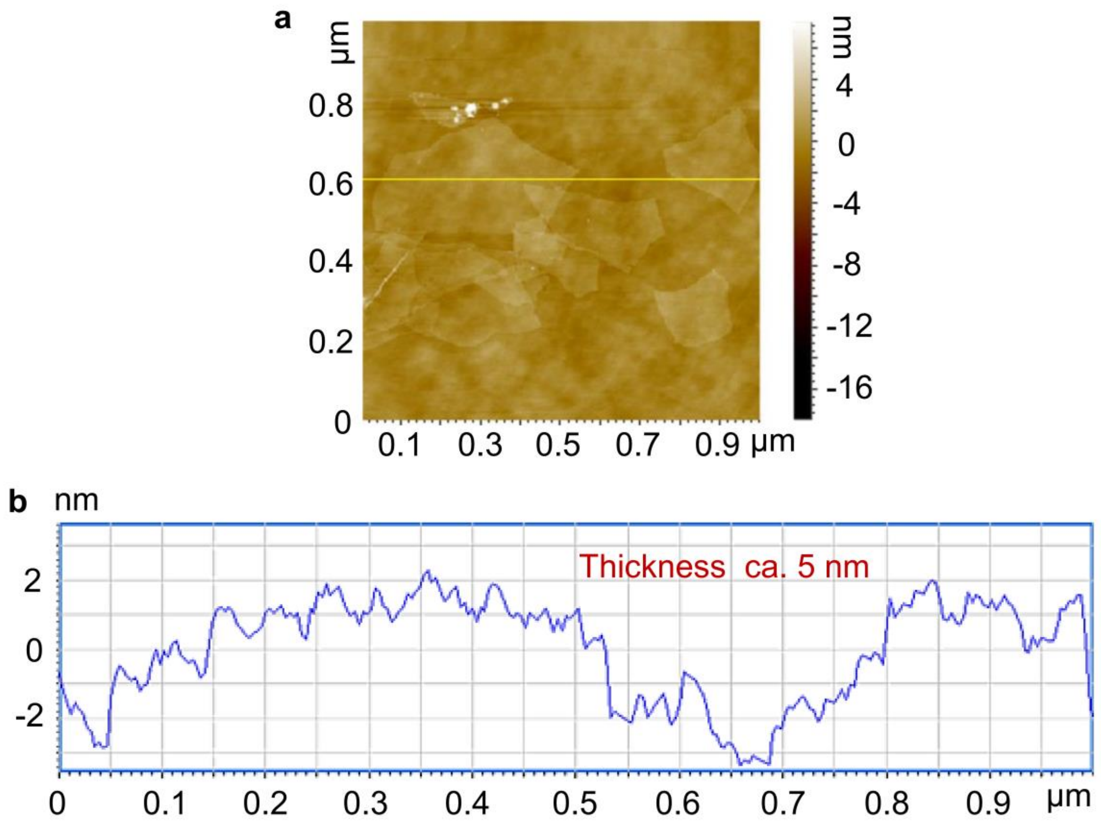

Supplementary Fig. 7 (a) AFM image of WO_3 {001} sheets dispersed on silicon and (b) AFM height profile of WO_3 {001} sheets.

The thickness of WO_3 {001} sheets is measured to be 5 nm by atomic force microscope (AFM) imaging.

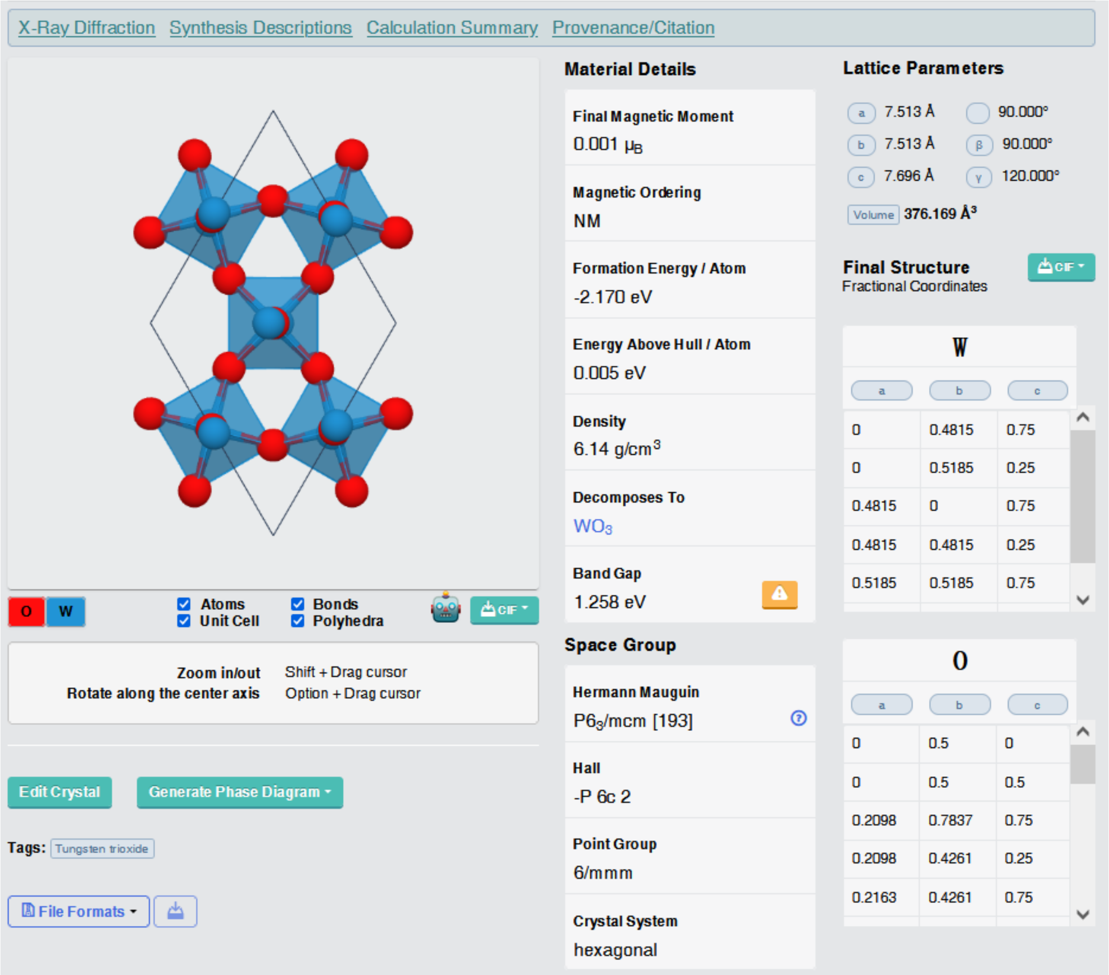

Supplementary Fig. 8 Hexagonal WO_3 crystals of the same mass density. (https://materialsproject.org/)

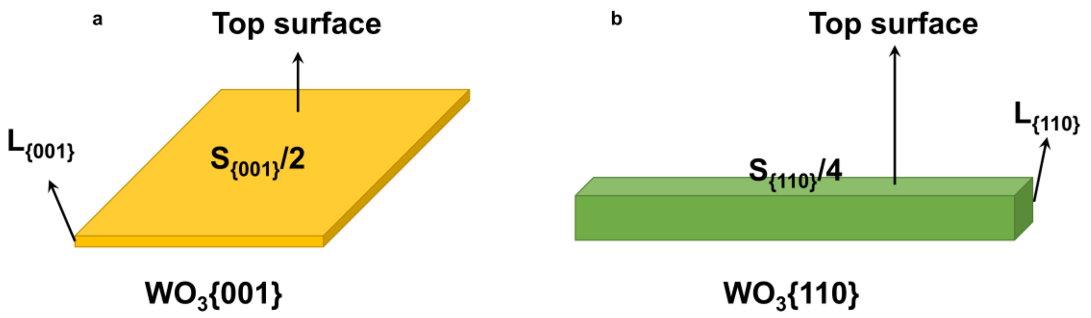

Supplementary Fig. 9 (a) Geometric model of $WO_3\{001\}$ and (b) $WO_3\{110\}$ in the shape of cubic sheet and rectangular cylinder, respectively.

Since both $WO_3\{001\}$ and $WO_3\{110\}$ belong to hexagonal crystal system, their mass density should be same (6.14 g cm^{-3}, Supplementary Fig. 8, https://materialsproject.org/). This feature is responsible for the same volume per gram of $WO_3\{001\}$ and $WO_3\{110\}$ catalysts, which is 0.163 cm^3 g^{-1}. To simplify the calculation, we assume that $WO_3\{001\}$ is a cubic sheet and $WO_3\{110\}$ is a rectangular cylinder (Supplementary Fig. 9). The surface area of $WO_3\{001\}$ and $WO_3\{110\}$ is labelled as $S_{\{001\}}$ and $S_{\{110\}}$, respectively. Since the {001} and {110} are the dominant facets of $WO_3\{001\}$ and $WO_3\{110\}$, their surface area (the top surface) can be approximately equal to $S_{\{001\}}/2$ and $S_{\{110\}}/4$, respectively. The thickness of $WO_3\{001\}$ and $WO_3\{110\}$ is labelled as $L_{\{001\}}$ and $L_{\{110\}}$, respectively. Then, we can get the equations:

$$V\{001\} = L_{\{001\}} * S\{001\} / 2 \quad (S6)$$

$$V\{110\} = L_{\{110\}} * S\{110\} / 4 \quad (S7)$$

$$V\{001\} = V\{110\} \quad (S8)$$

That is:

$$L_{\{001\}} * S\{001\} / 2 = L_{\{110\}} * S\{110\} / 4 \quad (S9)$$

$$S\{001\} / S\{110\} = L_{\{110\}} / 2L_{\{001\}} \quad (S10)$$

As shown in Supplementary Fig. 7b and 1c, we get that the value of $L_{\{001\}}$ and $L_{\{110\}}$ is 5 and 10 nm, respectively. Therefore, the ratio of $S\{001\}/ S\{110\}$ is 1. This value is very close to the BET result obtained by the N_2 adsorption–desorption isotherm curves.

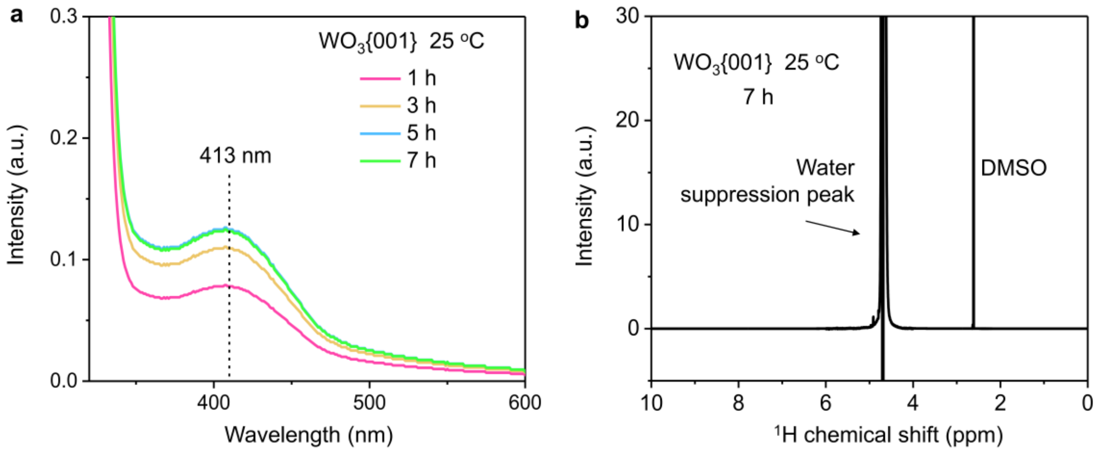

Supplementary Fig. 10 (a) UV-visible absorption spectra of HCHO product on WO_3 {001} in CH_4 atmosphere with different reaction time. (b) ^{1}H NMR spectrum of product on WO_3 {001} in CH_4 atmosphere for 7 h reaction time. Reaction condition: 10 mg catalyst, 20 bar CH_4, 5 mL H_2O volume, Xenon light 150 mW cm^{-2}, reaction temperature 25 °C.

Through acetylacetone color-developing reaction, the HCHO signals are discerned (Supplementary Fig. 10a). No other carbon oxygenates product is detected in ^{1}H NMR spectrum (Supplementary Fig. 10b).

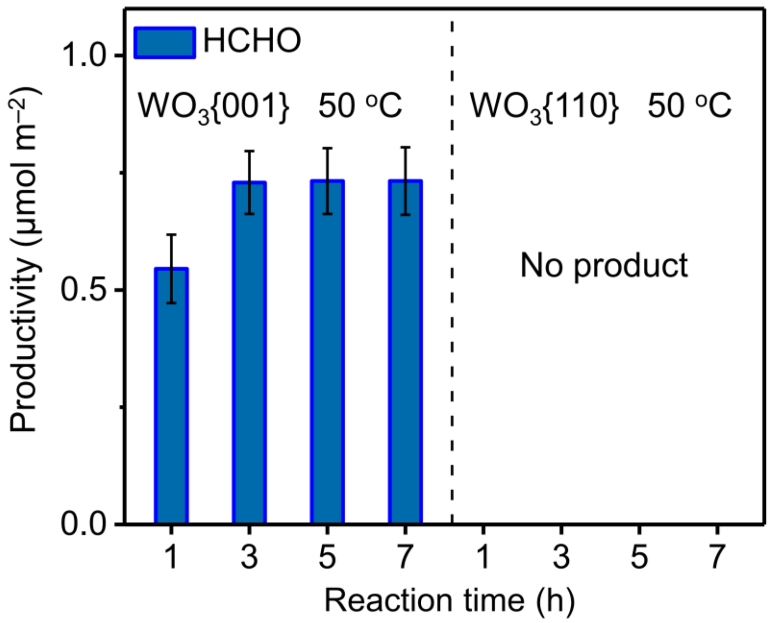

Supplementary Fig. 11 Photocatalytic CH_4 oxidation performance on WO_3\{001\} and WO_3\{110\} in CH_4 atmosphere with variation of reaction time at 50 ºC. Error bars indicate standard deviations.

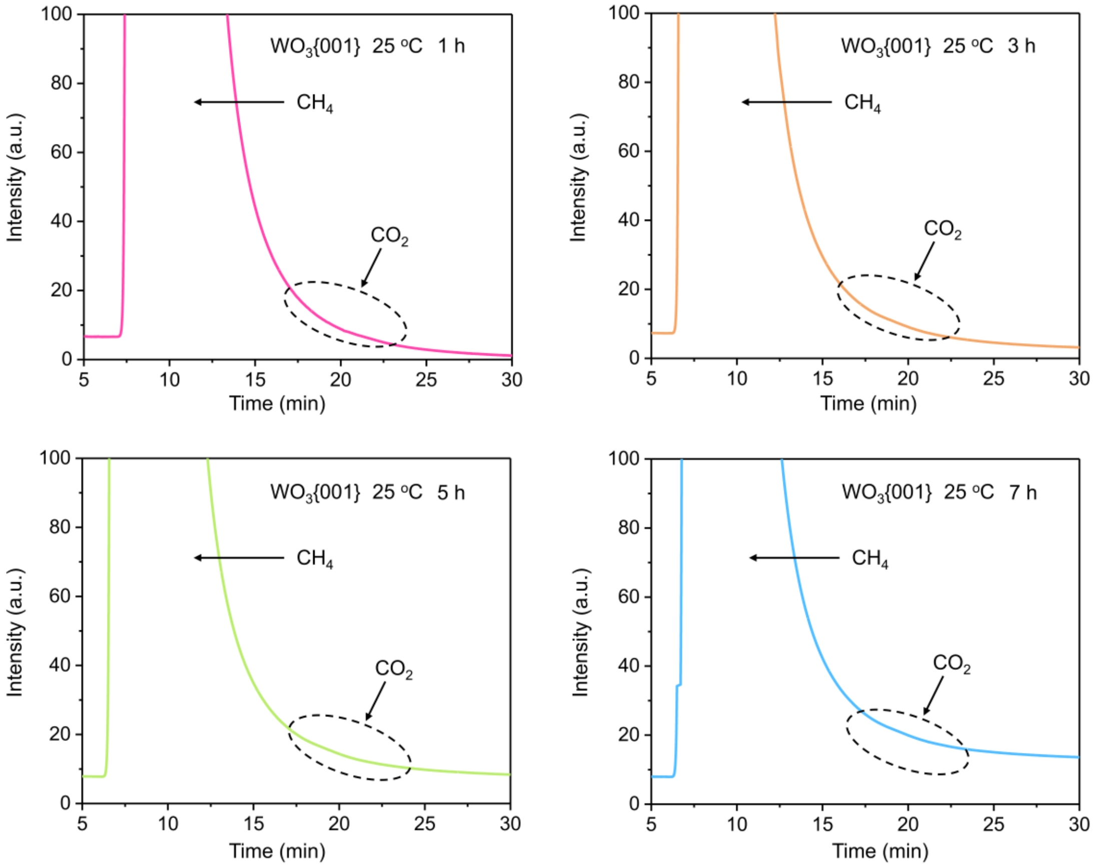

Supplementary Fig. 12 GC spectra of gas product from CH_4 oxidation on WO_3{001} in CH_4 atmosphere with different reaction time. Peaks at 9.21 and 19.11 min are attributed to residual CH_4 and produced CO_2, respectively. Reaction condition: 10 mg catalyst, 20 bar CH_4, 5 mL H_2O volume, Xenon light 150 mW cm^{-2}, reaction temperature 25 °C.

No signal of CO_2 is observed, which is attributed to the low concentration of HCHO.

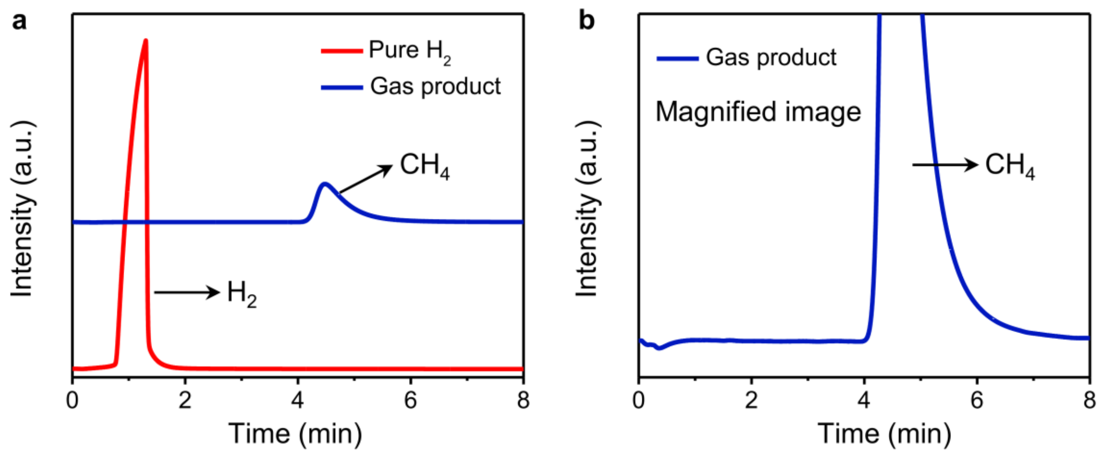

Supplementary Fig. 13 (a) GC signals of pure H_2 and gas product of WO_3 {001} after reaction in pure CH_4 atmosphere for 7 h under light irradiation. (b) Magnified image of gas product from Supplementary Fig. 13a.

No H_2 is generated in WO_3 {001} system after CH_4 oxidation reaction in absence of O_2. From the GC spectrum with thermal conductivity detector (TCD), taking pure H_2 as reference, the H_2 signal should be at 1.1 min. As shown in Supplementary Fig. 13a and the magnified image in Supplementary Fig. 13b, no H_2 signal is detected for the gas product over WO_3 {001} after reaction for 7 h in CH_4 atmosphere without O_2 addition. This result proves that no H_2 is generated.

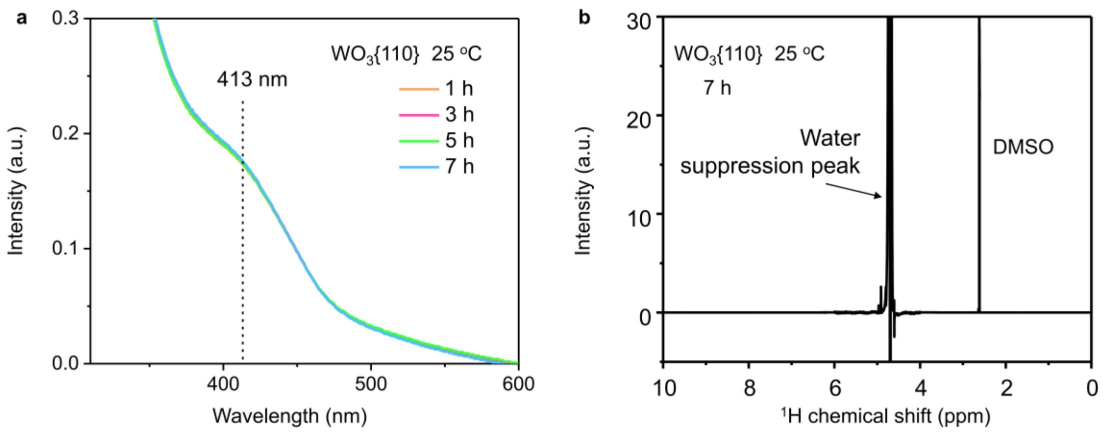

Supplementary Fig. 14 (a) UV-visible absorption spectra of HCHO product on WO_3 {110} in CH_4 atmosphere with different reaction time. (b) ^{1}H NMR spectrum of product on WO_3 {110} in CH_4 atmosphere for 7 h reaction time. Reaction condition: 10 mg catalyst, 20 bar CH_4, 5 mL H_2O volume, Xenon light 150 mW cm^{-2}, reaction temperature 25 ºC.

The acetylacetone color-developing reaction reveals that no HCHO is generated on WO_3 {110} in CH_4 atmosphere at 25 ºC (Supplementary Fig. 14a). Also, no other carbon oxygenates product is detected in ^{1}H NMR spectrum (Supplementary Fig. 14b).

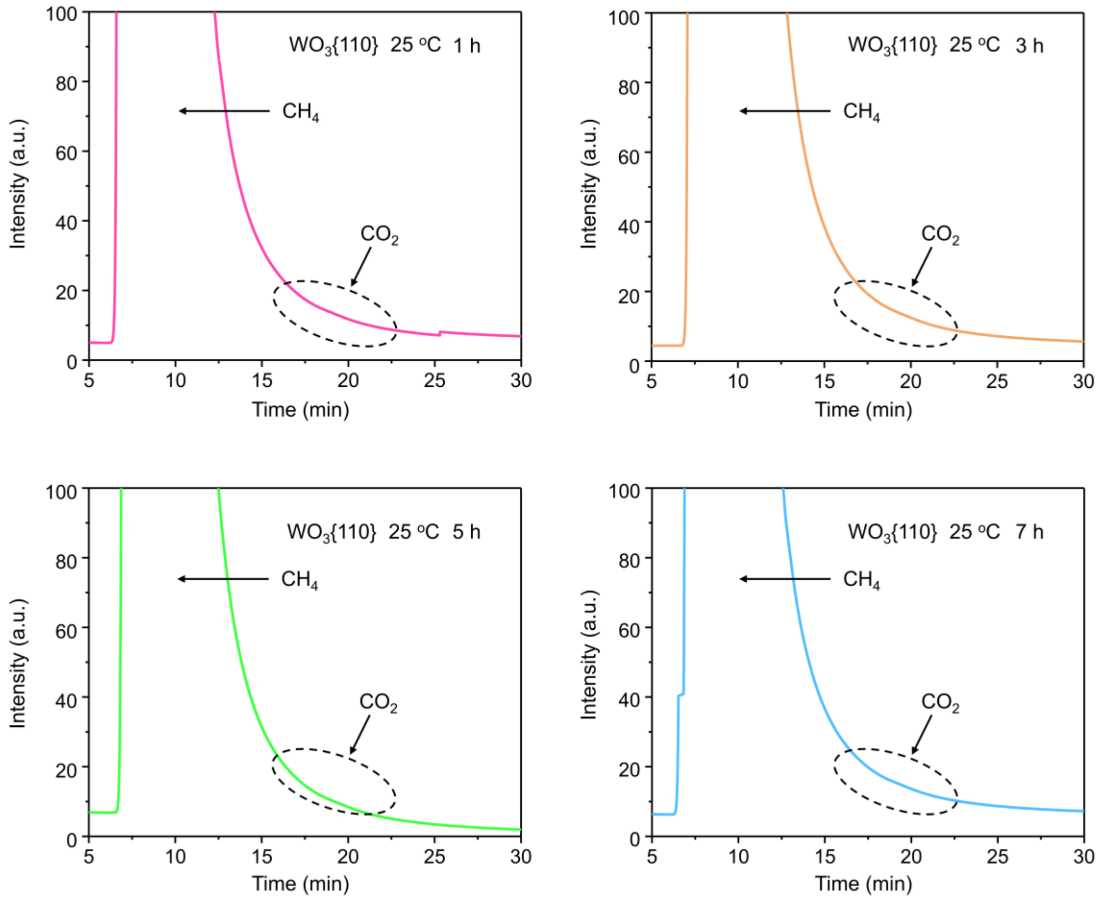

Supplementary Fig. 15 GC spectra of gas product from CH_4 oxidation on WO_3{110} in CH_4 atmosphere with different reaction time at 25 °C. Peaks at 9.21 and 19.11 min are attributed to residual CH_4 and produced CO_2, respectively. Reaction condition: 10 mg catalyst, 20 bar CH_4, 5 mL H_2O volume, Xenon light 150 mW cm^{-2}.

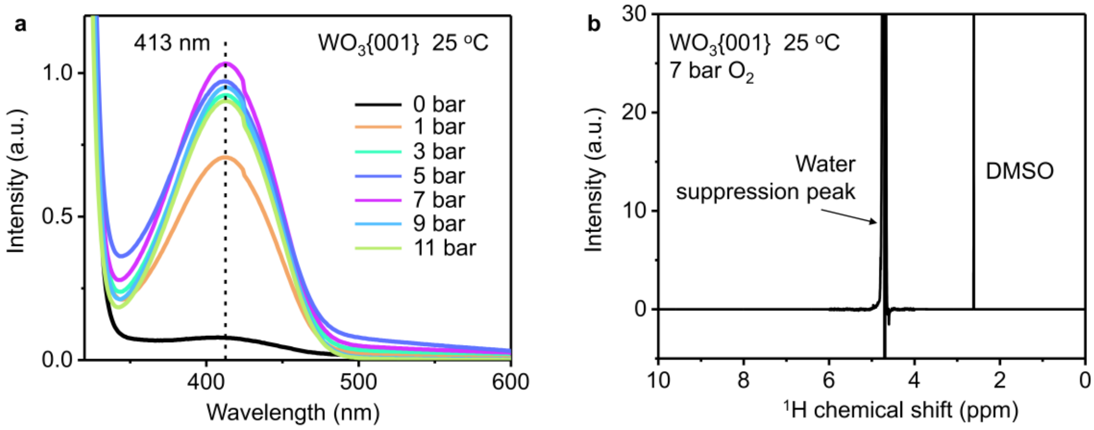

Supplementary Fig. 16 (a) UV-visible absorption spectra of HCHO product on WO_3 {001} in CH_4 + O_2 atmosphere of different O_2 pressure. (b) ^{1}H NMR spectrum of product on WO_3 {001} with 7 bar O_2 for 3 h reaction time. Reaction condition: 10 mg catalyst, total CH_4 + O_2 gas pressure 20 bar, 5 mL H_2O volume, 3 h reaction time, Xenon light 150 mW cm^{-2}, reaction temperature 25 °C.

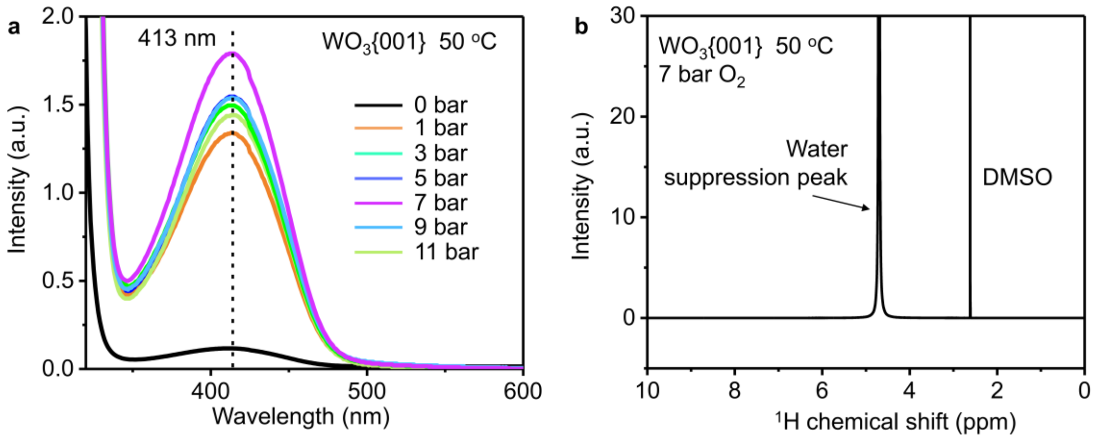

Supplementary Fig. 17 (a) UV-visible absorption spectra of HCHO product on WO_3\{001\} in $CH_4 + O_2$ atmosphere of different O_2 pressure. (b) ^{1}H NMR spectrum of product on WO_3\{001\} with 7 bar O_2 for 3 h reaction time. Reaction condition: 10 mg catalyst, total $CH_4 + O_2$ gas pressure 20 bar, 5 mL H_2O volume, 3 h reaction time, Xenon light 150 mW cm^{-2}, reaction temperature 50 °C.

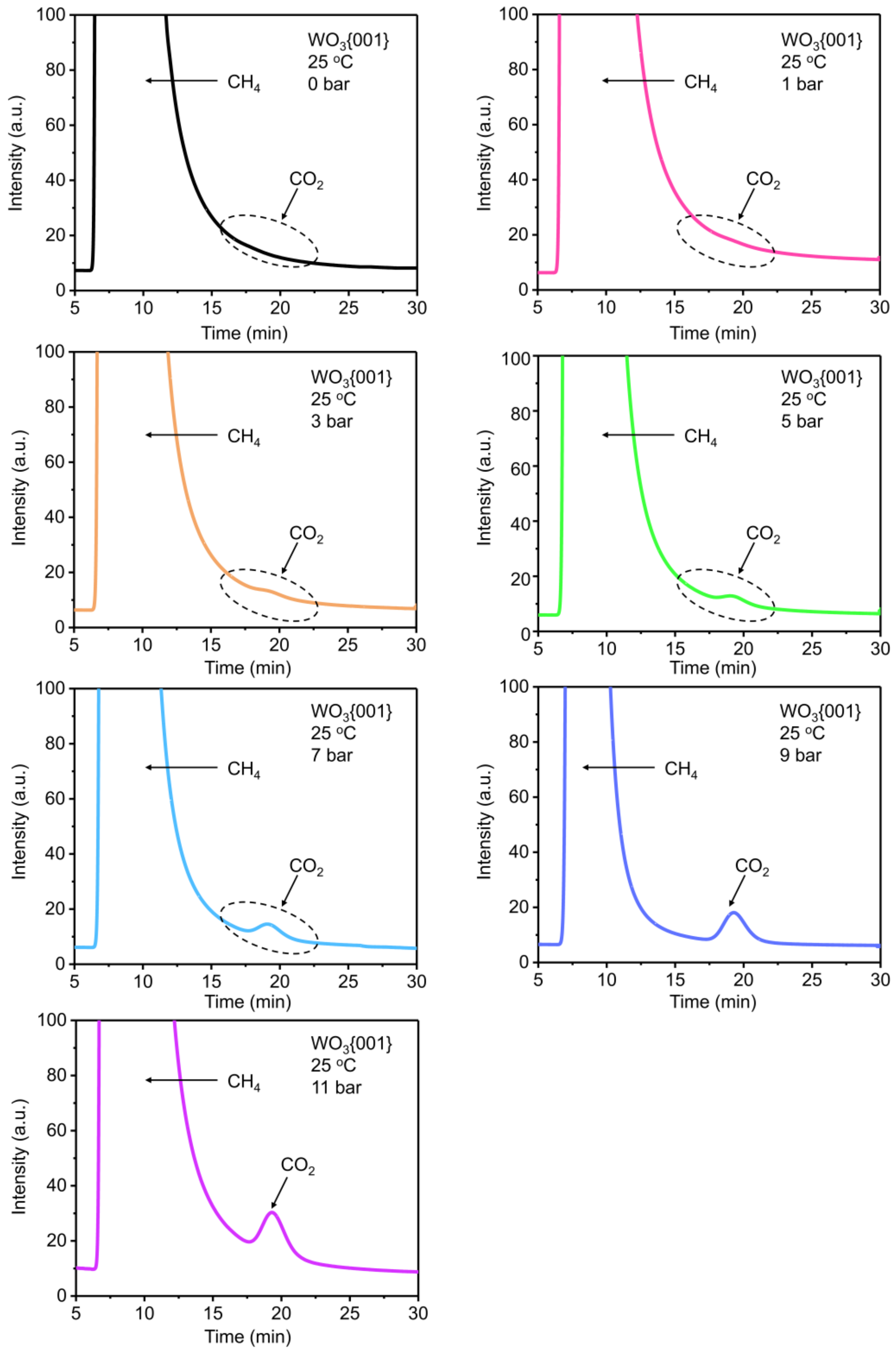

Supplementary Fig. 18 GC spectra of gas product from CH_4 oxidation on WO_3{001} in O_2 atmosphere of different pressure. Peaks at 9.21 and 19.11 min are attributed to residual CH_4 and produced CO_2, respectively. Reaction condition: 10 mg catalyst, total CH_4 + O_2 gas pressure 20 bar, 5 mL H_2O volume, 3 h reaction time, Xenon light 150 mW cm^{-2}, reaction temperature 25 °C.

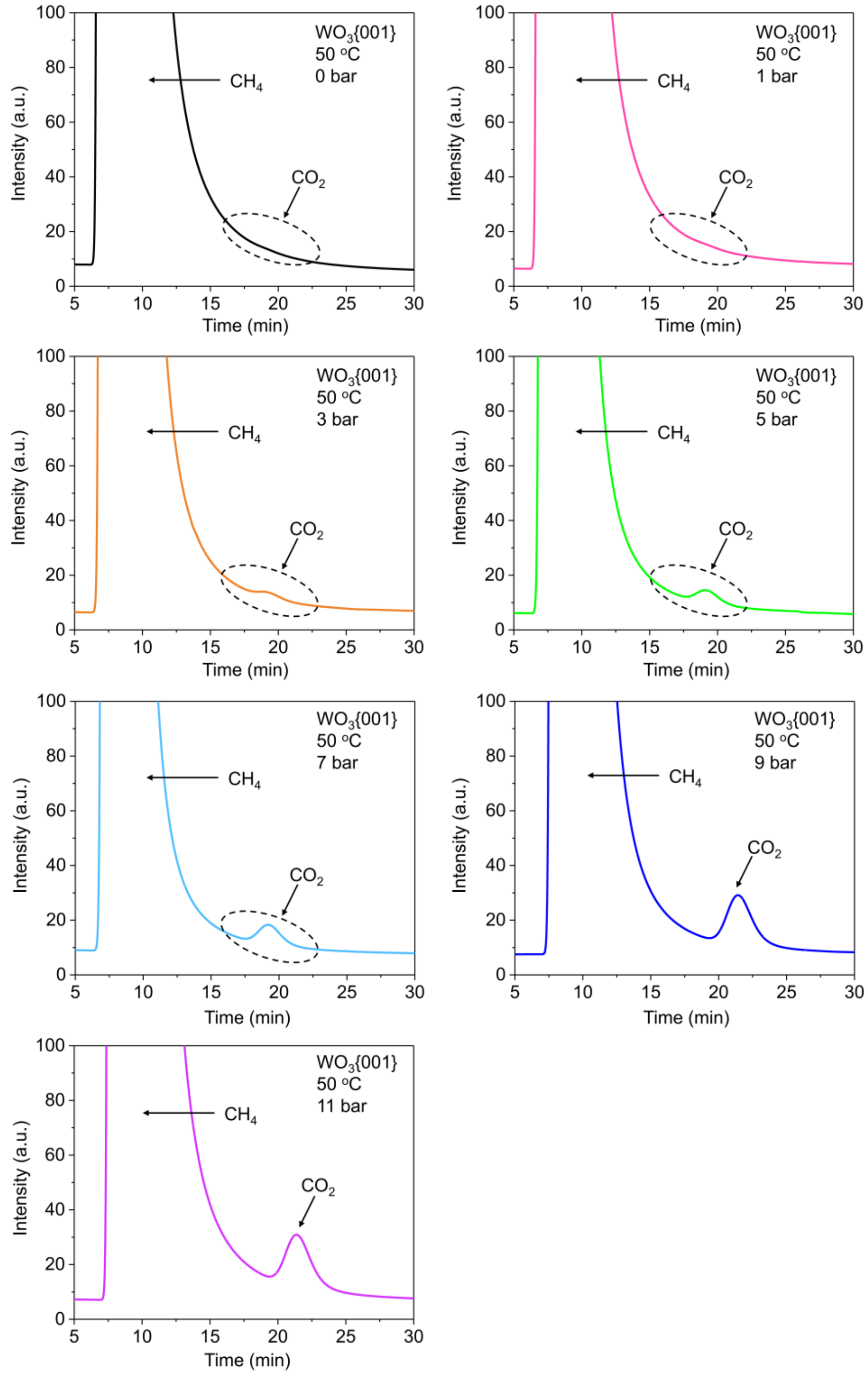

Supplementary Fig. 19 GC spectra of gas product from CH_4 oxidation on WO_3\{001\} in O_2 atmosphere of different pressure. Peaks at 9.21 and 19.11 min are attributed to residual CH_4 and produced CO_2, respectively. Reaction condition: 10 mg catalyst, total CH_4 + O_2 gas pressure 20 bar, 5 mL H_2O volume, 3 h reaction time, Xenon light 150 mW cm^{-2}, reaction temperature 50 °C.

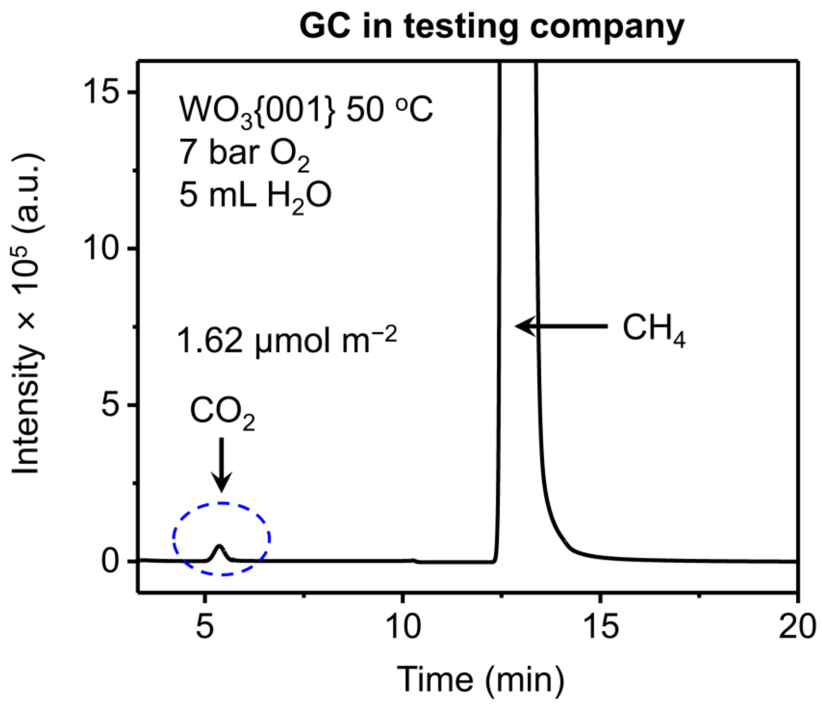

Supplementary Fig. 20 GC spectrum of gas product from CH_4 oxidation on WO_3 {001} tested in testing company. Peaks at 13.02 min and 5.37 min are attributed to residual CH_4 and produced CO_2, respectively. Reaction condition: 10 mg catalyst, 7 bar O_2, 13 bar CH_4, 5 mL H_2O volume, 3 h reaction time, Xenon light 150 mW cm^{-2}, reaction temperature 50 °C.

The GC baseline for CO_2 testing in our laboratory is not perfectly flat and a small overlap happens between the peaks of CH_4 and CO_2, which may lead to some deviation in CO_2 result. Actually, this deviation has been considered in the error bar as shown in Fig. 3. To further verify this result, the gas product on WO_3 {001} at 50 °C reaction temperature with 7 bar O_2 + 13 bar CH_4 and 5 mL H_2O (Supplementary Fig. 19) is retested in Shiyanjia Lab (testing company, www.shiyanjia.com). As shown in Supplementary Fig. 20, the baseline is perfectly flat and the peaks between CH_4 and CO_2 are completely separated. Through quantitative analysis, the productivity of CO_2 is 1.47 μmol m^{-2} (GC in our lab) and 1.62 μmol m^{-2} (GC in testing company), respectively, which is within the productivity range from 1.29 to 1.68 μmol m^{-2} with error bar (7 bar O_2, Fig. 3c). Thus, the CO_2 data of Fig. 3 is reasonable in our manuscript.

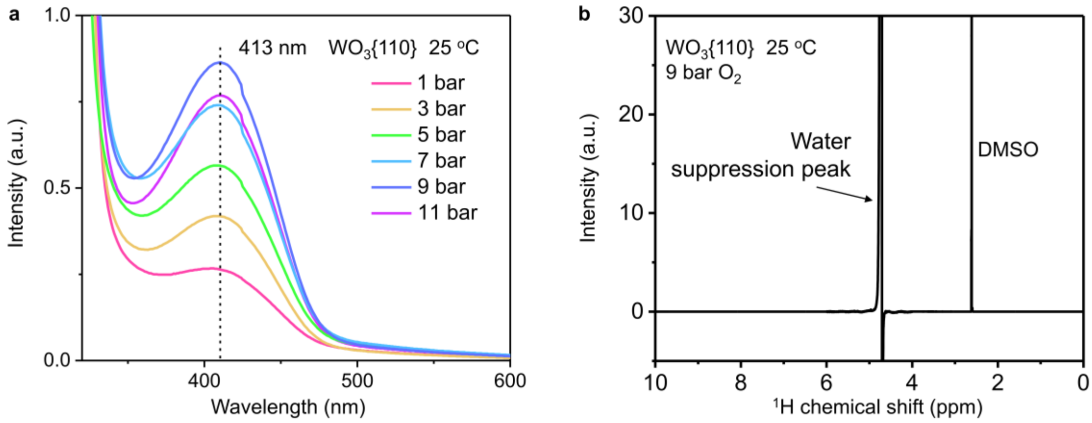

Supplementary Fig. 21 (a) UV-visible absorption spectra of HCHO product on WO_3{110} in CH_4 + O_2 atmosphere of different O_2 pressure. (b) ^{1}H NMR spectrum of product on WO_3{110} with 9 bar O_2 for 3 h reaction time. Reaction condition: 10 mg catalyst, total CH_4 + O_2 gas pressure 20 bar, 5 mL H_2O volume, 3 h reaction time, Xenon light 150 mW cm^{-2}, reaction temperature 25 °C.

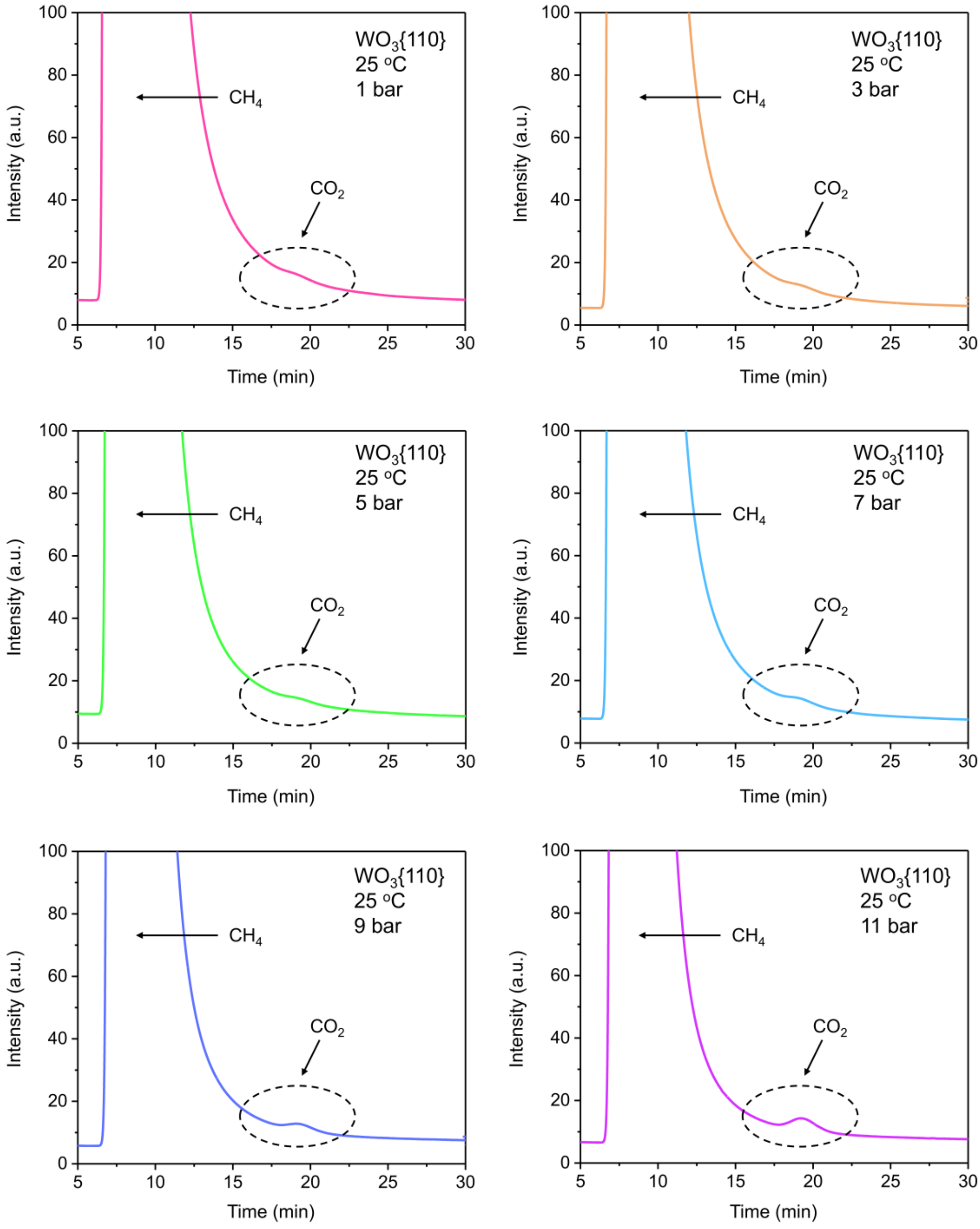

Supplementary Fig. 22 GC spectra of gas product from CH_4 oxidation on WO_3 {110} in O_2 atmosphere of different pressure. Peaks at 9.21 and 19.11 min are attributed to residual CH_4 and produced CO_2, respectively. Reaction condition: 10 mg catalyst, total CH_4 + O_2 gas pressure 20 bar, 5 mL H_2O volume, 3 h reaction time, Xenon light 150 mW cm^{-2}, reaction temperature 25 ºC.

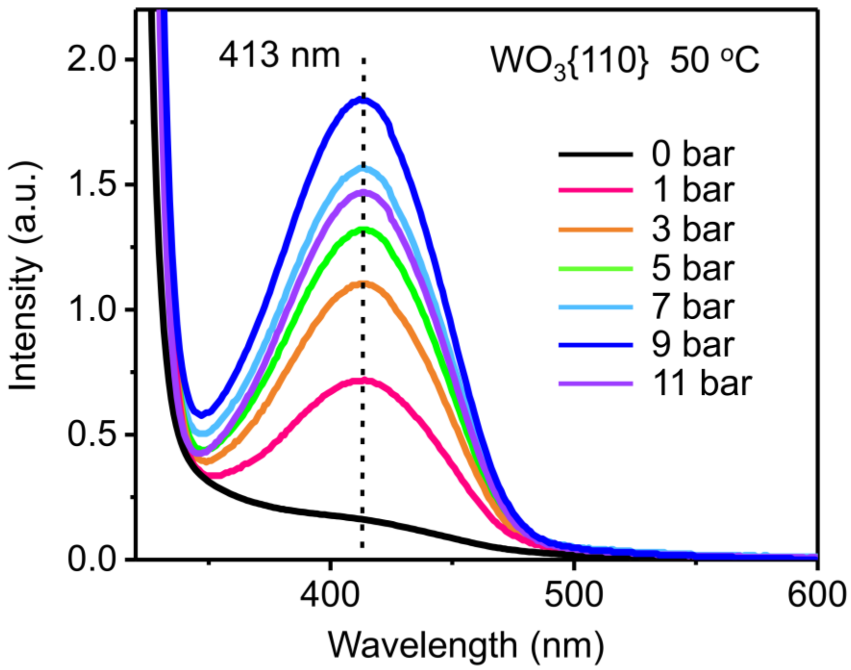

Supplementary Fig. 23 UV-visible absorption spectra of HCHO product on WO_3{110} in CH_4 + O_2 atmosphere of different O_2 pressure at 50 ºC. Reaction condition: 10 mg catalyst, total CH_4 + O_2 gas pressure 20 bar, 5 mL H_2O volume, 3 h reaction time, Xenon light 150 mW cm^{-2}.

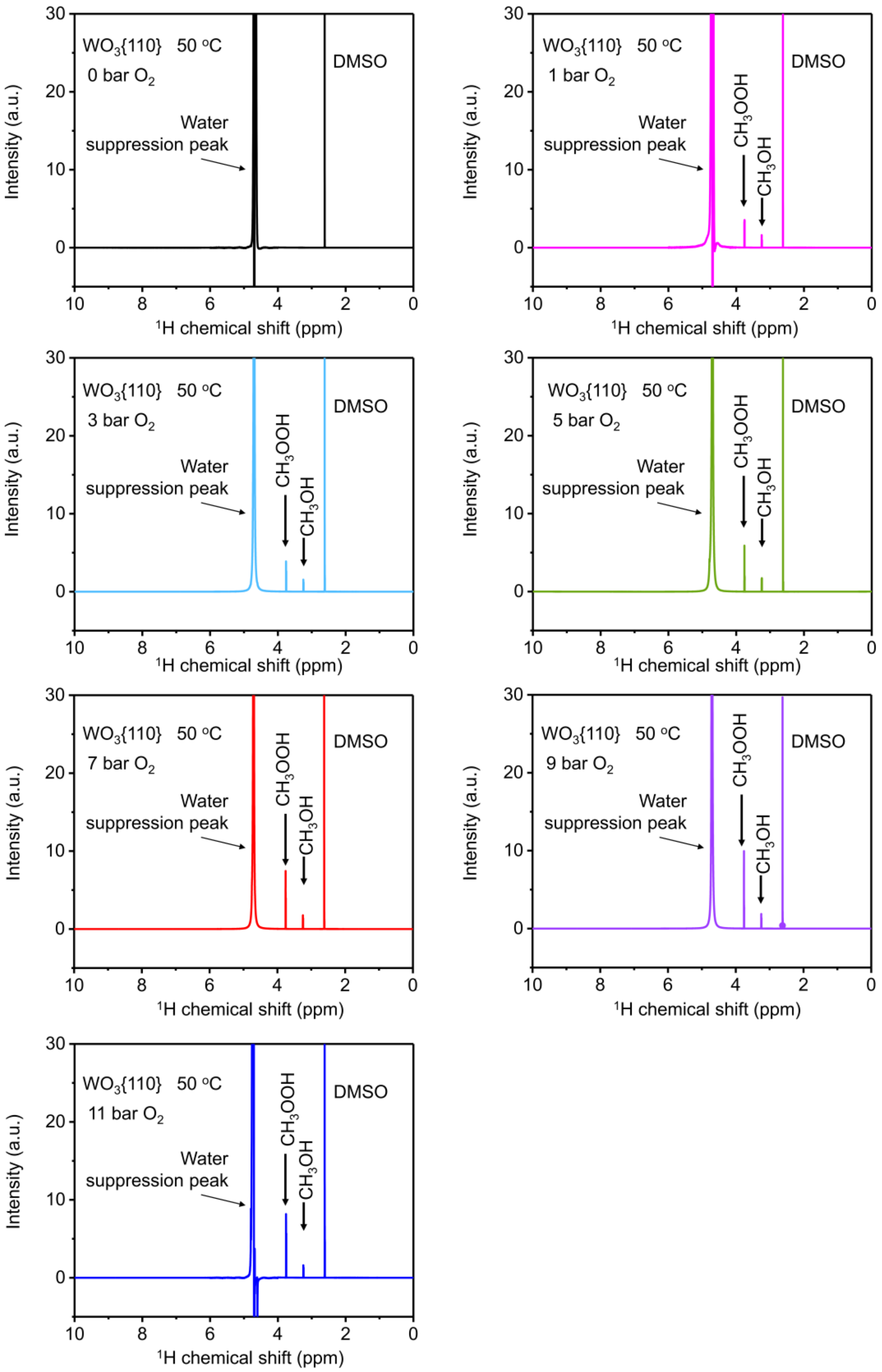

Supplementary Fig. 24 ^{1}H NMR spectra of product on $WO_3\{110\}$ in $CH_4 + O_2$ atmosphere of different O_2 pressure at 50 °C. Reaction condition: 10 mg catalyst, total $CH_4 + O_2$ gas pressure 20 bar, 5 mL H_2O volume, 3 h reaction time, Xenon light 150 mW cm^{-2}.

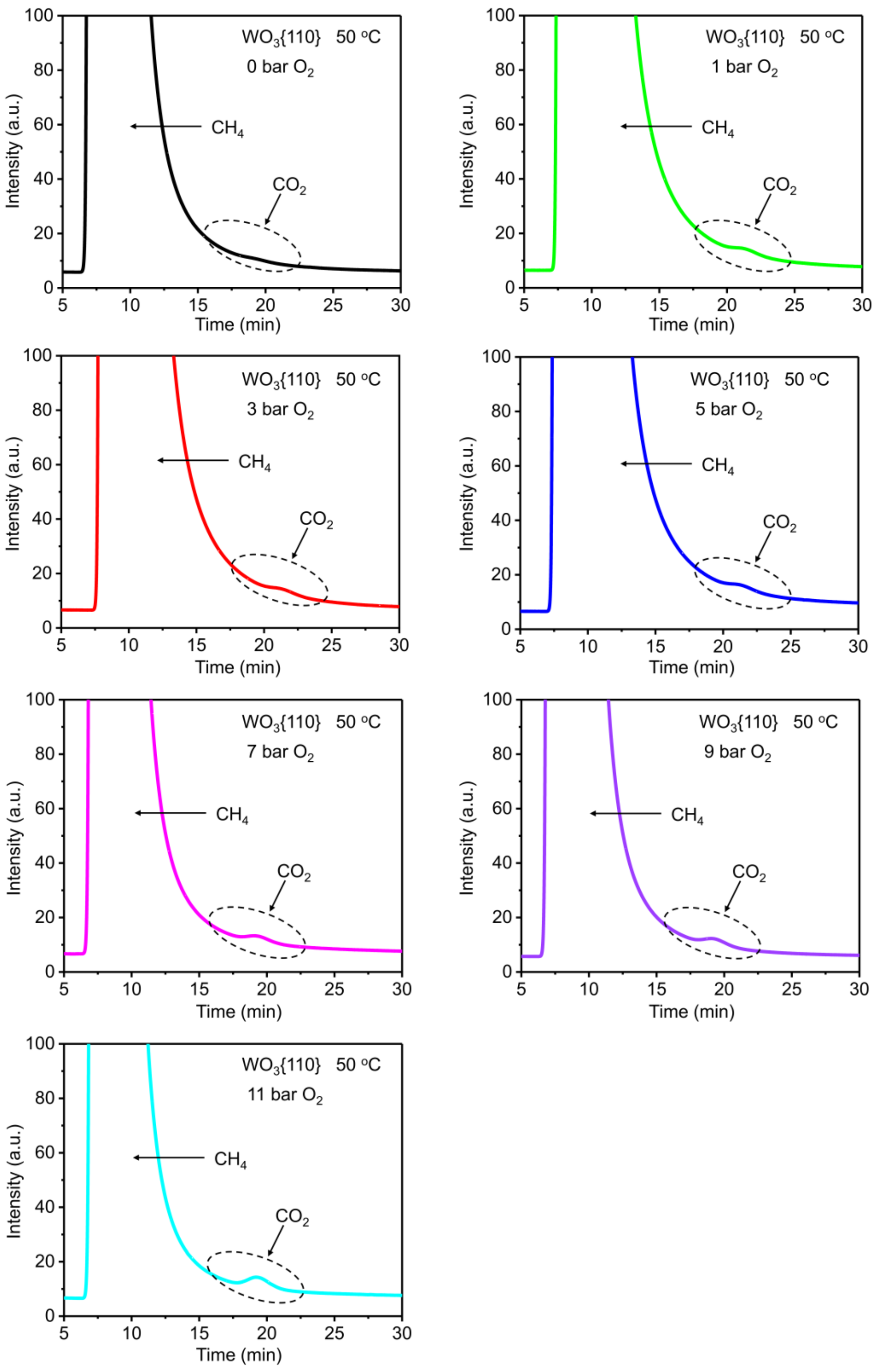

Supplementary Fig. 25 GC spectra of gas product from CH_4 oxidation on WO_3 {110} in $CH_4 + O_2$ atmosphere of different O_2 pressure at 50 °C. Peaks at 9.21 and 19.11 min are attributed to residual CH_4 and produced CO_2, respectively. Reaction condition: 10 mg catalyst, total $CH_4 + O_2$ gas pressure 20 bar, 5 mL H_2O volume, 3 h reaction time, Xenon light 150 mW cm^{-2}.

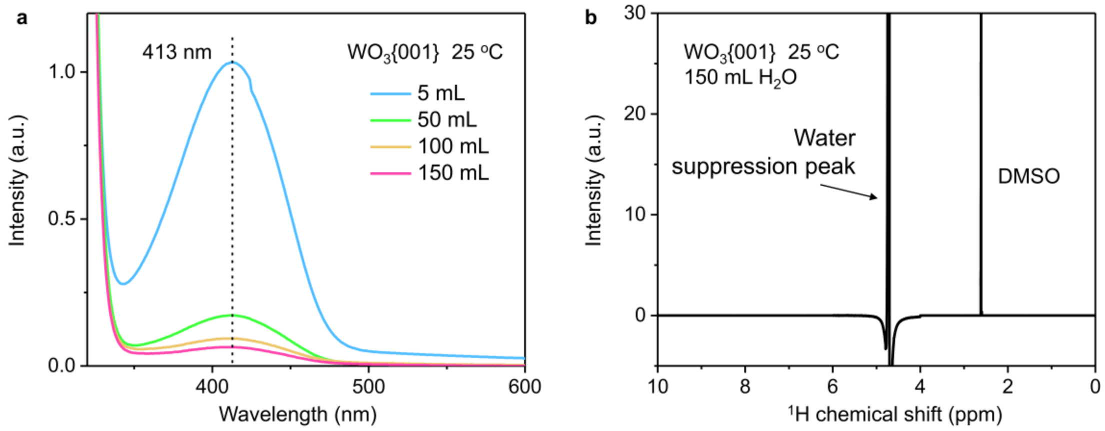

Supplementary Fig. 26 (a) UV-visible absorption spectra of HCHO product on WO_3 {001} in different volume of H_2O solvent. (b) ^{1}H NMR spectrum of product on WO_3 {001} in 150 mL H_2O under 7 bar O_2 pressure after 3 h reaction. Reaction condition: 10 mg catalyst, 7 bar O_2, 13 bar CH_4, 3 h reaction time, Xenon light 150 mW cm^{-2}, reaction temperature 25 °C.

The decrease of absorption peak intensity represents the reduced HCHO concentration.

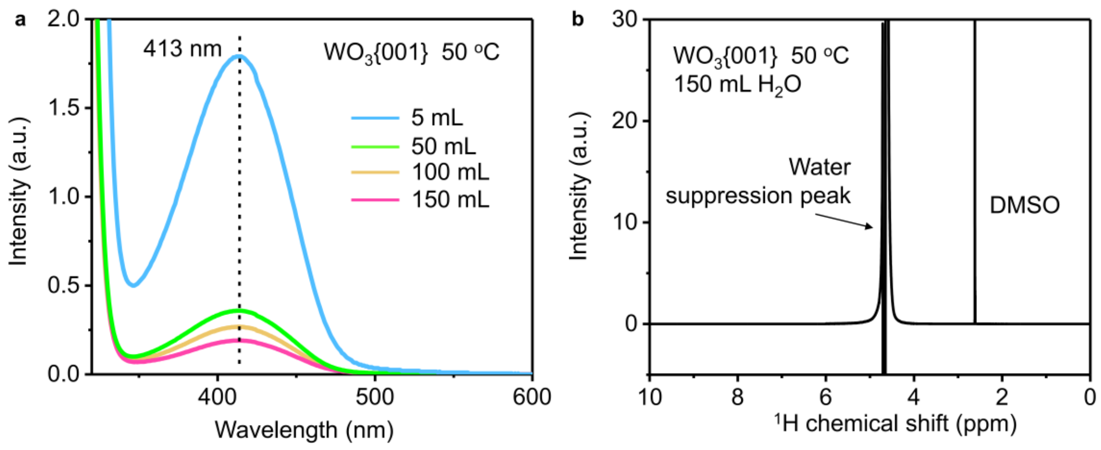

Supplementary Fig. 27 (a) UV-visible absorption spectra of HCHO product on WO_3 {001} in different volume of H_2O solvent. (b) ^{1}H NMR spectrum of product on WO_3 {001} in 150 mL H_2O under 7 bar O_2 pressure after 3 h reaction. Reaction condition: 10 mg catalyst, 7 bar O_2, 13 bar CH_4, 3 h reaction time, Xenon light 150 mW cm^{-2}, reaction temperature 50 °C.

The decrease of absorption peak intensity represents the reduced HCHO concentration.

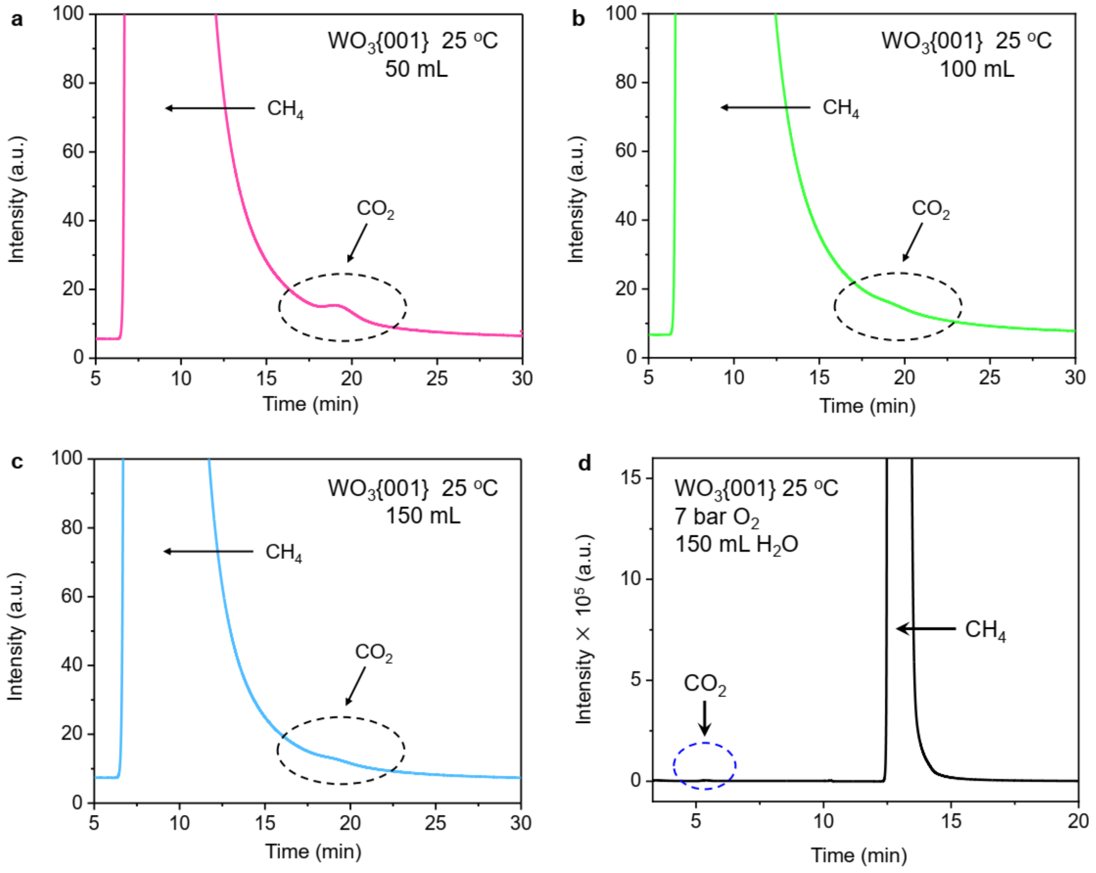

Supplementary Fig. 28 (a) GC spectra of gas product from CH_4 oxidation on WO_3{001} in H_2O volume of 50 mL, (b) 100 mL and (c) 150 mL. Peaks at 9.21 and 19.11 min are attributed to residual CH_4 and produced CO_2, respectively. Reaction condition: 10 mg catalyst, 7 bar O_2, 13 bar CH_4, 3 h reaction time, Xenon light 150 mW cm^{-2}, reaction temperature 25 °C. (d) Retest results in a testing company. GC spectrum of gas product from CH_4 oxidation on WO_3{001} with 150 mL H_2O at 25 °C

To determine whether the CO_2 signal truly disappears or is masked by the CH_4 peak as shown in Supplementary Fig. 28c, the gas products obtained with 150 mL H_2O are retested in Shiyanjia Lab. As shown in Supplementary Fig. 28d, no CO_2 signal is detected, indicating that no CO_2 is generated. Therefore, the HCHO selectivity over WO_3{001} with 150 mL H_2O can be regarded as 100%.

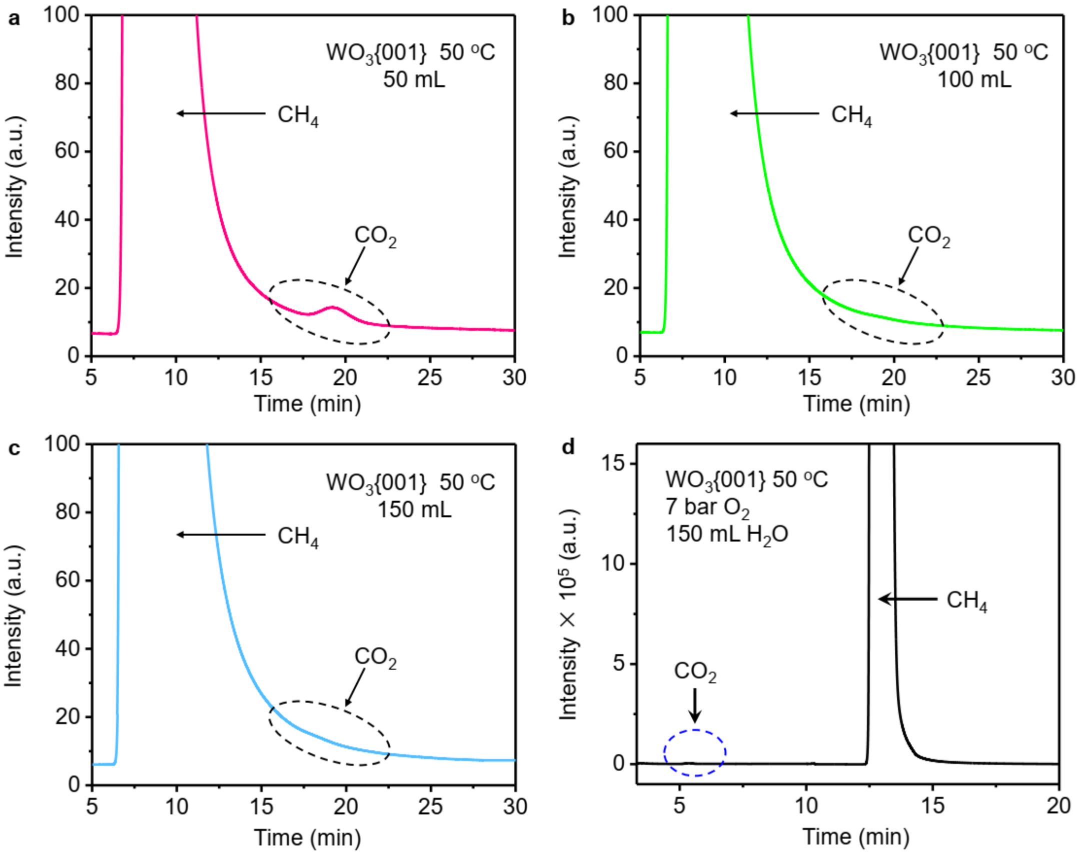

Supplementary Fig. 29 (a) GC spectra of gas product from CH_4 oxidation on WO_3 {001} in H_2O volume of 50 mL, (b) 100 mL and (c) 150 mL. Peaks at 9.21 and 19.11 min are attributed to residual CH_4 and produced CO_2, respectively. Reaction condition: 10 mg catalyst, 7 bar O_2, 13 bar CH_4, 3 h reaction time, Xenon light 150 mW cm^{-2}, reaction temperature 50 °C. (d) Retest results in a testing company. GC spectrum of gas product from CH_4 oxidation on WO_3 {001} with 150 mL H_2O at 50 °C

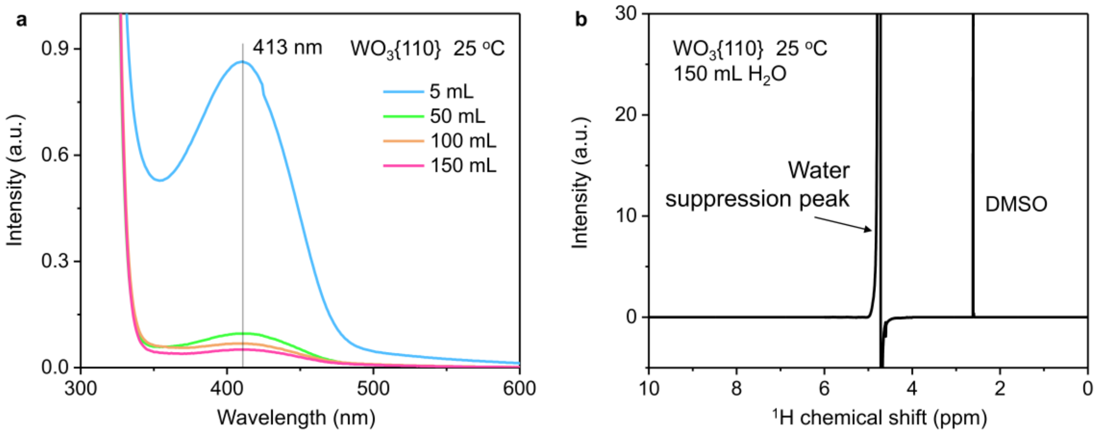

Supplementary Fig. 30 (a) UV-visible absorption spectra of HCHO product on WO_3 {110} in H_2O of different volume. (b) ^{1}H NMR spectrum of product on WO_3 {110} in 150 mL H_2O under 9 bar O_2 pressure after 3 h reaction. Reaction condition: 10 mg catalyst, 9 bar O_2, 11 bar CH_4, 3 h reaction time, Xenon light 150 mW cm^{-2}, reaction temperature 25 °C.

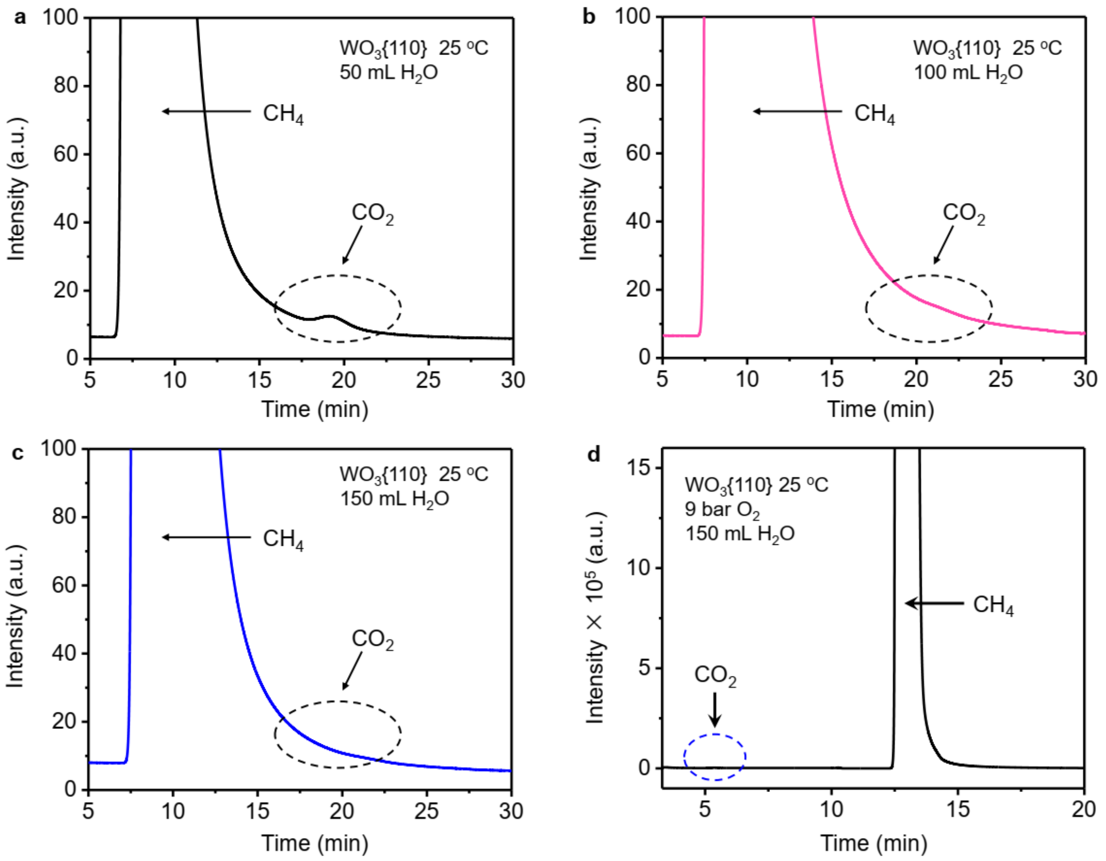

Supplementary Fig. 31 (a) GC spectra of gas product from CH_4 oxidation on WO_3{110} in H_2O volume of 50 mL, (b) 100 mL and (c) 150 mL. Peaks at 9.21 and 19.11 min are attributed to residual CH_4 and produced CO_2, respectively. Reaction condition: 10 mg catalyst, 9 bar O_2, 11 bar CH_4, 3 h reaction time, Xenon light 150 mW cm^{-2}, reaction temperature 25 °C. (d) Retest results in a testing company. GC spectrum of gas product from CH_4 oxidation on WO_3{110} with 150 mL H_2O at 25 °C

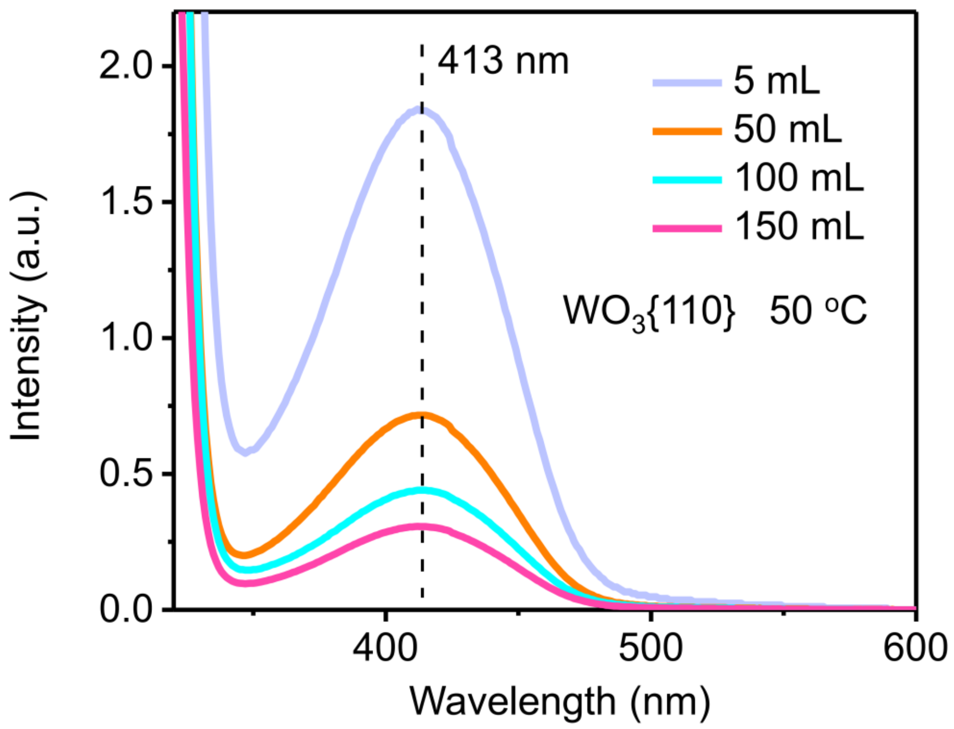

Supplementary Fig. 32 UV-visible absorption spectra of HCHO product on WO_3{110} in H_2O of different volume. Reaction condition: 10 mg catalyst, 9 bar O_2, 11 bar CH_4, 3 h reaction time, Xenon light 150 mW cm^{-2}, reaction temperature 50 ºC.

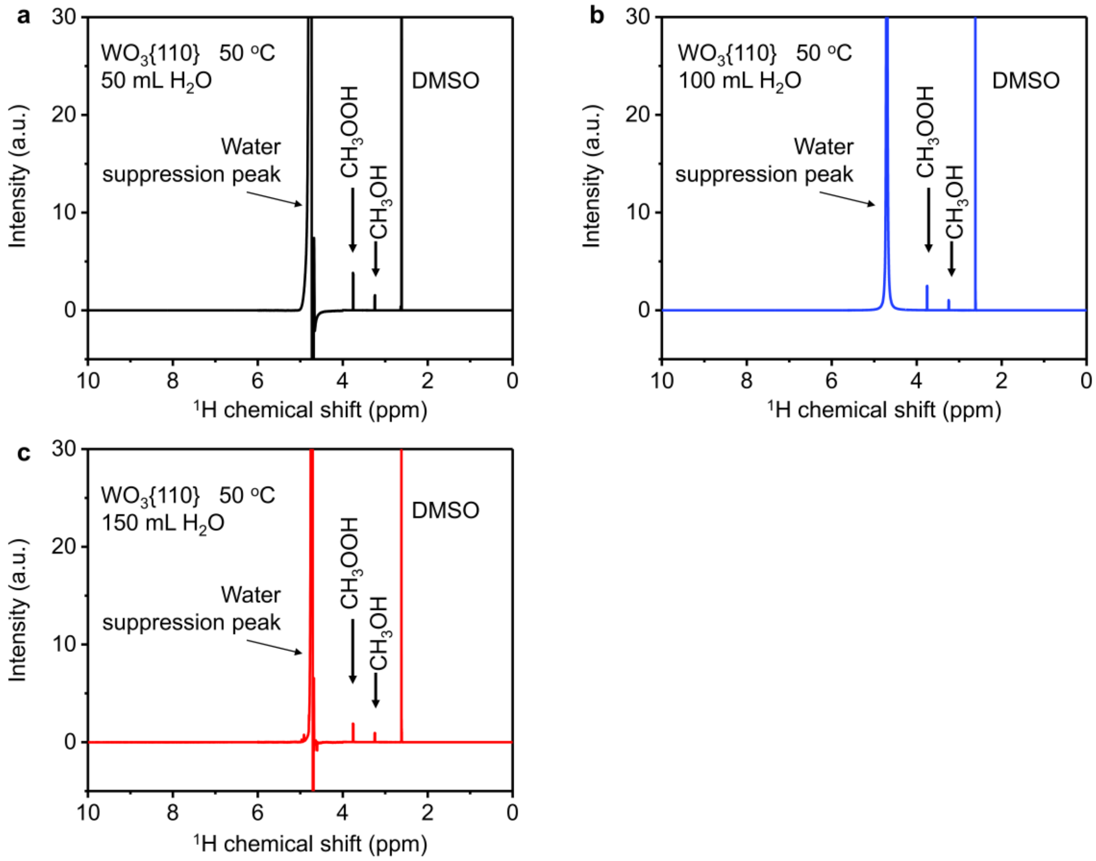

Supplementary Fig. 33 (a) ^{1}H NMR spectra of product on WO_3\{110\} in H_2O of different volume with 50 mL, (b) 100 mL and (c) 150 mL. Reaction condition: 10 mg catalyst, 9 bar O_2, 11 bar CH_4, 3 h reaction time, Xenon light 150 mW cm^{-2}, reaction temperature 50 ^{o}C.

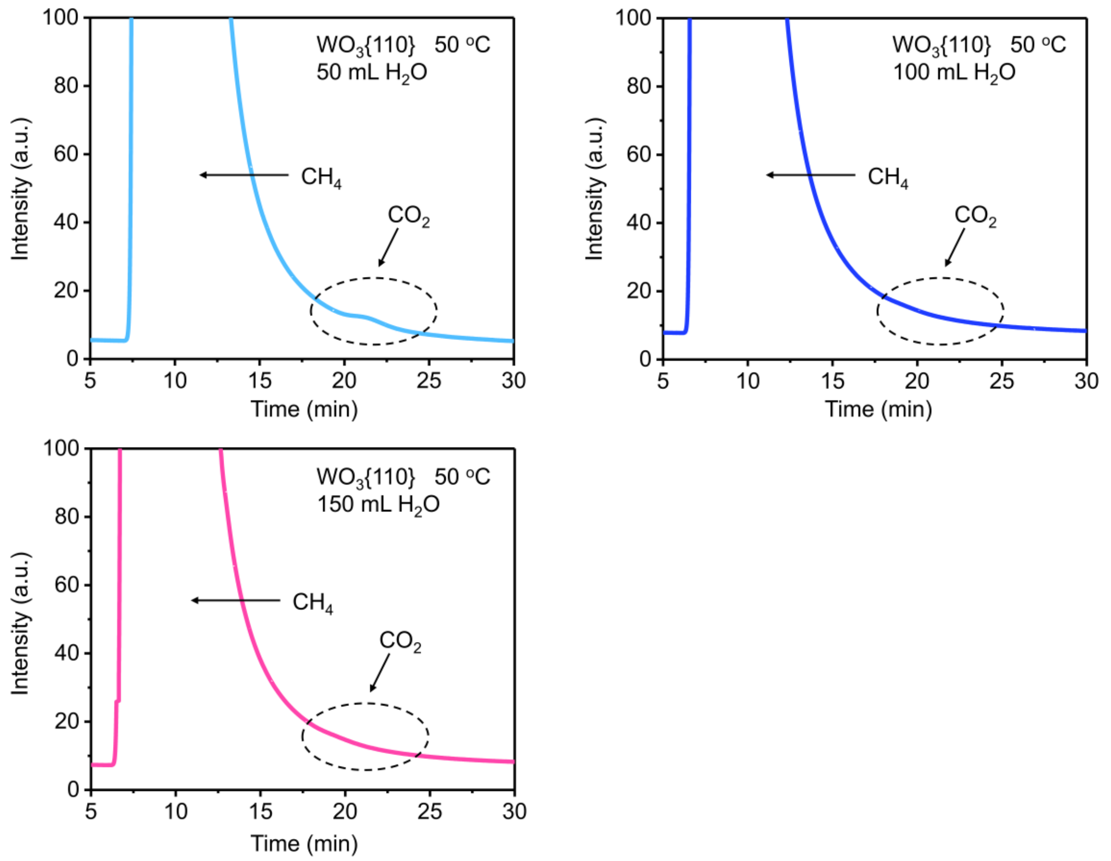

Supplementary Fig. 34 GC spectra of gas product from CH_4 oxidation over WO_3 {110} in H_2O of different volume. Peaks at 9.21 and 19.11 min are attributed to residual CH_4 and produced CO_2, respectively. Reaction condition: 10 mg catalyst, 9 bar O_2, 11 bar CH_4, 3 h reaction time, Xenon light 150 mW cm^{-2}, reaction temperature 50 ºC.

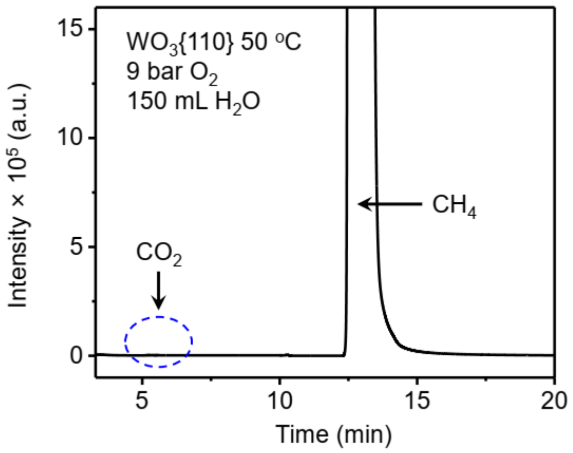

Supplementary Fig. 35 Retest results in a testing company. GC spectra of gas product from CH_4 oxidation on WO_3{110} with 150 mL H_2O at 50 °C. Reaction condition: 10 mg catalyst, 3 h reaction time, Xenon light 150 mW cm^{-2}, 9 bar O_2 + 11 bar CH_4.

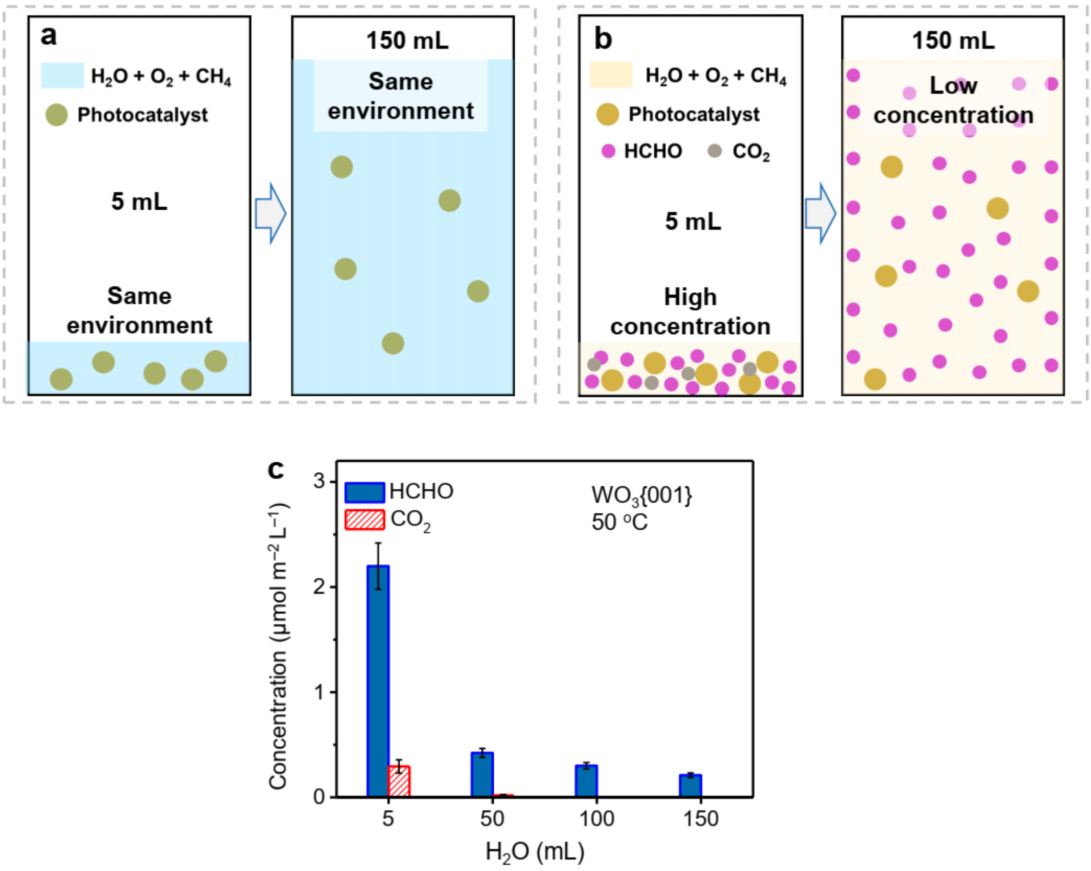

Supplementary Fig. 36 (a) No change happening on the reaction environment with the H_2O volume increasing from 5 to 150 mL. (b) Decreased concentration of both HCHO and photocatalyst with H_2O volume increasing from 5 to 150 mL, severely inhibiting the CO_2 generation. (c) Decreased oxygenates concentration on WO_3{001} at 50 ºC with variation of H_2O amount. Reaction condition: 10 mg catalyst, 3 h reaction time, Xenon light 150 mW cm^{-2}, 7 bar O_2 + 13 bar CH_4. Error bars indicate standard deviations.

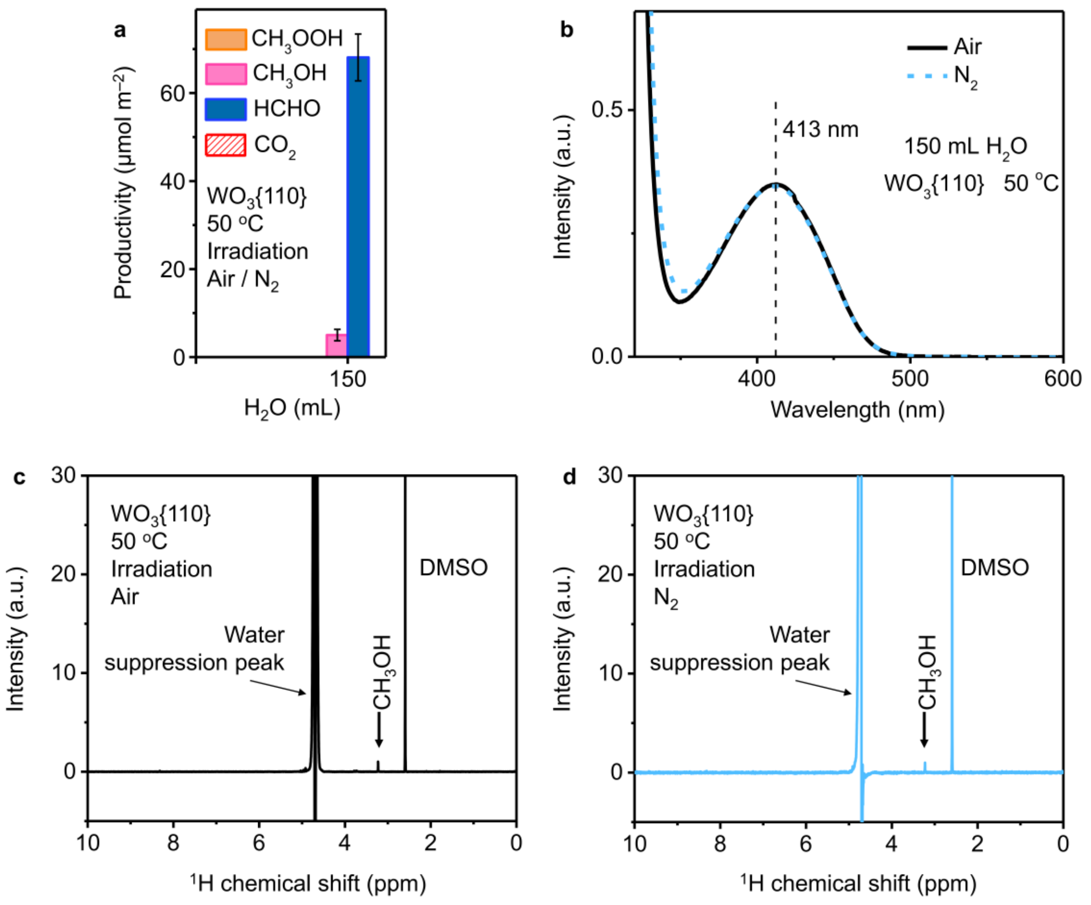

Supplementary Fig. 37 (a) The productivity of oxygenates generated over WO_3 {110} with 150 mL H_2O, 9 bar O_2 + 11 bar CH_4 at 50 °C, after light irradiation treatment under air or N_2 atmosphere. (b) The corresponding UV-visible absorption spectra of HCHO product and (c, d) ^{1}H NMR spectra after light irradiation treatment under air or N_2 atmosphere. Irradiation treatment: the product solution was centrifuged to remove the photocatalyst, and then exposed to Xenon light irradiation under air or N_2 atmosphere for 20 min. Error bars indicate standard deviations.

The HCHO selectivity enhancement along with H_2O volume increasing from 5 to 150 mL for both WO_3 {001} and WO_3 {110} does not involve the change of reaction mechanism. This is because 5 mL of H_2O is sufficient to disperse 10 mg WO_3 {001} or WO_3 {110} nanoparticles, and such adequate dispersion of photocatalyst in aqueous solution is maintained along with the volume of H_2O increasing to 150 mL (Supplementary Fig. 36a). Therefore, the change in H_2O volume only alter the concentration of reactants and products rather than the reaction pathway (mechanism). The reasons for the HCHO selectivity enhancement over WO_3 {001} and WO_3 {110} along with H_2O volume increasing are analyzed as follows.

For $WO_3\{001\}$ at both 25 °C and 50 °C reaction temperature, CH_4 can be oxidized directly to HCHO. Due to the small volume of 5 mL H_2O, the produced HCHO and photocatalyst of relatively high concentration possess the high possibility to collide with each other, leading to the further oxidation of HCHO to CO_2 (Supplementary Fig. 36b). When the H_2O volume increases to 150 mL, despite the total amount of HCHO increases (Fig. 3g), the concentration of both HCHO and photocatalyst decreases, severely inhibiting their collision and CO_2 generation (Supplementary Fig. 36c). Consequently, the selectivity of HCHO nearly reaches 100 % in 150 mL H_2O. Additionally, we note that the CO_2 signal disappearance is not equal to no any CO_2 product, because all the GC instruments have a detection limit. In short, the inhibition of CO_2 generation does not mean that the reaction mechanism is changed.

For $WO_3\{110\}$, owing to the existence of CH_3OOH and CH_3OH as intermediates, the selectivity enhancement of HCHO along with H_2O volume increasing should be separately discussed at 25 °C and 50 °C. The CH_4 oxidation pathway on $WO_3\{110\}$ is $CH_4 \rightarrow CH_3OOH \rightarrow HCHO\ (CH_3OH) \rightarrow CO_2$, where the production of HCHO may be accompanied by CH_3OOH and CH_3OH as intermediates. The signal disappearance and appearance of CH_3OOH and CH_3OH at 25 °C and 50 °C, respectively, are attributed to their rate coefficients (k^*) of CH_4 oxidation. The k^* of $WO_3\{110\}$ with radical mechanism exhibits a non-Arrhenius dependence and increases with the reaction temperature (equation S4).

At low temperature of 25 °C, the high $E_a{}^*$ value leads to slow k^* and low productivity of oxygenates (HCHO, 6.19 μmol m^{-2} within 5 mL H_2O). Amongst such low yield of oxygenates, it is reasonable to miss the intermediate signals of CH_3OOH and CH_3OH. Similar to $WO_3\{001\}$ system, along with H_2O volume increasing, the concentrations of oxygenates become even lower, thus the intermediate signals of CH_3OOH and CH_3OH are more unlikely to appear. Moreover, the reduced concentration of HCHO avoids its overoxidation. Ultimately, the selectivity of HCHO on $WO_3\{110\}$ reaches 100% at 25 °C.

When the reaction temperature rises to 50 °C, the k^* of $WO_3\{110\}$ is considerably promoted with the productivity of oxygenates reaching 16.88 μmol m^{-2} within 5 mL H_2O. The promoted reaction rate k^* of $WO_3\{110\}$ at 50 °C also accelerates the formation of intermediate products, contributing to the appearance of CH_3OOH and CH_3OH signals. Along with H_2O volume increasing from 5 to 150 mL, all

productivities of CH_3OOH, HCHO and CH_3OH are boosted. Meanwhile, the HCHO selectivity is enhanced from 58.01% (5 mL H_2O) to 76.88% (150 mL H_2O,) indicating that HCHO is always the primary product. This is because, based on the Gibbs free energy[31], the conversion of CH_3OOH to HCHO is thermodynamically favorable. Furthermore, it is found that CH_3OOH spontaneously and rapidly decomposes to HCHO under light irradiation without photocatalyst no matter in air or N_2 atmosphere. As shown in Fig. 3i, Supplementary Fig. 32 and 33c, the sum of the productivities of CH_3OOH + HCHO is ~ 68.54 μmol m^{-2} (150 mL). After light irradiation treatment without photocatalyst no matter in air or N_2 atmosphere (Supplementary Fig. 37), the signal of CH_3OOH disappears (Supplementary Fig. 37a, 37c and 37d) while the productivity of HCHO increases to ~ 68.41 μmol m^{-2} (Supplementary Fig. 37a and 37b). Thus, we can deduce that CH_3OOH is readily decomposed to HCHO, which is thermodynamically and kinetically favorable. Whereas, $CH_3OOH \rightarrow CH_3OH$ is an electron reduction process, which is relatively unfavorable in thermodynamics and dynamics. Therefore, along with H_2O volume increasing, the increase in the productivity of HCHO is greater than CH_3OOH and CH_3OH. The reason for the CO_2 signal disappearance in WO_3 {110} system is the same as WO_3 {001} system. Altogether, with the increase of H_2O amount, the productivity and selectivity of HCHO is obviously improved.

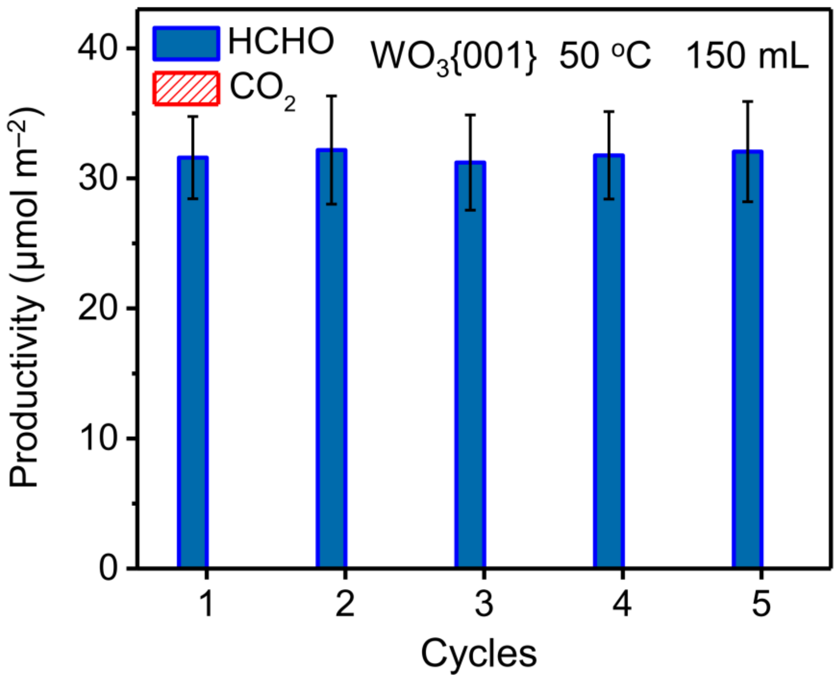

Supplementary Fig. 38 Stability of WO_3\{001\} for CH_4 oxidation. Reaction condition: 10 mg catalyst, 150 mL H_2O, 7 bar O_2 + 13 bar CH_4, 3 h reaction time of each cycle, Xenon light 150 mW cm^{-2}, 50 °C. Error bars indicate standard deviations.

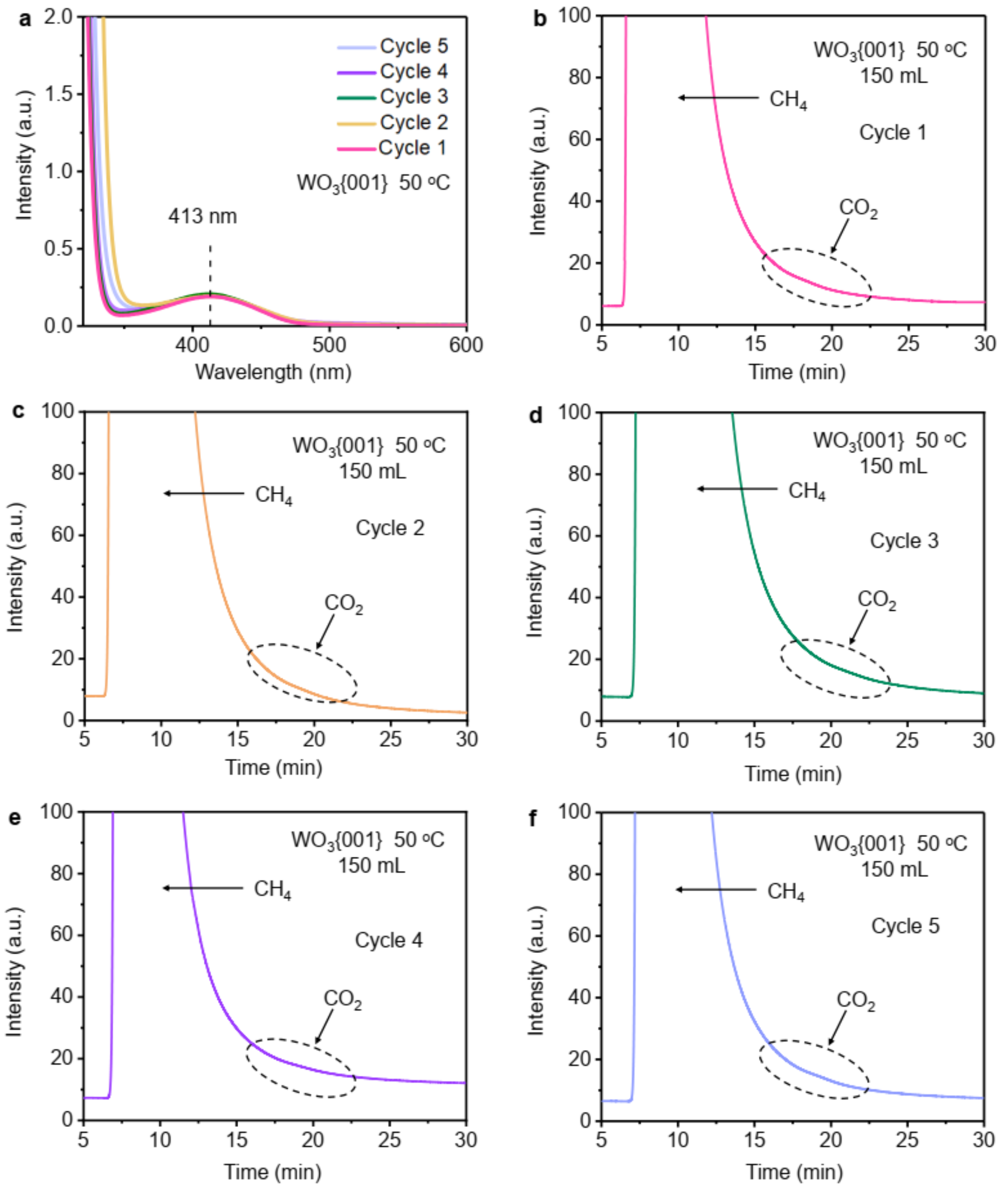

Supplementary Fig. 39 (a) UV-visible absorption spectra of HCHO product on WO_3 {001} for each cycle reaction. (b-f) GC spectra of gas product from CH_4 oxidation on WO_3 {001} for each cycle reaction. Reaction condition: 10 mg catalyst, 150 mL H_2O, 7 bar O_2 + 13 bar CH_4, 3 h reaction time of each cycle, Xenon light 150 mW cm^{-2}, 50 °C.

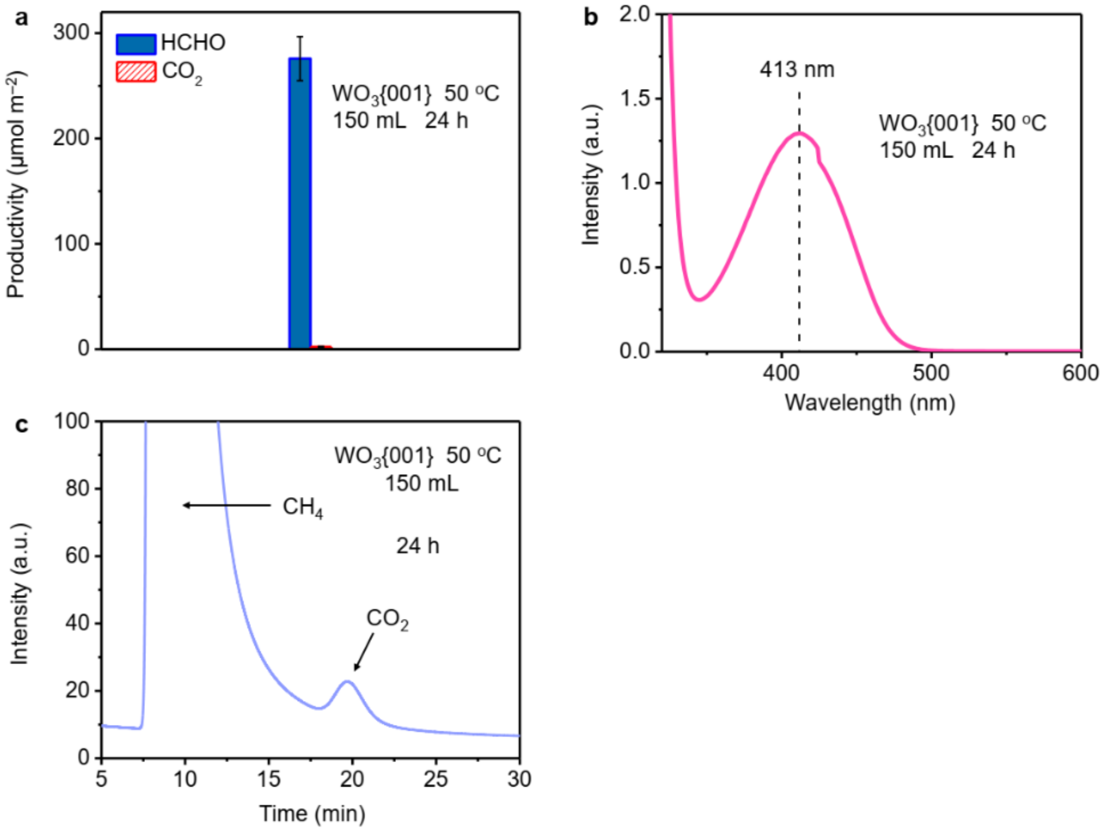

Supplementary Fig. 40 (a) Photocatalytic CH_4 oxidation performance on WO_3{001} for 24 h reaction time. (b) The corresponding UV-visible absorption spectrum of HCHO product and (c) GC spectrum of gas product. Reaction condition: 10 mg catalyst, 150 mL H_2O, 7 bar O_2 + 13 bar CH_4, 24 h reaction time, Xenon light 150 mW cm^{-2}, 50 °C. Error bars indicate standard deviations.

To prove the 100% replenishment of lattice-O for WO_3{001} from surrounding O_2, the stability of the photocatalyst is evaluated. After five photocatalytic cycles, the initial activity of WO_3{001} is almost fully preserved (Supplementary Fig. 38 and 39). Besides, by extending the reaction time to 24 h, the productivity of oxygenates is increased by ~8.8 times (HCHO, 275.85 μmol m^{-2}; CO_2, 2.3 μmol m^{-2}, Supplementary Fig. 40) compared with the reaction for 3 h (HCHO, 31.59 μmol m^{-2}; CO_2, 0 μmol m^{-2}). Both cyclic test and long-term reaction reveal the satisfied performance stability of WO_3{001} and again verify the 100% replenishment of lattice-O on WO_3{001} from surrounding O_2.

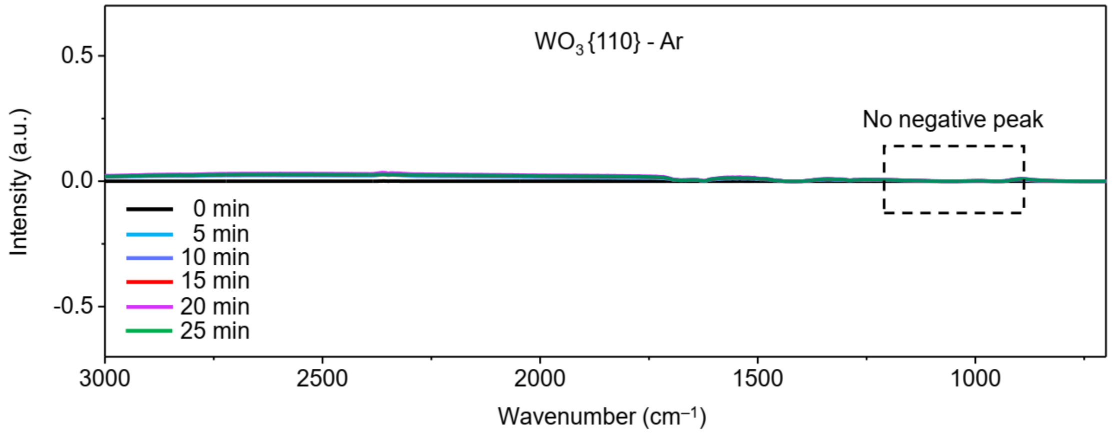

Supplementary Fig. 41 *In situ* DRIFTS spectra of WO_3 {110} in pure Ar atmosphere under different light irradiation time without H_2O addition.

To rule out lattice-O loss from photocorrosion, the *in situ* DRIFTS spectra of WO_3 {110} are carried out in pure Ar atmosphere with different irradiation time. As shown in Supplementary Fig. 41, no negative peak is observed upon the light irradiation from 0 to 25 min. Thus, the lattice-O loss of WO_3 {110} in CH_4 atmosphere without H_2O or with H_2O is attributed to its reaction with CH_4 or H_2O rather than the photocorrosion.

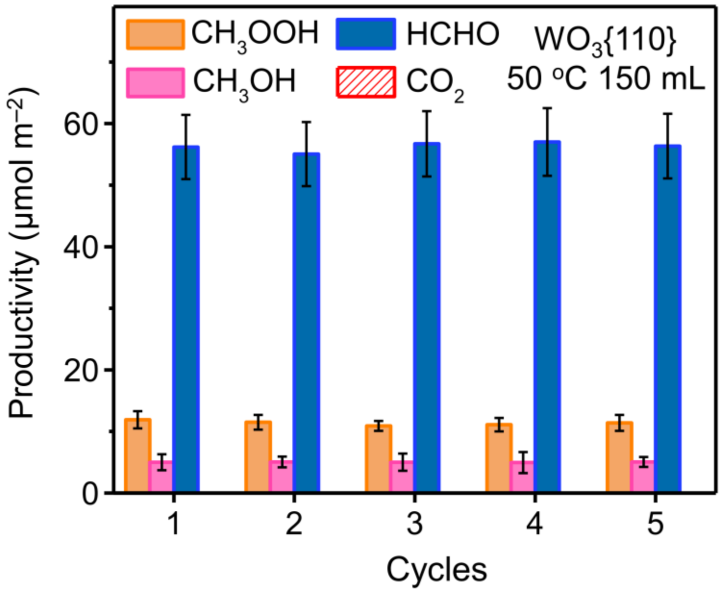

Supplementary Fig. 42 Stability of WO_3{110} for CH_4 oxidation. Reaction condition: 10 mg catalyst, 150 mL H_2O, 9 bar O_2 + 11 bar CH_4, 3 h reaction time of each cycle, Xenon light 150 mW cm^{-2}, 50 ºC. Error bars indicate standard deviations.

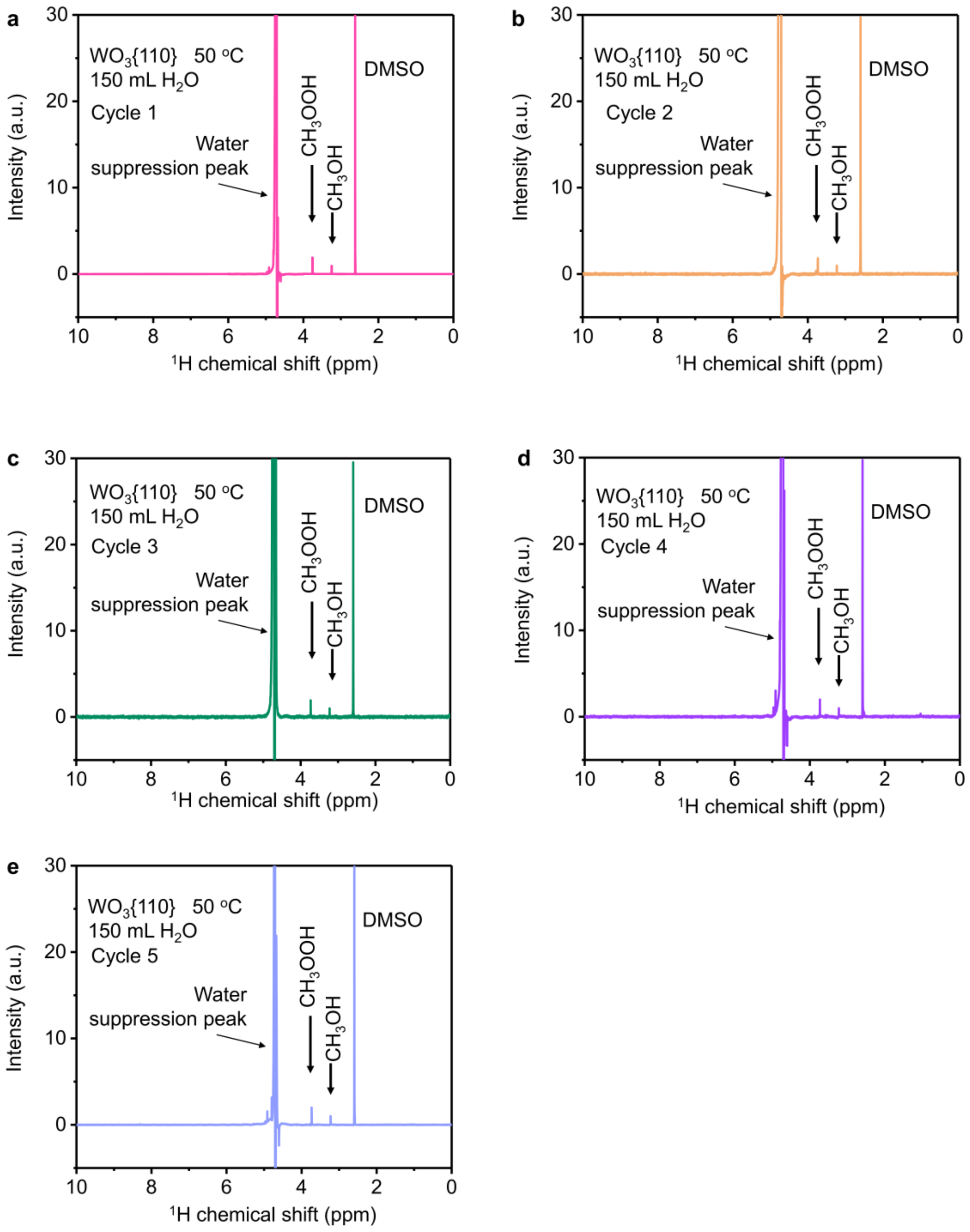

Supplementary Fig. 43 (a-e) ^{1}H NMR spectra of products on WO_3 {110} for each cycle reaction. Reaction condition: 10 mg catalyst, 150 mL H_2O, 9 bar O_2 + 11 bar CH_4, 3 h reaction time of each cycle, Xenon light 150 mW cm^{-2}, 50 °C.

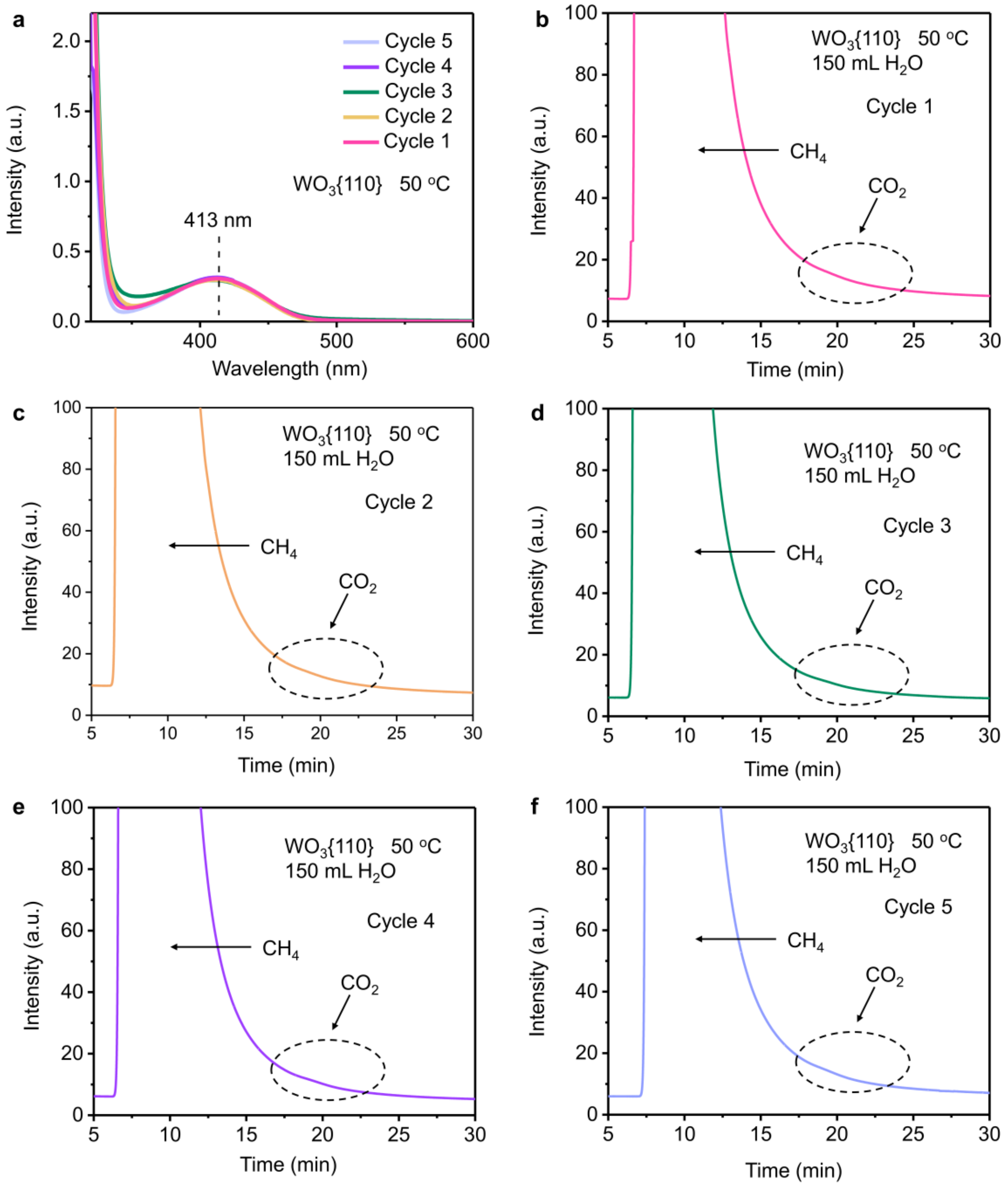

Supplementary Fig. 44 (a) UV-visible absorption spectra of HCHO product on $WO_3\{110\}$ for each cycle reaction. (b-f) GC spectra of gas product from CH_4 oxidation on $WO_3\{110\}$ for each cycle reaction. Reaction condition: 10 mg catalyst, 150 mL H_2O, 9 bar O_2 + 11 bar CH_4, 3 h reaction time of each cycle, Xenon light 150 mW cm^{-2}, 50 ^oC.

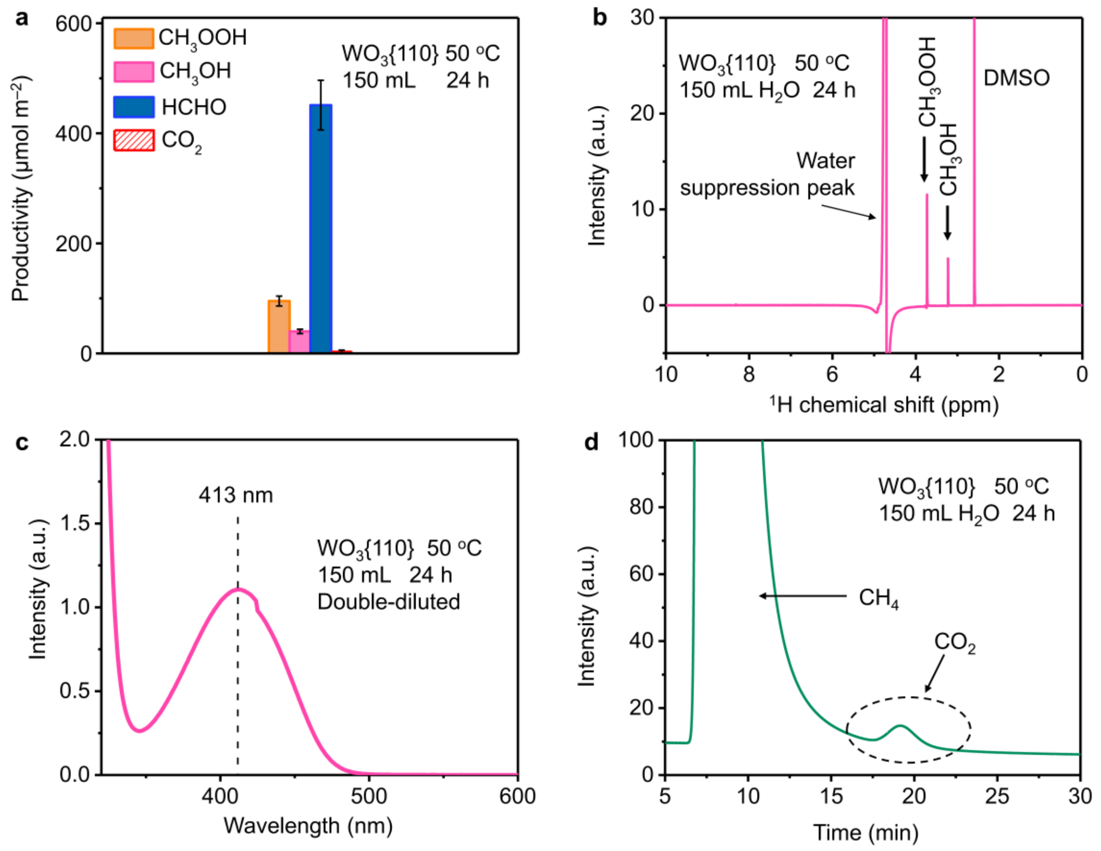

Supplementary Fig. 45 (a) Photocatalytic CH_4 oxidation performance on WO_3 {110} for 24 h reaction. (b) ^{1}H NMR spectrum of product. (c) The corresponding UV-visible absorption spectrum of HCHO product and (d) GC spectrum of gas product. Reaction condition: 10 mg catalyst, 150 mL H_2O, 9 bar O_2 + 11 bar CH_4, 24 h reaction time, Xenon light 150 mW cm^{-2}, 50 °C. Error bars indicate standard deviations.

The performance stability of WO_3 {110} is evaluated by the cyclic test and long-term reaction. After five photocatalytic cycles, the initial activity of WO_3 {110} is almost fully preserved (Supplementary Fig. 42–44). In addition, by extending the reaction time to 24 h, the productivity of oxygenates is increased by ~7.8 times (CH_3OOH, 95.21 μmol m^{-2}; CH_3OH, 40.02 μmol m^{-2}; HCHO, 451.20 μmol m^{-2}; CO_2, 2.3 μmol m^{-2}, Supplementary Fig. 45a–45d) compared with the reaction for 3 h ((CH_3OOH, 11.91 μmol m^{-2}; CH_3OH, 4.99 μmol m^{-2}; HCHO, 58.19 μmol m^{-2}; CO_2, 0 μmol m^{-2}, Fig. 3i). Both cyclic test and long-term reaction reveal the satisfied performance stability of WO_3 {110}.

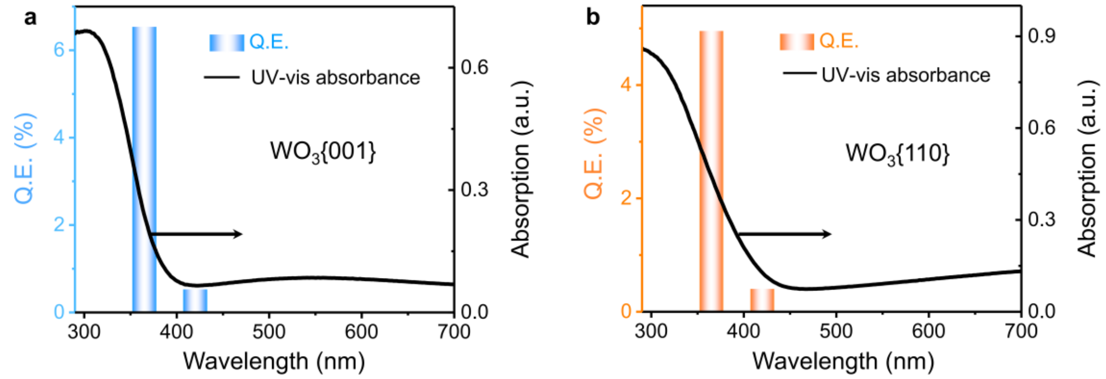

Supplementary Fig. 46 Q.E. values with different wavelength of monochromatic light irradiation at 25 °C reaction temperature, 365 nm 6.26 mW cm^{-2}, 420 nm 22.50 mW cm^{-2}, 470 nm 24.56 mW cm^{-2}, 535 nm 13.81 mW cm^{-2} and 630 nm 13.82 mW cm^{-2}, along with the diffuse reflectance spectra of (a) WO_3{001} and (b) WO_3{110}.

Q.E. test

For WO_3{001} system, 200 mg of photocatalyst was added into 150 mL of H_2O, meanwhile 11 bar O_2 + 20 bar CH_4 were injected into high pressure reactor. Under monochromatic light of 365, 420, 470, 535 and 630 nm irradiation for 3 h at 25 °C, the productivities of HCHO (Supplementary Fig. 47a and Supplementary Table 1) and CO_2 (Supplementary Fig. 47b–47f and Supplementary Table 1) were tested. Then, Q.E. values are calculated according to the following equation.:

$$\text{Q.E.} = \frac{R(electron)\ N_A}{ISt/E\lambda} \times 100\% \tag{S11}$$

where I, S, t and N_A stand for the light intensity irradiated on the sample, irradiation area (12.56 cm^2), reaction time (3 h) and Avogadro's constant (6.02 × 10^{23} mol^{-1}), respectively. The value of E_λ is calculated through the formula of $E_\lambda = h\ c\ /\ \lambda$. Taking λ = 365 nm as an example, the value of E_λ is calculated to be 5.4 × 10^{-19} J. R(electron) represents the number of electrons involved in the product formation.

WO_3{001}/ WO_3{110}

$$CH_4 + O_2 - 4\ e^- \rightarrow HCHO + H_2O \tag{S12}$$

$$CH_4 + 2O_2 - 8\ e^- \rightarrow CO_2 + 2H_2O \tag{S13}$$

As for WO_3{001}, the production of HCHO and CO_2 from CH_4 and O_2 involves 4 and 8 electrons. Taking 365 nm light irradiation as an example (I = 6.26 mW cm^{-2}), the yields of HCHO and CO_2 are 38.70 and 2.00 μmol. Then, Q.E. = 6.02 × 10^{23} mol^{-1} × (38.70 μmol × 4 + 2.00 μmol × 8) × 100% / (6.26 mW/cm^2 × 12.56 cm^2 × 3 × 3600 s /

5.4×10^{-19} J) = 6.54%. Likewise, the obtained Q.E. values at the wavelength of 420, 470, 535 and 630 nm are 0.53%, 0%, 0% and 0% (Supplementary Fig. 46a and Supplementary Table 1), respectively.

Likewise, the Q.E. values of WO_3 {110} at the wavelength of 365, 420, 470, 535 and 630 nm are 4.96%, 0.41%, 0% and 0% (Supplementary Fig. 46b, 48a–48f and Supplementary Table 1), which were also obtained with the similar reaction conditions except 14 bar O_2 + 17 bar CH_4.

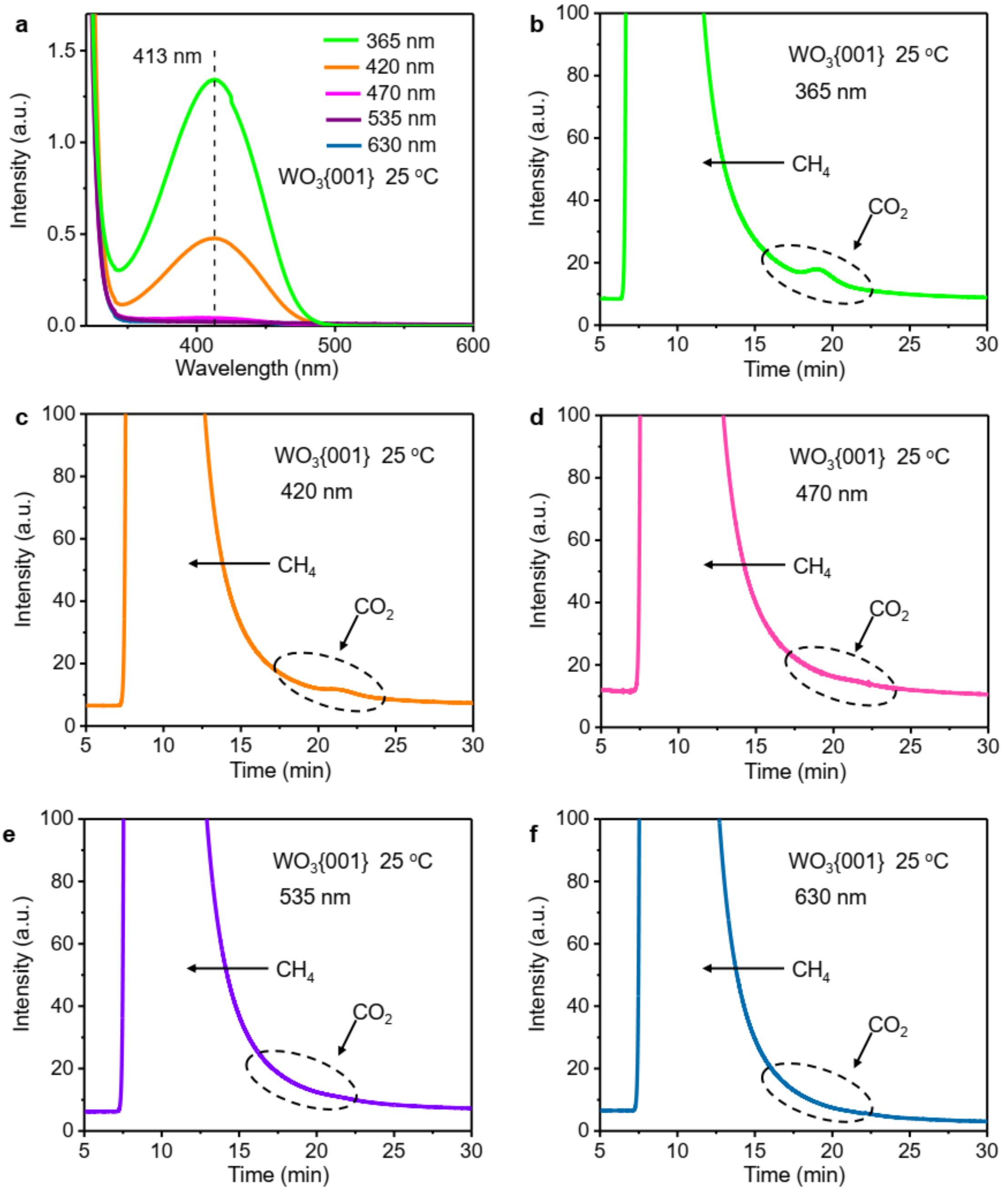

Supplementary Fig. 47 (a) UV-visible absorption spectra of HCHO product on WO_3\{001\} with different wavelength of monochromatic light irradiation. (b-f) GC spectra of gas product from CH_4 oxidation on WO_3\{001\} with different wavelength of monochromatic light irradiation. Peaks at 9.21 and 19.11 min are attributed to residual CH_4 and produced CO_2, respectively. Reaction condition: 200 mg catalyst, 11 bar O_2, 20 bar CH_4, 3 h reaction time, reaction temperature 25 °C.

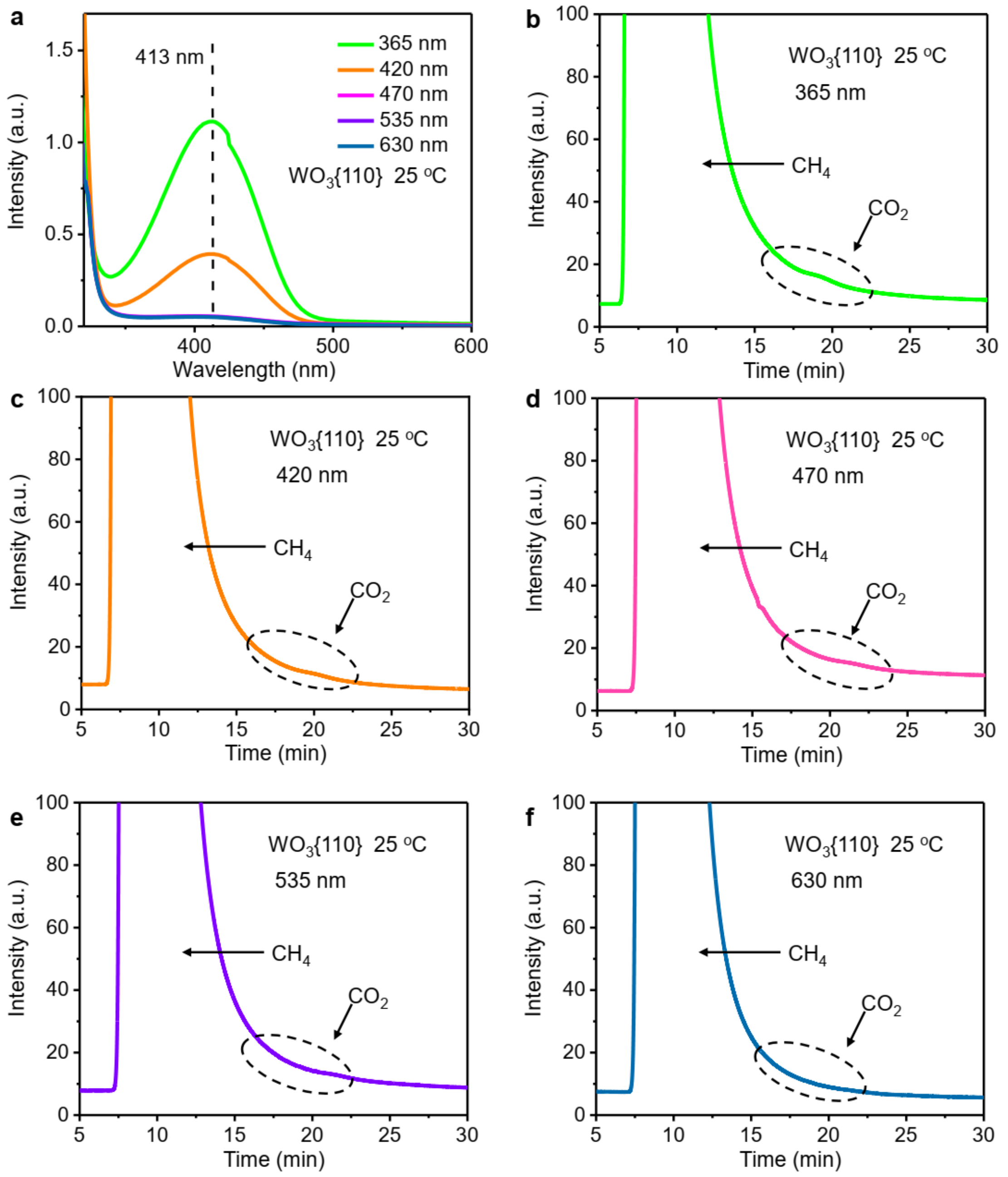

Supplementary Fig. 48 (a) UV-visible absorption spectra of HCHO product on WO_3 {110} with different wavelength of monochromatic light irradiation. (b-f) GC spectra of gas product from CH_4 oxidation on WO_3 {110} with different wavelength of monochromatic light irradiation. Peaks at 9.21 and 19.11 min are attributed to residual CH_4 and produced CO_2, respectively. Reaction condition: 200 mg catalyst, 14 bar O_2, 17 bar CH_4, 3 h reaction time, reaction temperature 25 °C.

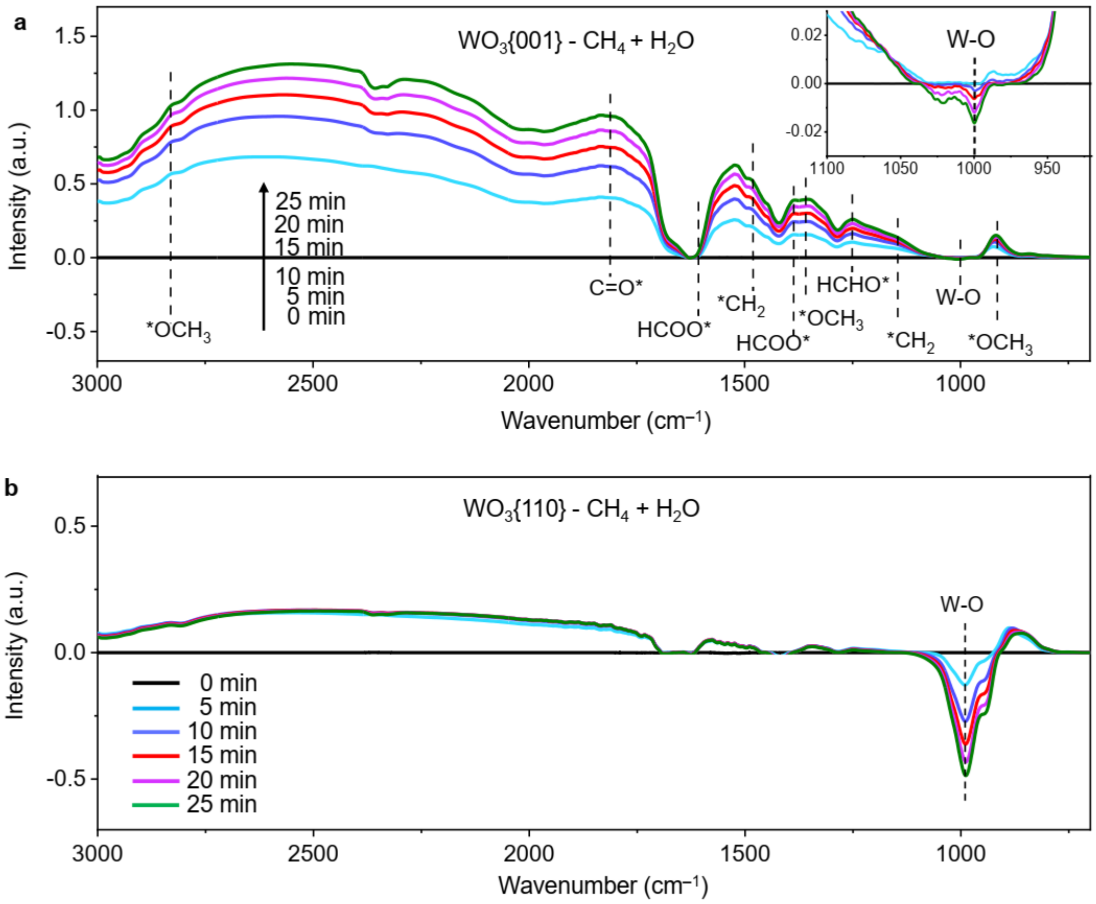

Supplementary Fig. 49 (a) *In situ* DRIFTS spectra of WO_3 {001} and (b) WO_3 {110} in pure CH_4 atmosphere with H_2O addition under different light irradiation time. Here, * denotes an adsorption site on surface. The inset is the magnified W-O peak.

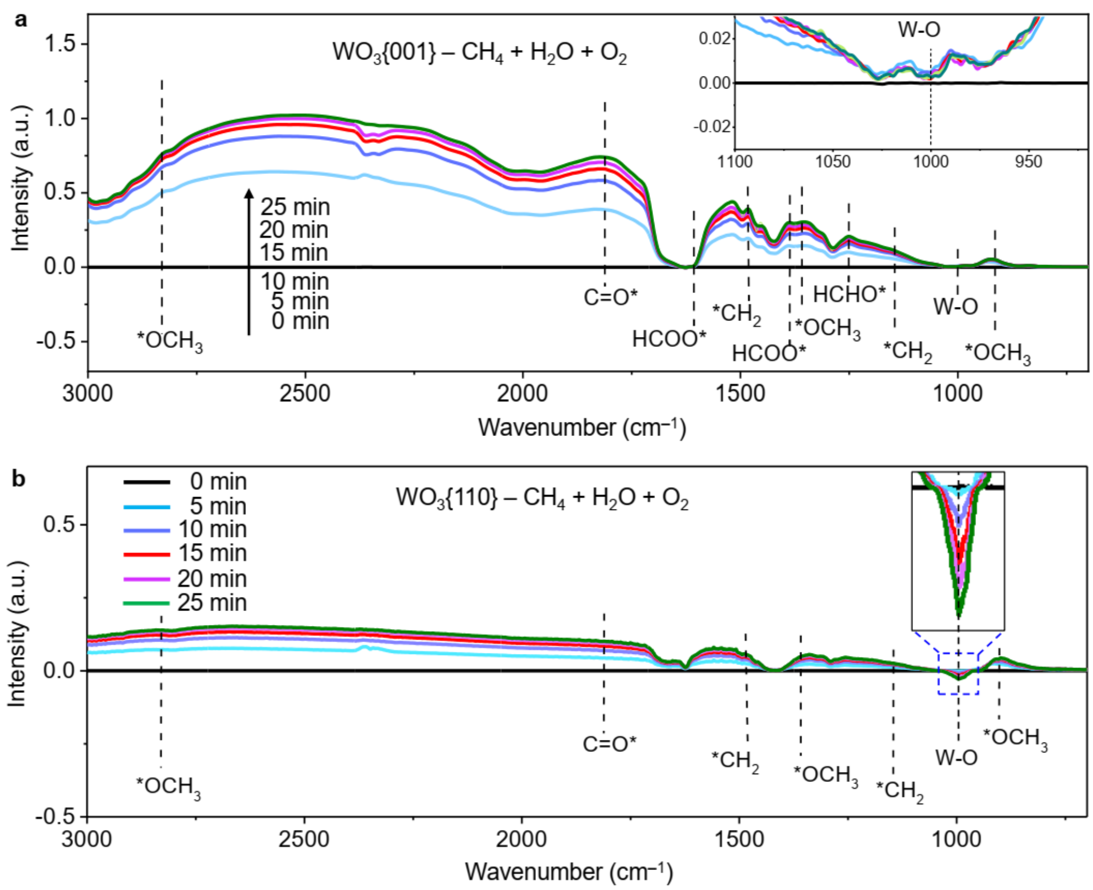

Supplementary Fig. 50 (a) *In situ* DRIFTS spectra of WO_3 {001} and (b) WO_3 {110} in the mixed $CH_4 + O_2$ atmosphere with H_2O addition under different light irradiation time. Here, * denotes an adsorption site on surface. The inset is the magnified W-O peak.

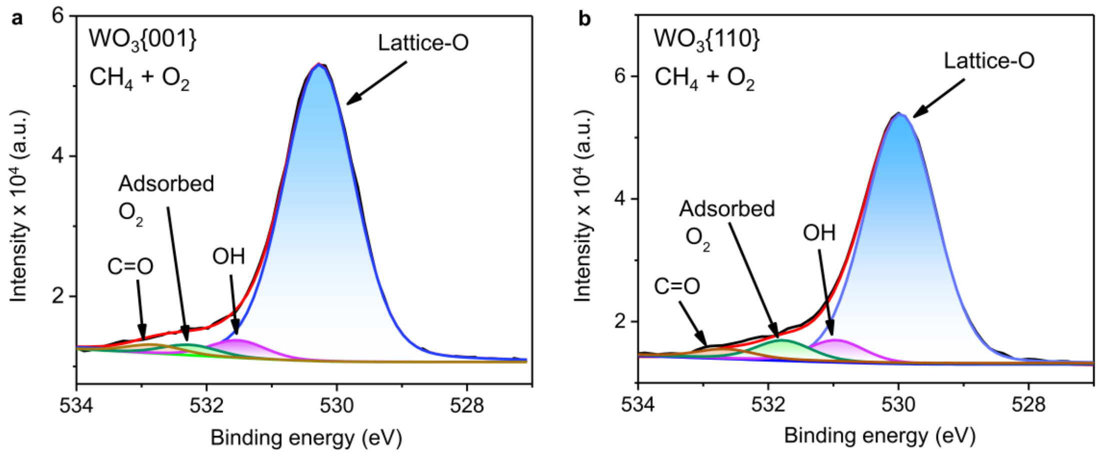

Supplementary Fig. 51 High-resolution O1*s* XPS spectra of (a) WO_3{001} and (b) WO_3{110} after reactions in the mixed CH_4 + O_2 atmosphere.

The signal of the lattice-O is elevated again, proving that the lattice-O is repaired by O_2.

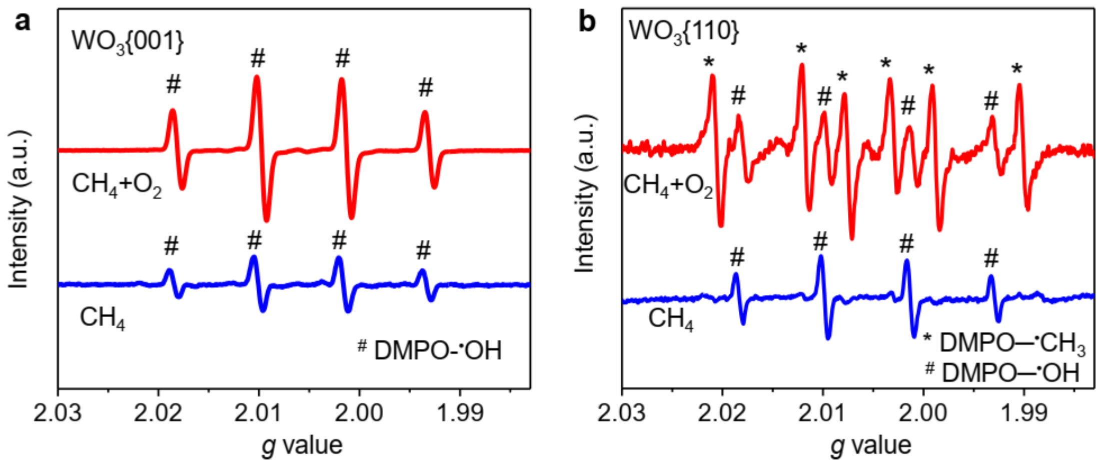

Supplementary Fig. 52 EPR spectra of (a) WO_3 {001} and (b) WO_3 {110} in pure CH_4 or CH_4 + O_2 atmosphere at room temperature with DMPO as the radical trapping agent in aqueous solution.

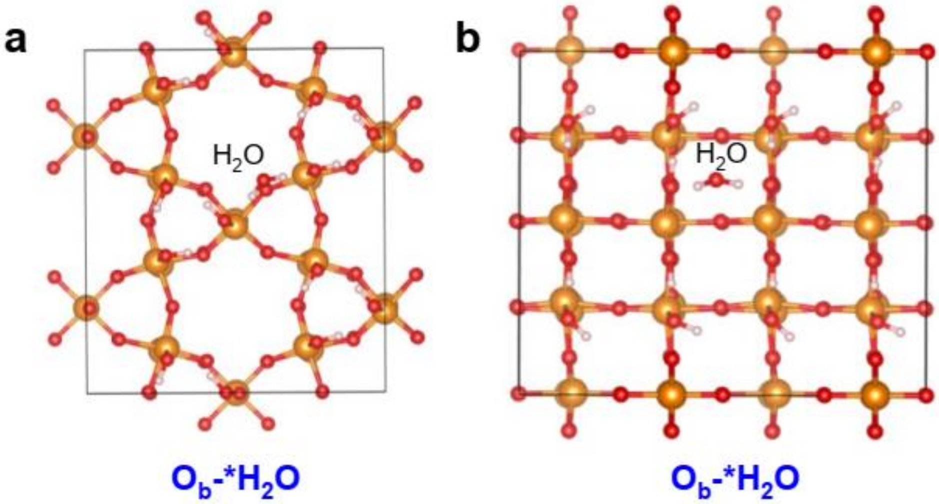

Supplementary Fig. 53 Atomic configurations of H_2O adsorption at the O_b sites on (a) WO_3\{001\} and (b) WO_3\{110\} surface.

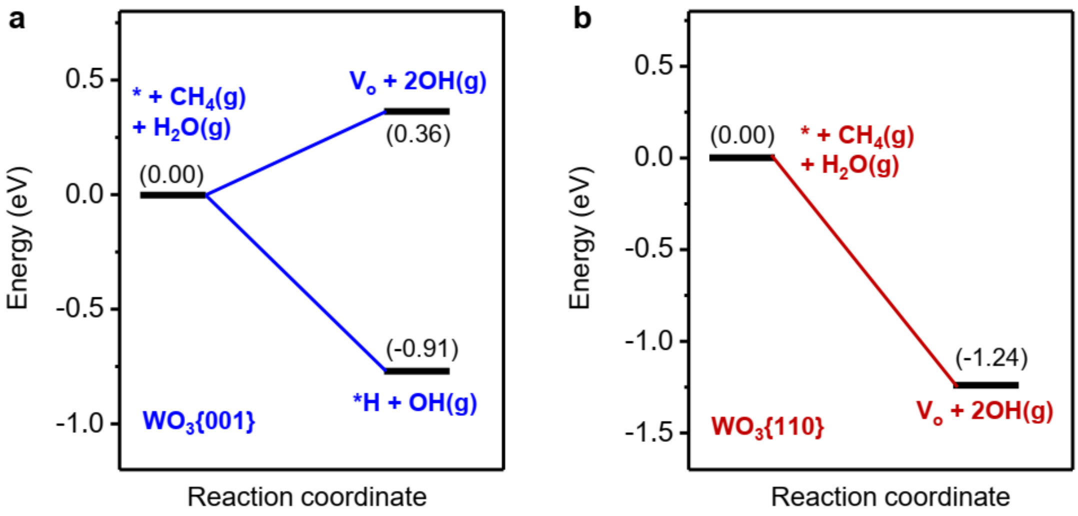

Supplementary Fig. 54 Energy diagrams of H_2O oxidation on the surface of (a) WO_3\{001\} and (b) WO_3\{110\}. The energy value of V_o + 2OH(g) on WO_3\{001\} is corrected with $-eU_g = -2.78$ eV. The energy value of V_o + 2OH(g) on WO_3\{110\} is corrected with $-eU_g = -2.52$ eV.

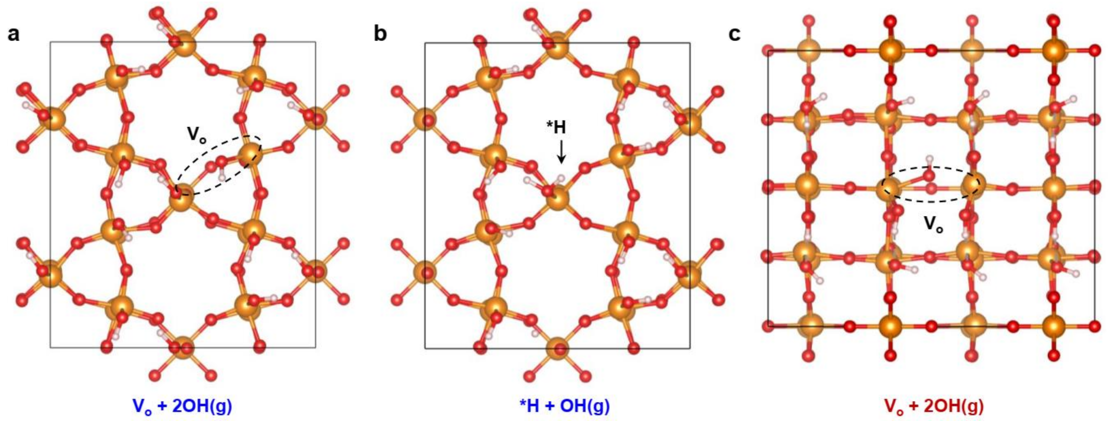

Supplementary Fig. 55 Atomic configurations of (a) V_o + 2OH(g) and (b) *H + OH(g) at O_b site on the surface of WO_3{001}, and (c) V_o + 2OH(g) at O_b site on the surface of WO_3{110}.

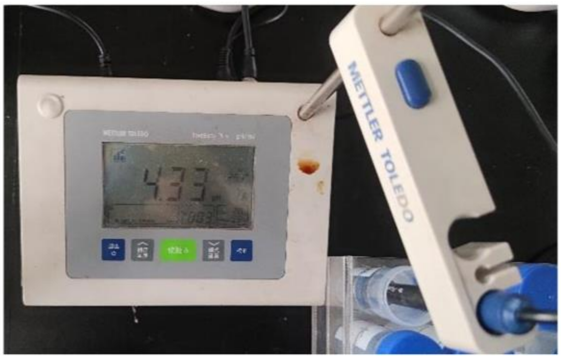

Supplementary Fig. 56 The pH value of solution after CH_4 oxidation reaction in O_2 atmosphere with WO_3{110} as photocatalyst.

In WO_3{110} system, under O_2 condition, a distinct $^{\bullet}OOH$ signal with sixfold peaks is detected (Fig. 7b). To confirm that the $^{\bullet}OOH$ radical in our reaction system originates from the protonation of $^{\bullet}O_2^{-}$ rather than the decomposition of H_2O_2, we test the pH value of solution after CH_4 oxidation reaction in O_2 atmosphere with WO_3{110} as photocatalyst. The pH value of solution is tested to be 4.33 (Supplementary Fig. 56). According to the previous reports[49], the $^{\bullet}OOH$ radical generated in acid solution (pH < 4.8) results from the protonation of $^{\bullet}O_2^{-}$ radical. The $^{\bullet}O_2^{-}$ radical is the dissolved state of O_2^{-} ion in neutral (or alkaline) solution, which is the single electron reduction product of O_2 on the photocatalyst surface. The occurrence of single electron reduction of O_2 is attributed to its triplet state ($^3\Sigma_g^{-}$), which possesses two unpaired electrons on each of the two antibonding π orbitals (π_x* and π_y*) at the same energy level[42]. This state of O_2 ensures its reactivity as a strong electron scavenger when adsorbed on the catalyst surface, forming the O_2^{-} anion. Noteworthily, the decomposition of H_2O_2 to produce ^{-}OOH (the alkaline form of $^{\bullet}OOH$ radical) only occurs in alkaline solutions (pK_a = 11.7)[42]. Furthermore, we cannot assume that the ^{-}OOH anion plays the same role in CH_4 oxidation as the $^{\bullet}OOH$ radical since no relevant work has been reported. Thus, we suggest that single electron reduction of O_2 on the WO_3{110} surfaces produce O_2^{-} anions, then giving rise to $^{\bullet}OOH$ radicals in acid solution.

.

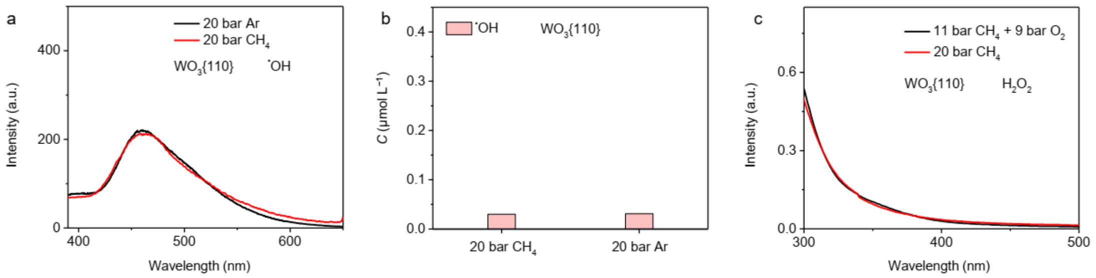

Supplementary Fig. 57 (a) Fluorescence spectra of 1 mM coumarin solution after reaction over WO_3\{110\}. (b) Calculated concentration of •OH radicals. (c) H_2O_2 monitoring by real-time UV-vis absorption spectra through color-developing method. Reaction condition: 10 mg WO_3\{110\}, 20 bar CH_4 or 20 bar Ar, 5 mL H_2O, 50 °C reaction temperature, 3 h reaction time, Xenon light 150 mW cm^{-2}.

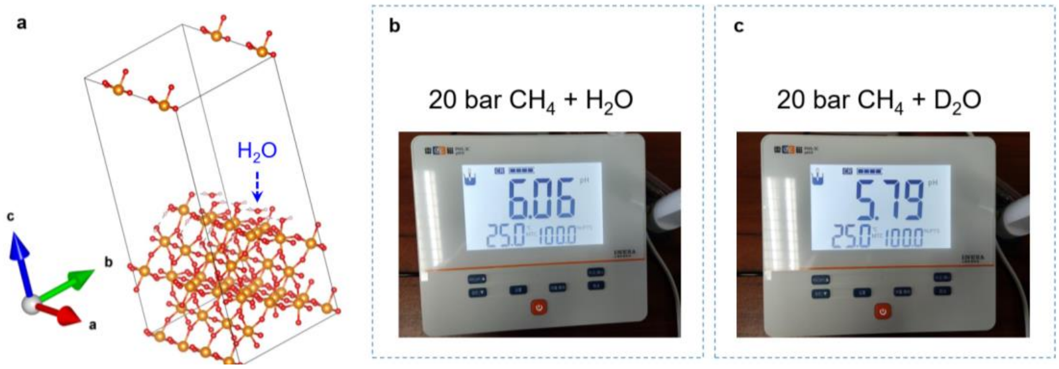

Supplementary Fig. 58 (a) Spontaneous dissociation or combination of terminal OH group or terminal H atom on WO_3\{110\} surface. (b) The pH value of solution after reaction in H_2O or (c) D_2O solution with 20 bar CH_4 addition for 3 h at 50 °C.

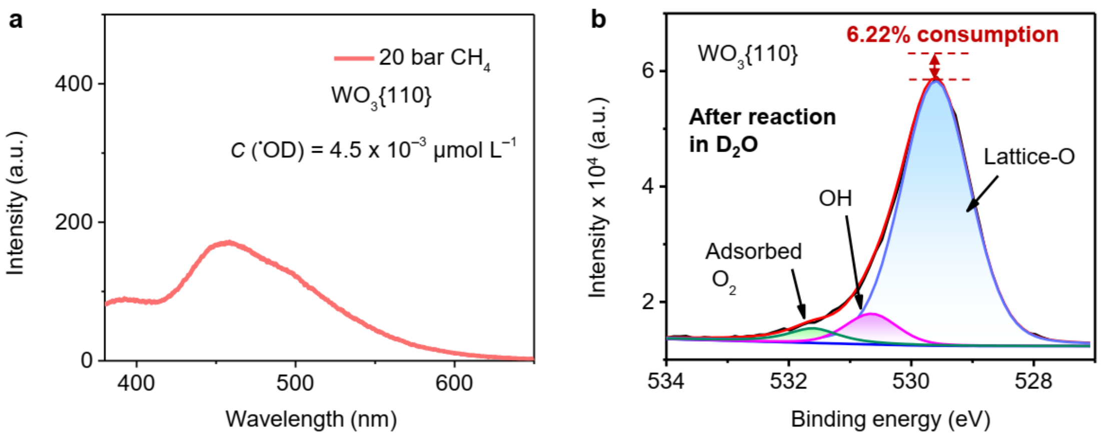

Supplementary Fig. 59 (a) Fluorescence spectrum of 1 mM coumarin solution taking WO_3\{110\} as photocatalyst with 20 bar CH_4 and 5 mL D_2O as solvent. (b) High-resolution O1*s* XPS spectra of WO_3\{110\} after reaction in CH_4 atmosphere with 5 mL D_2O as solvent.

The $^{\bullet}OH$ radical generated in WO_3\{110\} system is mainly quenched though it can be clearly detected.

As shown by the *in situ* DRIFT/XPS results, a higher lattice-O loss is observed on WO_3\{110\} than WO_3\{001\} in pure CH_4 atmosphere. This is because in aqueous solutions, H_2O molecules are easily adsorbed on lattice-O of WO_3\{110\} and oxidized to produce $^{\bullet}OH$ radicals ($O_b + h^+ + H_2O \rightarrow V_o + 2^{\bullet}OH$) with lattice-O participation, which is verified by the DFT calculations (Supplementary Fig. 54b and 55c). During this process, the lattice-O of WO_3\{110\} is largely consumed and oxygen vacancy is formed. Therefore, the lattice-O consumption of WO_3\{110\} should be equal to half of the $^{\bullet}OH$ radical production without O_2 addition (consumed lattice-O of WO_3\{110\} = $0.5 \times C$ ($^{\bullet}OH$)). In WO_3\{001\} system, since the lattice-O participates into HCHO formation, the amount of consumed lattice-O is equal to that of HCHO produced without O_2 addition (consumed lattice-O of WO_3\{001\} = C (HCHO)). Based on the XPS results, after the reaction in CH_4 atmosphere, the amount of lattice-O consumed on WO_3\{110\} should be 1.5 times than that of WO_3\{001\}, that is $0.5 \times C$ ($^{\bullet}OH$) = $1.5 \times C$ (HCHO) $\rightarrow$ C ($^{\bullet}OH$) = $3 \times C$ (HCHO). However, through coumarin fluorescence test, the $^{\bullet}OH$ radical generated over WO_3\{110\} after reactions in CH_4 atmosphere (or Ar atmosphere) is quantified to be only ~0.03 μmol L^{-1} for 3 h reaction time in 5 mL H_2O (Supplementary Fig. 57a and 57b), which is far less than the generated HCHO (17.8 μmol L^{-1}, that is 0.567 μmol m^{-2}, Supplementary Fig. 11) over WO_3\{001\} under the same reaction condition. Moreover, no H_2O_2 is generated (Supplementary Fig. 57c). Thus, we hypothesize that the $^{\bullet}OH$ must be quenched soon after its formation. Seen from the DFT calculation on WO_3\{110\}, the terminal OH group or the H atom is always spontaneously dissociated from the surface and likely combined to form H_2O molecule due to the high surface energy (Supplementary Fig. 58a). And the number of dissociated terminal H atoms is greater than that of dissociated terminal OH groups, which is revealed by the acidic nature of solution (pH = 6.06) after reaction in 20 bar CH_4 atmosphere with H_2O as solvent (Supplementary Fig. 58b). Given this, we deduce that the $^{\bullet}OH$ radical generated by lattice-O is involved in this process and then quenched. As a result, the detected number of $^{\bullet}OH$ radical is far less than the consumed amount of lattice-O over WO_3\{110\}. To verify that lattice-O consumption is indeed caused by H_2O oxidation, we use D_2O aqueous solution containing coumarin to carry out free radical trapping experiments. After light irradiation for 3 h in 20 bar CH_4, weak

fluorescent signal is observed indicating that trace amount of $^{\bullet}OD$ is generated (4.5×10^{-3} μmol L^{-1}, Supplementary Fig. 59a). This value is much lower than the generated $^{\bullet}OH$ radical amount (0.03 μmol L^{-1}) with H_2O as solvent, which is attributed to the low kinetic energy of deuterium isotope effect. The pH value of the reaction solution is tested to be 5.79 (Supplementary Fig. 58c), which is lower than that of H_2O solution (pH = 6.06). The lower pH value is reasonable considering that the consumption of H^+ is less with the smaller amount of $^{\bullet}OD$ radical produced in D_2O. The O1*s* XPS spectra of the recycled WO_3\{110\} further show that only 6.22% of the lattice-O is consumed using D_2O as solvent (Supplementary Fig. 59b). Altogether, we suppose that the lattice-O of WO_3\{110\} is indeed participated and consumed in H_2O oxidation to form $^{\bullet}OH$ radical, which is easily quenched by combing with the dissociated H atom from the surface OH group of WO_3\{110\} instead of involvement of C_1 oxygenates formation. Moreover, due to the higher surface energy of WO_3\{110\} facets (4.13 J m^{-2}) than WO_3\{001\} facets (2.46 J m^{-2})[3, 50], the detachment of lattice-O on WO_3\{110\} is accelerated. Thus, the WO_3\{110\} is more likely to lose lattice-O as revealed from the *in situ* DRIFT and XPS results.

The •OH radical generated in WO_3\{110\} system is not involved in oxygenates production.

The •OH radical generated in WO_3\{110\} system is quantified with O_2 addition. Both in 9 bar O_2 + 11 bar Ar and 9 bar O_2 + 11 bar CH_4 atmosphere under light irradiation for 3 h at 50 °C, the generated •OH radical amount is ~0.33 μmol L^{-1} (Supplementary Fig. 60a and 60b). According to previous reports[38, 42], the •OH radical is generated through two electron reduction process of O_2 using H_2O_2 as intermediate ($O_2 + 2H^+ + 2e^- = H_2O_2 = 2{}^\bullet OH$, E ($O_2$, $2H^+/H_2O_2$) = 0.695 V *vs* NHE). Yet no signal of H_2O_2 is detected, indicating that all the H_2O_2 is decomposed (Supplementary Fig. 60c). Compared to the •OH radical (0.03 μmol L^{-1}) in absence of O_2, the concentration of •OH radical upon O_2 addition (0.33 μmol L^{-1}) is increased 11-fold. This result manifests that the •OH radical upon O_2 addition is mainly from O_2 reduction rather H_2O decomposition. Although the amount of •OH radical is increased after O_2 addition, compared to the generated CH_3OH concentration (12.59 μmol L^{-1}, Fig. 3e), its concentration (0.33 μmol L^{-1}) is still very low, suggesting that CH_3OH mainly originates from CH_3OOH decomposition rather than the combination between •CH_3 radical and •OH radical. Besides, taking coumarin as the trapping agent for •OH radical, the type and yield of oxygenates are not changed, indicating that •OH radical is not involved in CH_4 oxidation process (Supplementary Fig. 61). Isotopically labeled D_2O and $H_2{}^{18}O$ as solvents are added to track whether H_2O is involved in oxygenates formation and the role of oxygen vacancy. Taking WO_3\{110\} as photocatalyst in 5 mL D_2O and 11 bar CH_4 + 9 bar O_2 atmosphere (Supplementary Fig. 62 and 63a, 63c, 63e), the yield of oxygenates is reduced in compared with that using H_2O as solvent (Fig. 3e). This is because, using WO_3\{110\} as catalyst to oxidize CH_4 with O_2, the first lattice-O is needed to decompose H_2O to produce oxygen vacancy, and then oxygen vacancy adsorbs O_2 to participate in CH_4 oxidation. Using D_2O as a solvent, the decomposition process of D_2O by lattice-O is difficult, and thus the number of oxygen vacancies and adsorbed O_2 is reduced (Supplementary Fig. 59b). Notably, taking the recycled WO_3\{110\} after reaction in 20 bar CH_4 and 5 mL H_2O as photocatalyst and being re-fed into 5 mL D_2O and 11 bar CH_4 + 9 bar O_2 atmosphere, the yield of oxygenates is found to be similar to that using H_2O as solvent (Supplementary Fig. 62 and 63b, 63d, 63f). This is because the recycled WO_3\{110\} is already equipped with sufficient oxygen vacancy, ensuring the O_2 adsorption and CH_4 oxidation (Fig. 4d). After reaction,

the HCHO signal generated in D_2O solvent demonstrates that the D atom of D_2O cannot participate in CH_4 oxidation into HCHO (Supplementary Fig. 64a and 65a), and the H atom of HCHO does not undergo H-D exchange with D_2O (Supplementary Fig. 64a and 65b). Despite CH_3OD signal is detected (Supplementary Fig. 64b and 66a), it may be caused by H-D exchange between CH_3OH and D_2O (Supplementary Fig. 64b and 66b).

Using $^{18}O_2$ + H_2O or O_2 + $H_2{}^{18}O$ as reactants and solvents, we find that only $CH_3{}^{18}OH$ (Supplementary Fig. 67 and 68a) and $HCH^{18}O$ (Fig. 9c and Supplementary Fig. 78a) or $CH_3{}^{16}OH$ (Supplementary Fig. 67 and Supplementary Fig. 68b) and $HCH^{16}O$ (Fig. 9c and Supplementary Fig. 78b) are generated, further validating that H_2O cannot participate into CH_3OH and HCHO formation through $^{\bullet}OH$ radical.

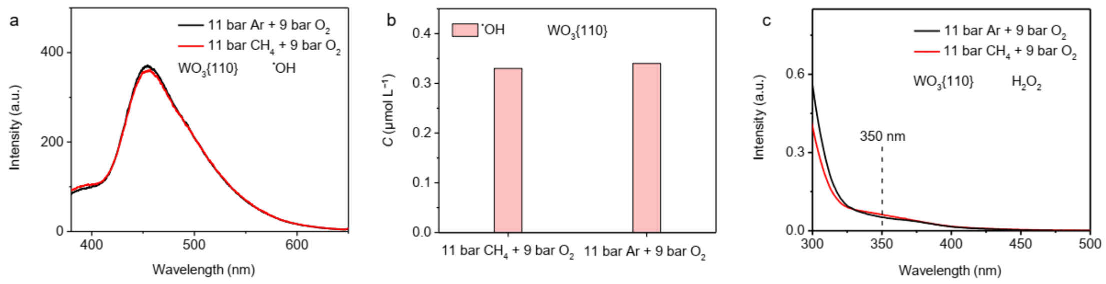

Supplementary Fig. 60 (a) Fluorescence spectra of 1 mM coumarin solution taking WO_3\{110\} as photocatalyst in 11 bar CH_4 + 9 bar O_2 or 11 bar Ar + 9 bar O_2 atmosphere. (b) Calculated concentration of $^{\bullet}OH$ radicals. (c) H_2O_2 monitoring by real-time UV-vis absorption spectra through color-developing method. Reaction condition: 10 mg WO_3\{110\}, 11 bar CH_4 + 9 bar O_2 or 11 bar Ar + 9 bar O_2, 5 mL H_2O, 50 ^{o}C reaction temperature, 3 h reaction time, Xenon light 150 mW cm^{-2}.

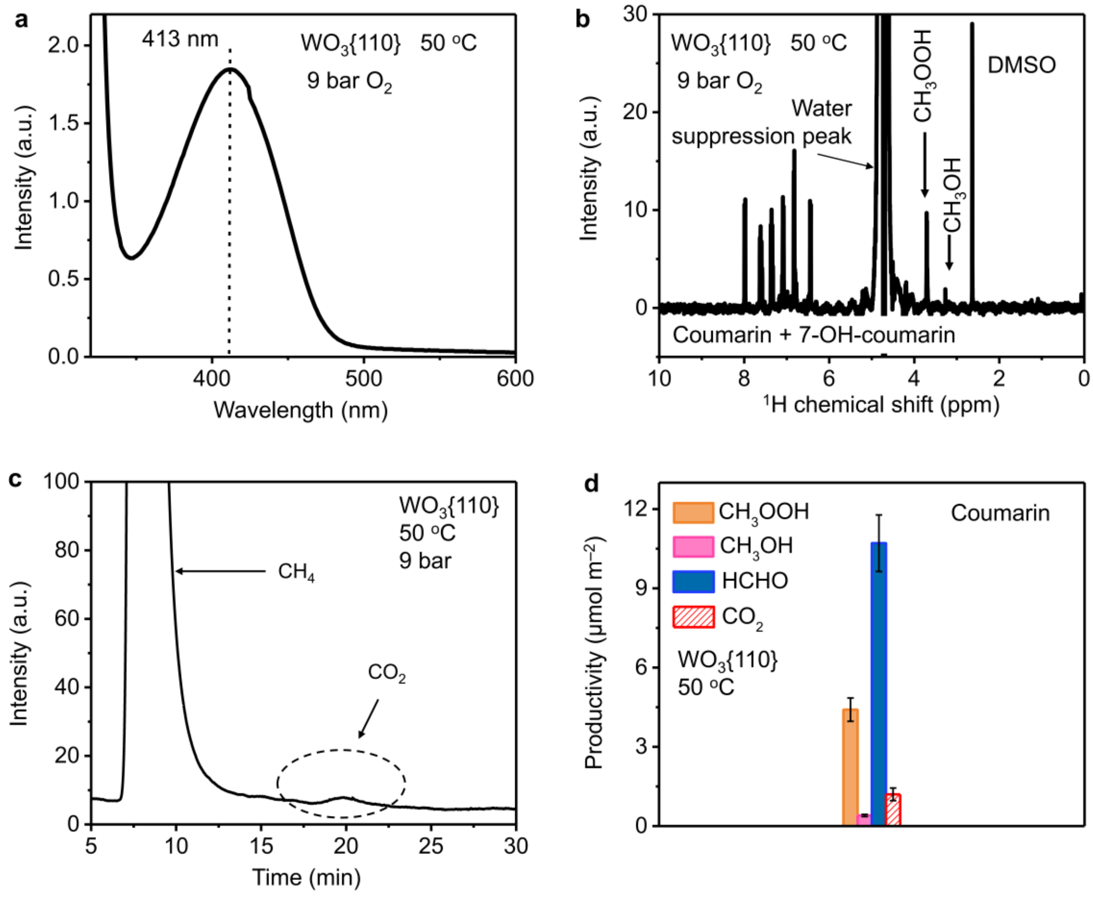

Supplementary Fig. 61 Photocatalytic CH_4 oxidation performance over WO_3\{110\} using coumarin as •OH radical trapping agent. (a) UV-visible absorption spectrum of HCHO product. (b) ^{1}H NMR spectrum of oxygenates. (c) GC spectrum of gas product. (d) Yield of oxygenates from CH_4 oxidation. Reaction condition: 10 mg WO_3\{110\}, 5 mL 1mM coumarin solution, 11 bar CH_4 + 9 bar O_2, 3 h reaction time, Xenon light 150 mW cm^{-2}. Error bars indicate standard deviations.

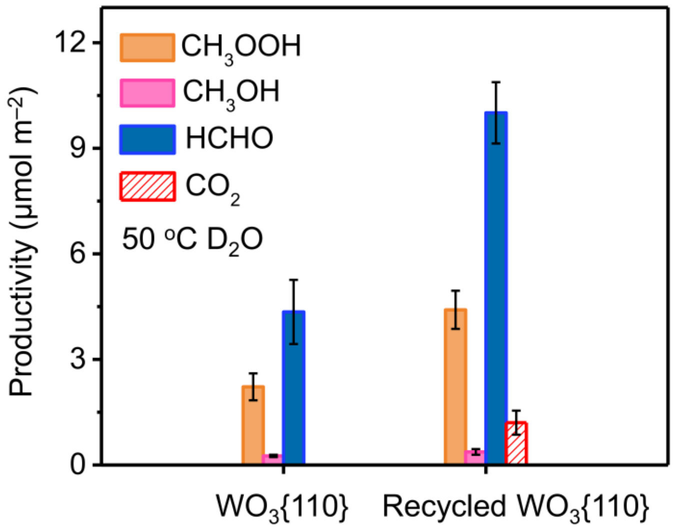

Supplementary Fig. 62 Photocatalytic CH_4 oxidation performance on WO_3 {110} and recycled WO_3 {110} with D_2O as solvent. Recycled WO_3 {110} is obtained after CH_4 oxidation without O_2 addition and with H_2O solvent. Reaction condition: 10 mg catalyst, 5 mL D_2O, 11 bar CH_4 + 9 bar O_2 = 20 bar, 3 h reaction time, Xenon light 150 mW cm^{-2}. Error bars indicate standard deviations.

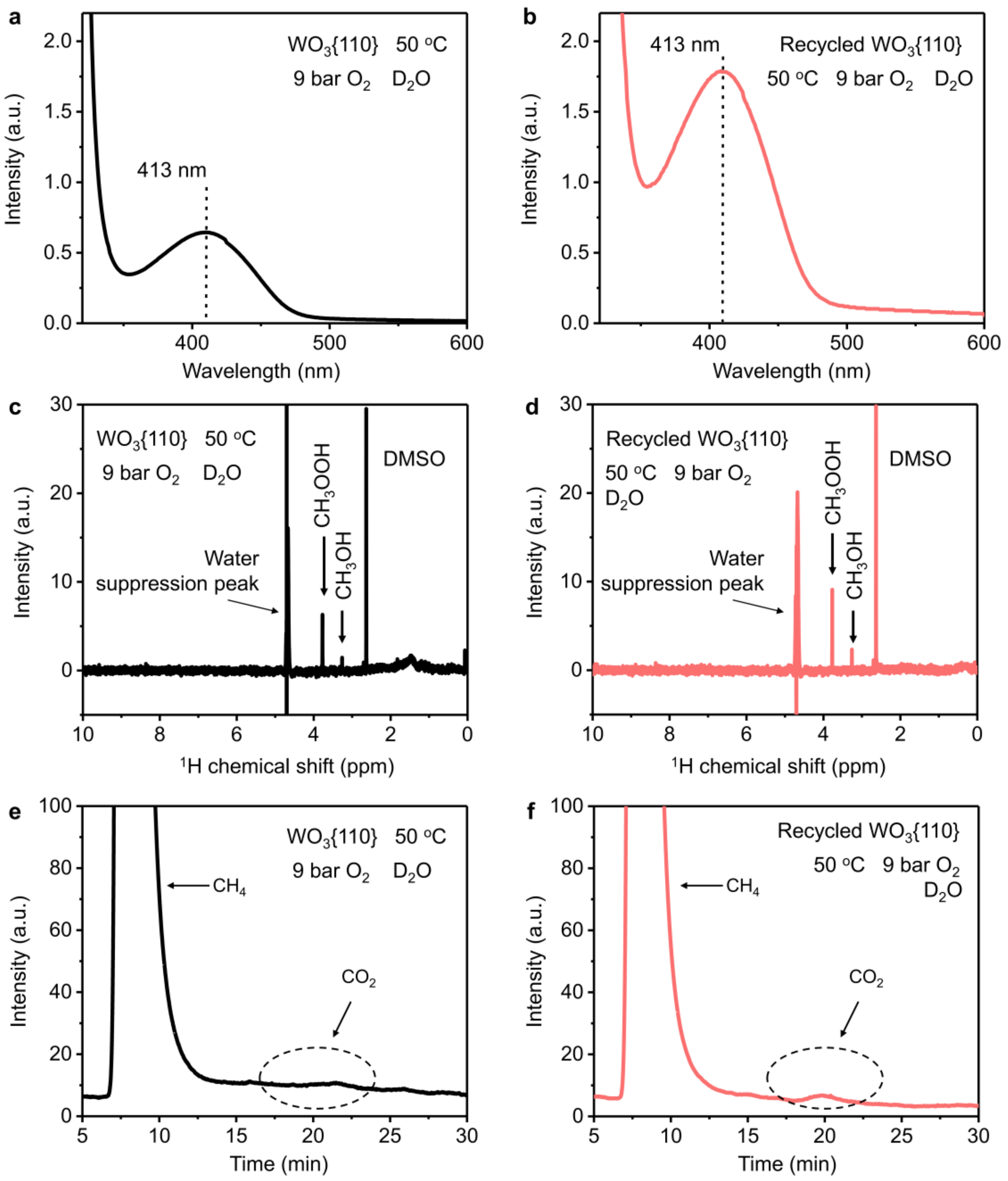

Supplementary Fig. 63 Photocatalytic CH_4 oxidation performance on WO_3 {110} and recycled WO_3 {110} with D_2O as solvent. (a) UV-visible absorption spectra of HCHO product on WO_3 {110} and (b) recycled WO_3 {110}. (c) ^{1}H NMR spectra of oxygenates on WO_3 {110} and (d) the recycled WO_3 {110}. (e) GC spectra of gas product on WO_3 {110} and (f) recycled WO_3 {110}. Recycled WO_3 {110} is obtained after CH_4 oxidation without O_2 addition and with H_2O solvent. Reaction conditions: 10 mg catalyst, 5 mL D_2O, 11 bar CH_4 + 9 bar O_2 = 20 bar, 3 h reaction time, Xenon light 150 mW cm^{-2}.

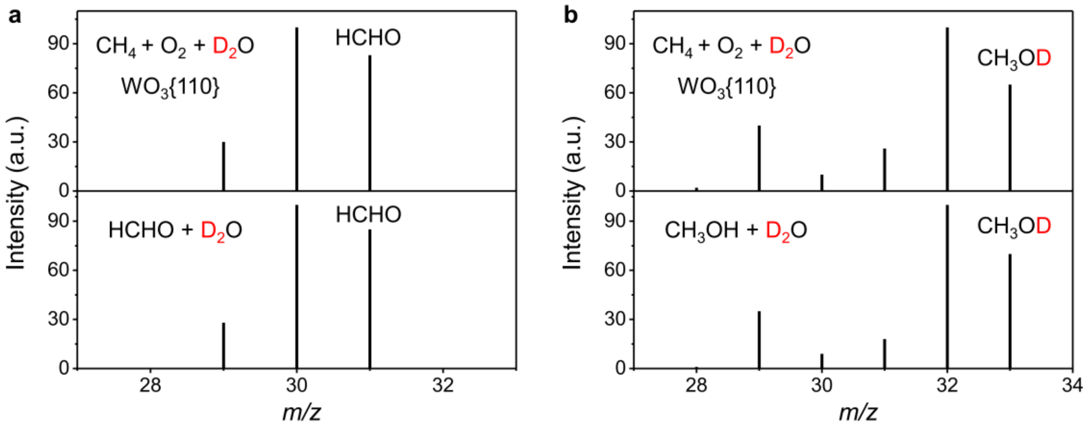

Supplementary Fig. 64 (a) GC-MS spectra of HCHO obtained from CH_4 oxidation using D_2O as solvent over WO_3\{110\}, or from 20 mM HCHO D_2O solution. The 20 mM HCHO D_2O solution is prepared by mixing 16 μL 37% HCHO solution with 10 mL D_2O under stirring for 3 h. (b) GC-MS spectra of CH_3OH obtained from CH_4 oxidation using D_2O as solvent over WO_3\{110\}, or from 20 mM CH_3OH D_2O solution. The 20 mM CH_3OH D_2O solution is prepared by mixing 11 μL 98% CH_3OH solution with 10 mL D_2O under stirring for 3 h.

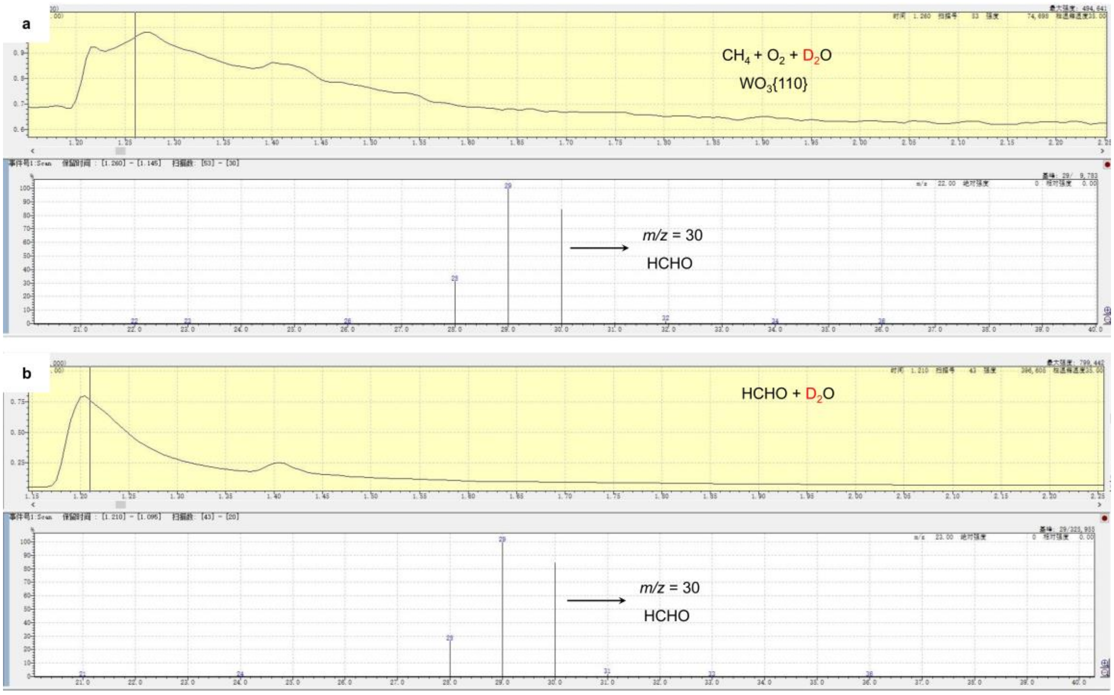

Supplementary Fig. 65 (a) Screenshot on GC-MS spectra of HCHO obtained from CH_4 oxidation using D_2O as solvent over $WO_3\{110\}$, (b) or from 20 mM HCHO D_2O solution. The 20 mM HCHO D_2O solution is prepared by mixing 16 μL 37% HCHO solution with 10 mLD_2O under stirring for 3 h.

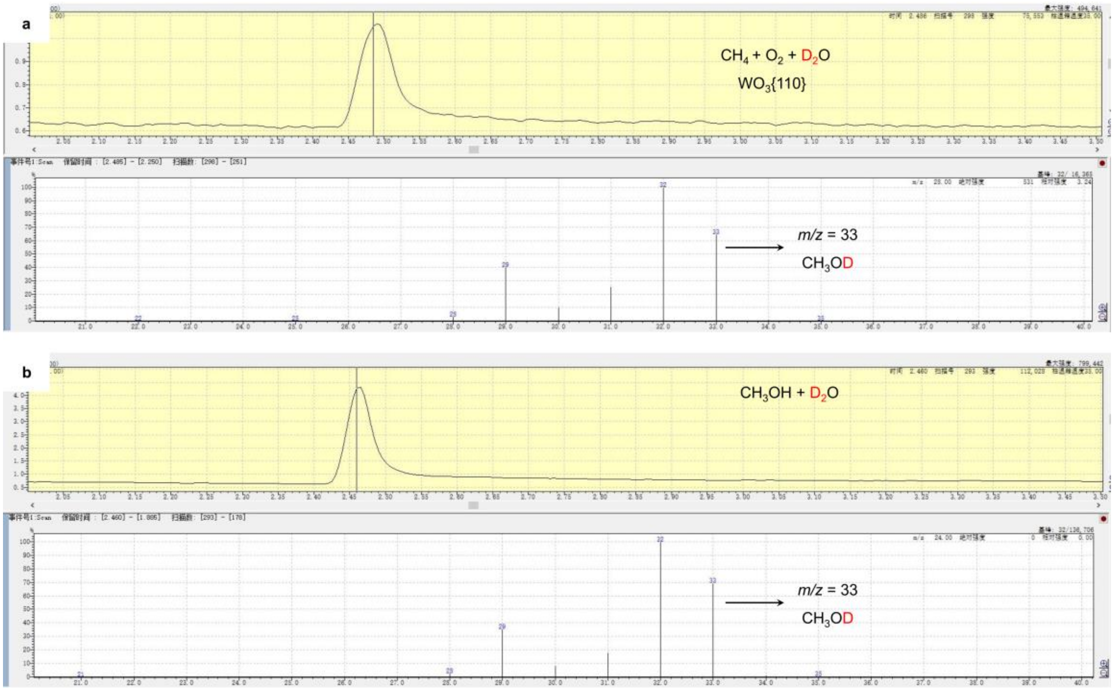

Supplementary Fig. 66 (a) Screenshot on GC-MS spectra of CH_3OH obtained from CH_4 oxidation using D_2O as solvent over $WO_3\{110\}$, (b) or from 20 mM CH_3OH D_2O solution. The 20 mM CH_3OH D_2O solution is prepared by mixing 11 μL 98% CH_3OH solution with 10 mL D_2O under stirring for 3 h.

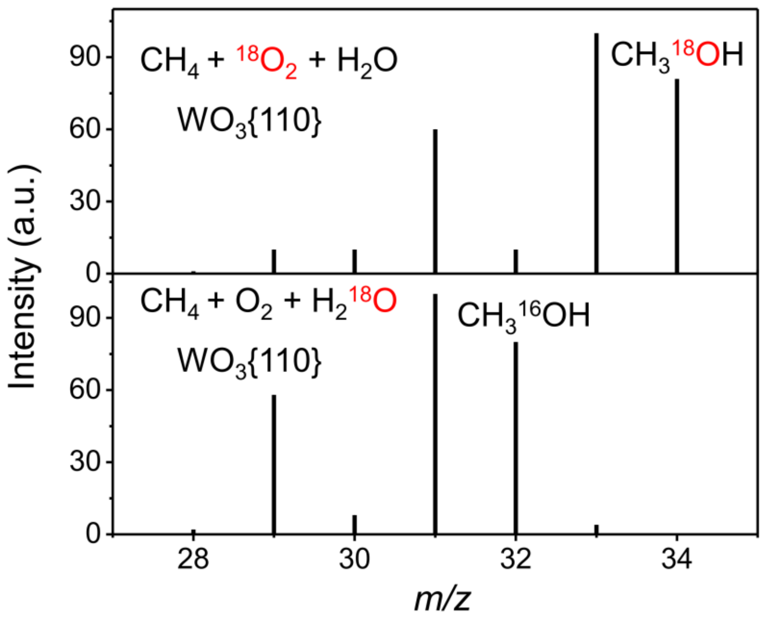

Supplementary Fig. 67 GC-MS spectra of CH_3OH obtained from CH_4 oxidation using $^{18}O_2$ or $H_2{}^{18}O$ over WO_3\{110\}. Reaction condition: 10 mg catalyst, 11 bar CH_4, 9 bar $^{18}O_2$ + 5 mL H_2O or 9 bar O_2 + 5 mL $H_2{}^{18}O$, 3 h reaction time, Xenon light 150 mW cm^{-2}, 50 ^{o}C reaction temperature.

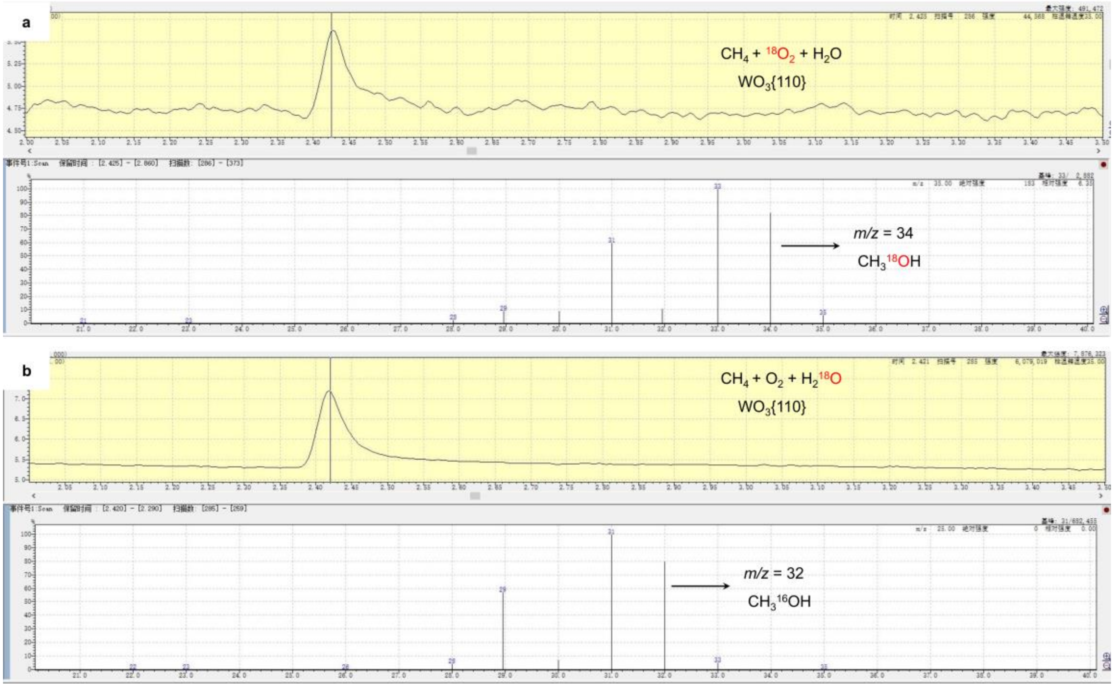

Supplementary Fig. 68 (a) Screenshot on GC-MS spectra of CH_3OH obtained from CH_4 oxidation using $^{18}O_2$ or (b) $H_2{}^{18}O$ over WO_3{110}. Reaction condition: 10 mg catalyst, 11 bar CH_4, 9 bar $^{18}O_2$ + 5 mL H_2O or 9 bar O_2 + 5 mL $H_2{}^{18}O$, 3 h reaction time, Xenon light 150 mW cm^{-2}.

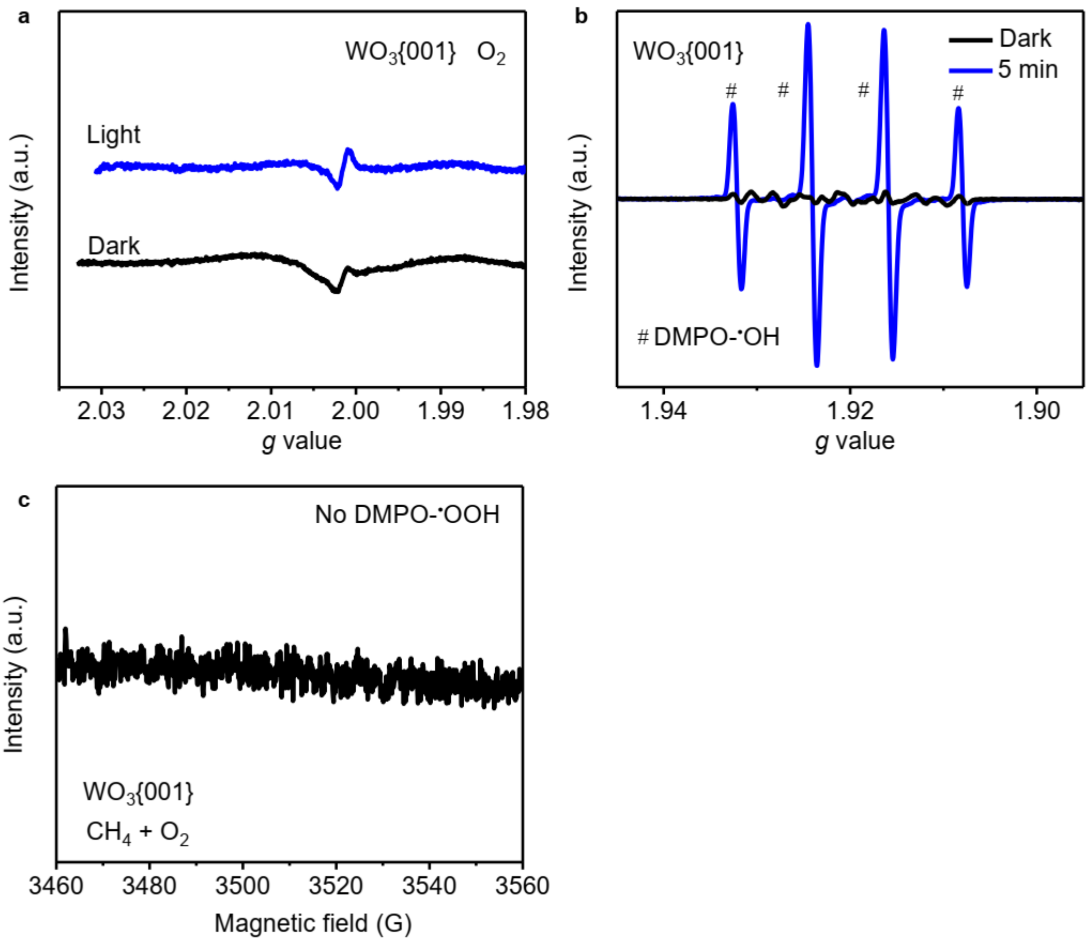

Supplementary Fig. 69 (a) EPR spectra of $WO_3\{001\}$ in O_2 atmosphere at 77 K. The $WO_3\{001\}$ is the recycled sample after CH_4 oxidation reaction without O_2. (b) EPR spectra of $WO_3\{001\}$ in the mixed O_2 + CH_4 atmosphere at room temperature with DMPO as the radical trapping agent in aqueous solution. (c) EPR spectrum of $WO_3\{001\}$ under light irradiation for 80 s with CH_4 and O_2 dissolved in methanol. DMPO is added to the reaction mixture as the radical trapping agent. The $WO_3\{001\}$ is the recycled sample after CH_4 oxidation reaction without O_2.

No signal change is observed on $WO_3\{001\}$ before and after light irradiation, indicating that no O^{2-} anion is formed on the surface of $WO_3\{001\}$ with O_2 addition (Supplementary Fig. 69a). After the incorporation of CH_4, only DMPO-$^{\bullet}$OH radical is observed without $^{\bullet}CH_3$ and $^{\bullet}$OOH radicals (Supplementary Fig. 69b and 69c). The different intermediates over $WO_3\{001\}$ and $WO_3\{110\}$ reveal the distinct HCHO formation process in the mixed CH_4 and O_2 atmosphere.

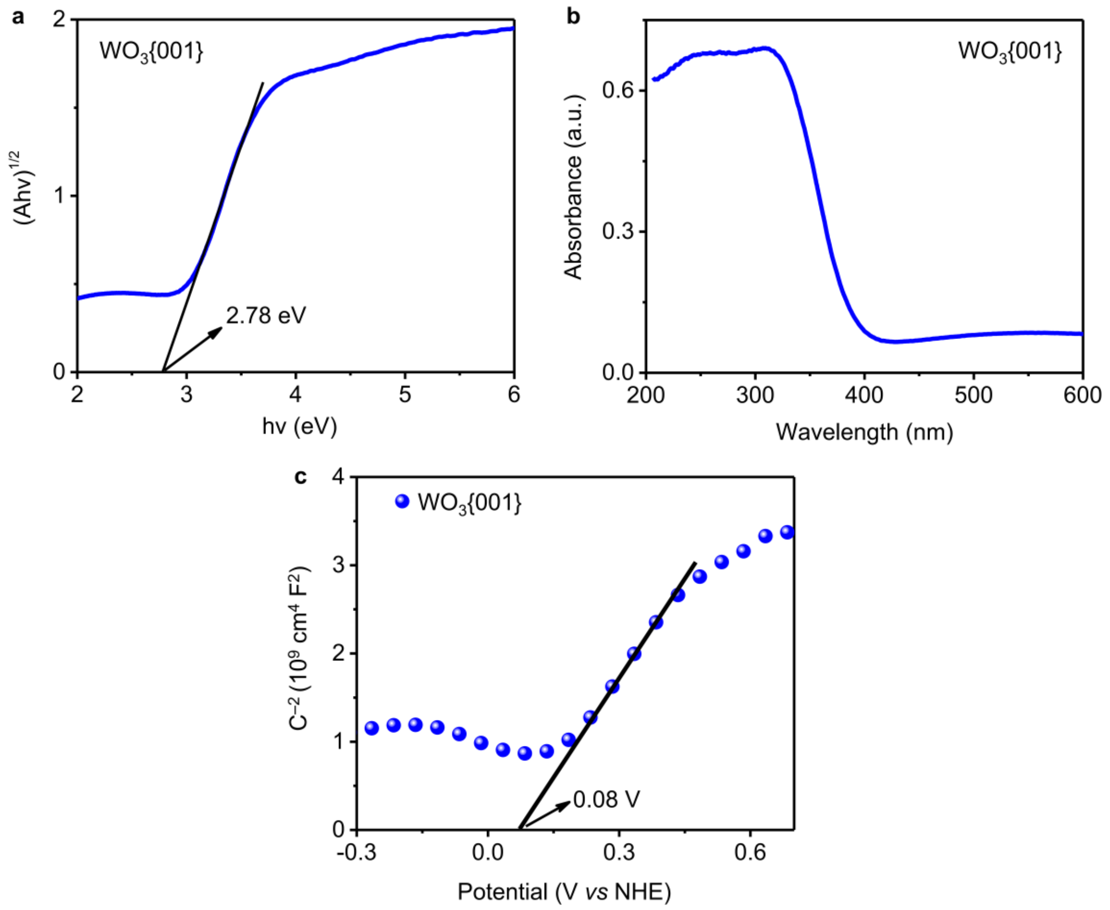

Supplementary Fig. 70 (a) Kubelka-Munk function of WO_3{001} for bandgap test. (b) UV-visible diffuse reflectance absorption plot of WO_3{001}. (c) Mott-Schottky test of WO_3{001} at the frequency of 1500 Hz.

The Kubelka-Munk plot (Supplementary Fig. 70a) transformed from UV-visible diffuse reflectance spectrum (Supplementary Fig. 70b) is used to estimate the bandgap value of WO_3{001} (2.78 eV). The conduction band of WO_3{001} is tested to be 0.08 V *vs* NHE, which is labelled from the corresponding Mott-Schottky curve (Supplementary Fig. 70c). Based on the value of bandgap and conduction band, the valence band potential of WO_3{001} is calculated to be 2.86 V *vs* NHE.

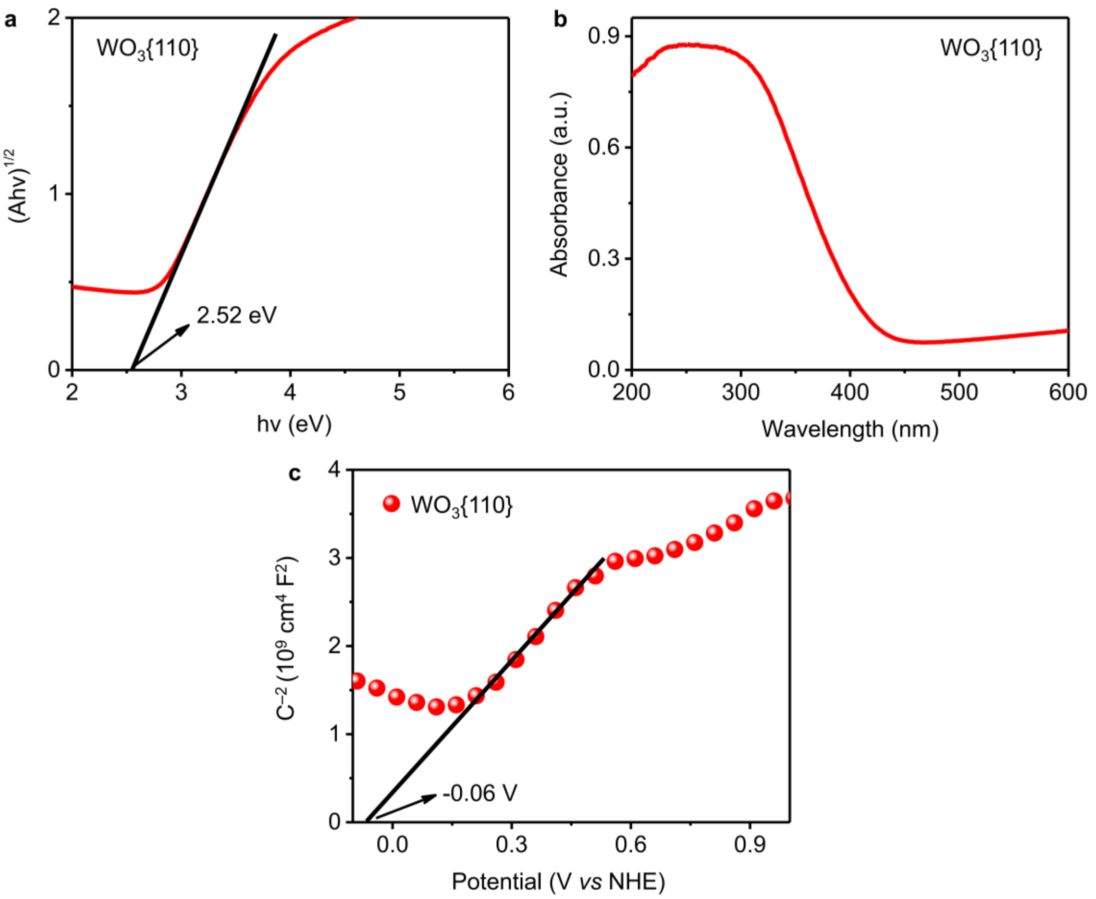

Supplementary Fig. 71 (a) Kubelka-Munk function of WO_3 {110} for bandgap test. (b) UV-visible diffuse reflectance absorption plot of WO_3 {110}. (c) Mott-Schottky test of WO_3 {110} at the frequency of 1500 Hz.

The Kubelka-Munk plot (Supplementary Fig. 71a) transformed from UV-visible diffuse reflectance spectrum (Supplementary Fig. 71b) is used to estimate the bandgap value of WO_3 {110} (2.52 eV). The conduction band of WO_3 {110} is tested to be -0.06 V *vs* NHE, which is labelled from the corresponding Mott-Schottky curve (Supplementary Fig. 71c). Based on the value of bandgap and conduction band, the valence band potential of WO_3 {110} is calculated to be 2.46 V *vs* NHE.

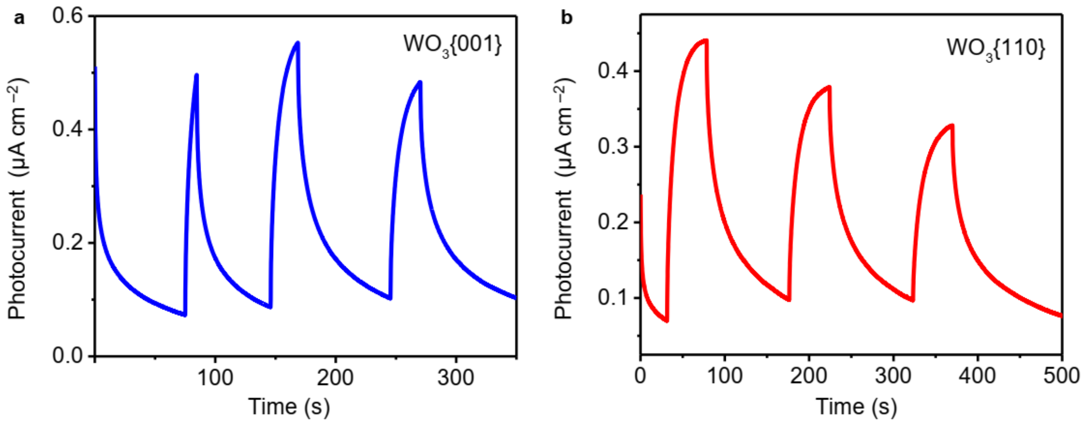

Supplementary Fig. 72 Photocurrent of (a) WO_3{001} and (b) WO_3{110}.

Based on the reports[51-52], the top of valence band of WO_3 is mainly composed of O2*p* orbitals. Once the light turns on, the valence band of WO_3 will release one electron and leaves one hole. The separation between electron and hole is reflected by the photocurrent experiment. Thus, the obvious photocurrent of WO_3{001} and WO_3{110} represents the successful activation of lattice-O.

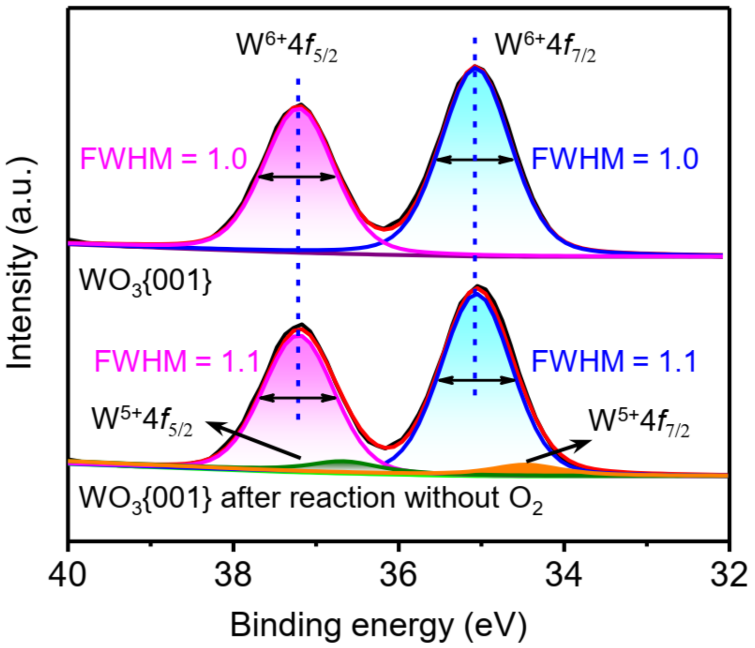

Supplementary Fig. 73 High-resolution W4*f* XPS spectra of WO_3 {001} before and after photocatalytic CH_4 oxidation without O_2 addition.

We infer that the unused electrons after reaction in pure CH_4 atmosphere are trapped in WO_3 {001} crystal, which is uncovered by the high-resolution W4*f* XPS spectra (Supplementary Fig. 73). Compared to pristine WO_3 {001} (FWHM = 1.0), the full width at half maximum (FWHM = 1.1) of W4*f* from WO_3 {001} after reaction in pure CH_4 atmosphere becomes broader. This means that the peak of W4*f* after reaction can be split. Thus, besides the $W^{6+}4f_{5/2}$ and $W^{6+}4f_{7/2}$ peaks, the peaks of $W^{5+}4f_{5/2}$ and $W^{5+}4f_{7/2}$ are separated from W4*f*, indicating that excess electrons emerge and reduce W^{6+} to W^{5+} during CH_4 oxidation without O_2.

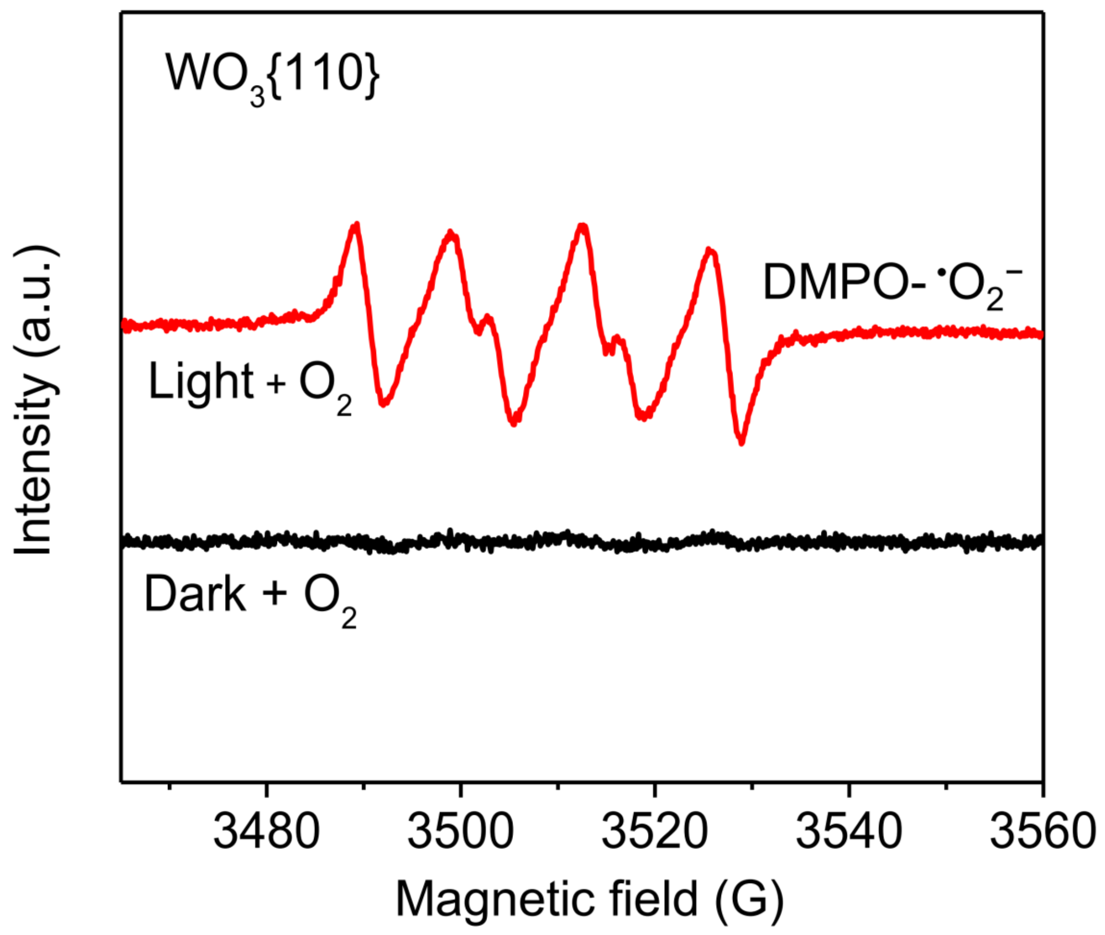

Supplementary Fig. 74 EPR signals of DMPO-$^{\bullet}O_2^-$ without CH_4 addition in WO_3\{110\} system.

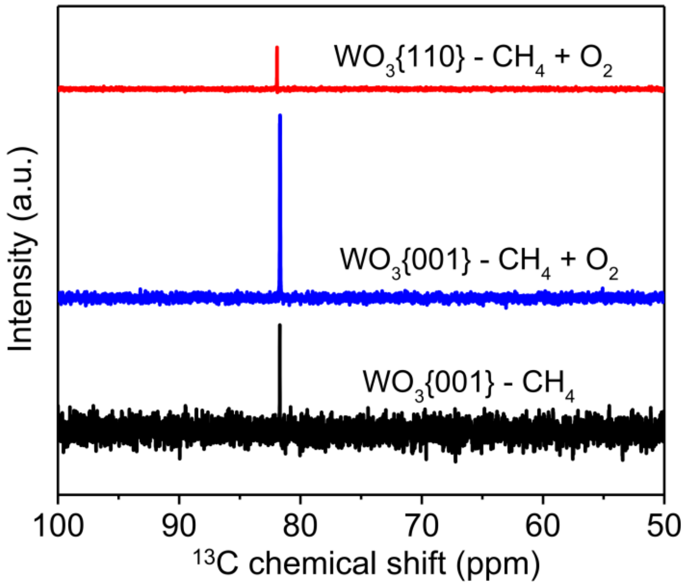

Supplementary Fig. 75 ^{13}C NMR spectra of the products generated by photocatalytic CH_4 oxidation on WO_3 {001} in CH_4 atmosphere or the mixed CH_4 and O_2 atmosphere, on WO_3 {110} in the mixed CH_4 and O_2 atmosphere at reaction temperature of 25 °C.

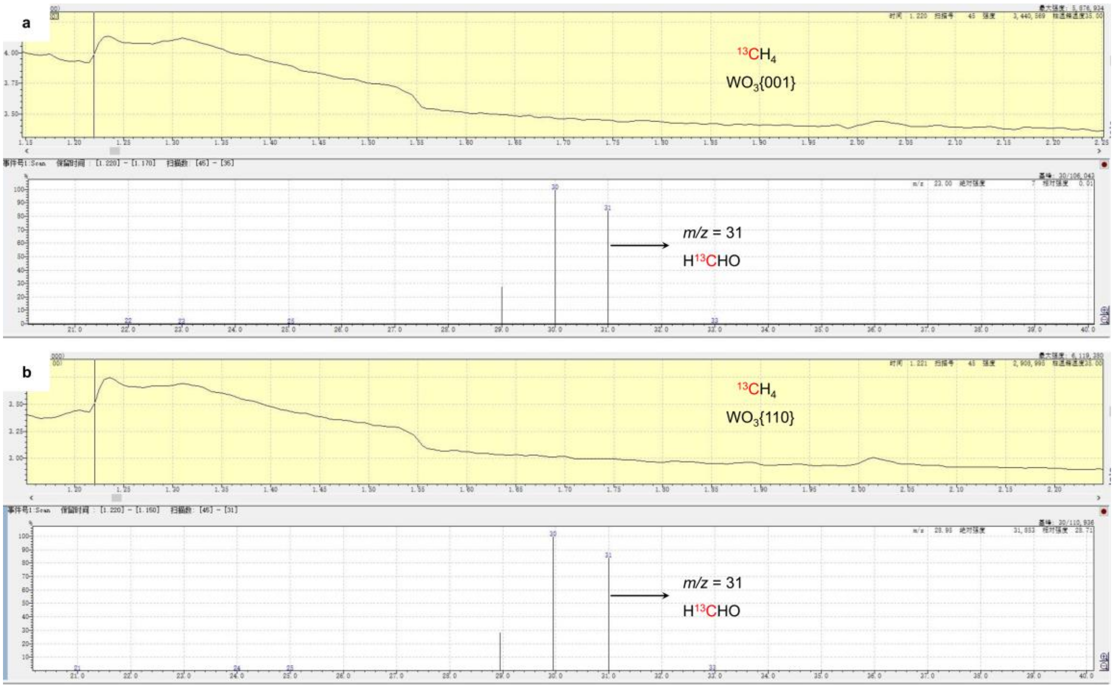

Supplementary Fig. 76 (a) Screenshot on GC-MS spectra of HCHO obtained in $WO_3\{001\}$ and (b) $WO_3\{110\}$ systems using $^{13}CH_4$ as carbon isotope.

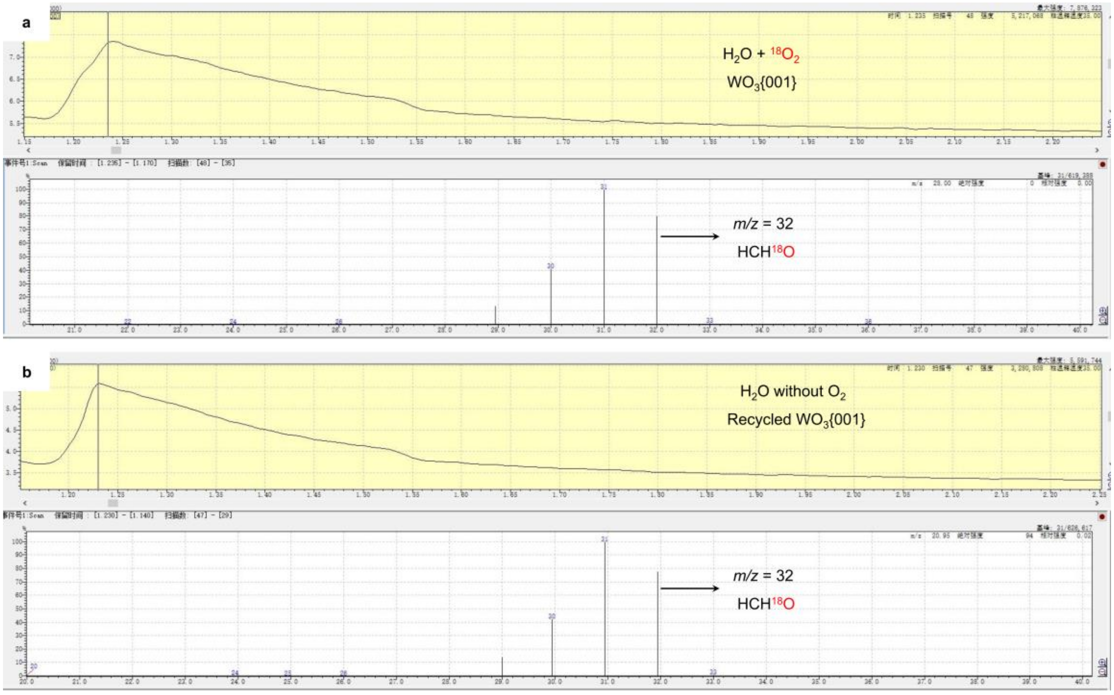

Supplementary Fig. 77 (a) Screenshot on GC-MS spectra of HCHO obtained in WO_3\{001\} system using $^{18}O_2$ as oxygen isotope and (b) the recycled WO_3\{001\} as photocatalyst without O_2 addition.

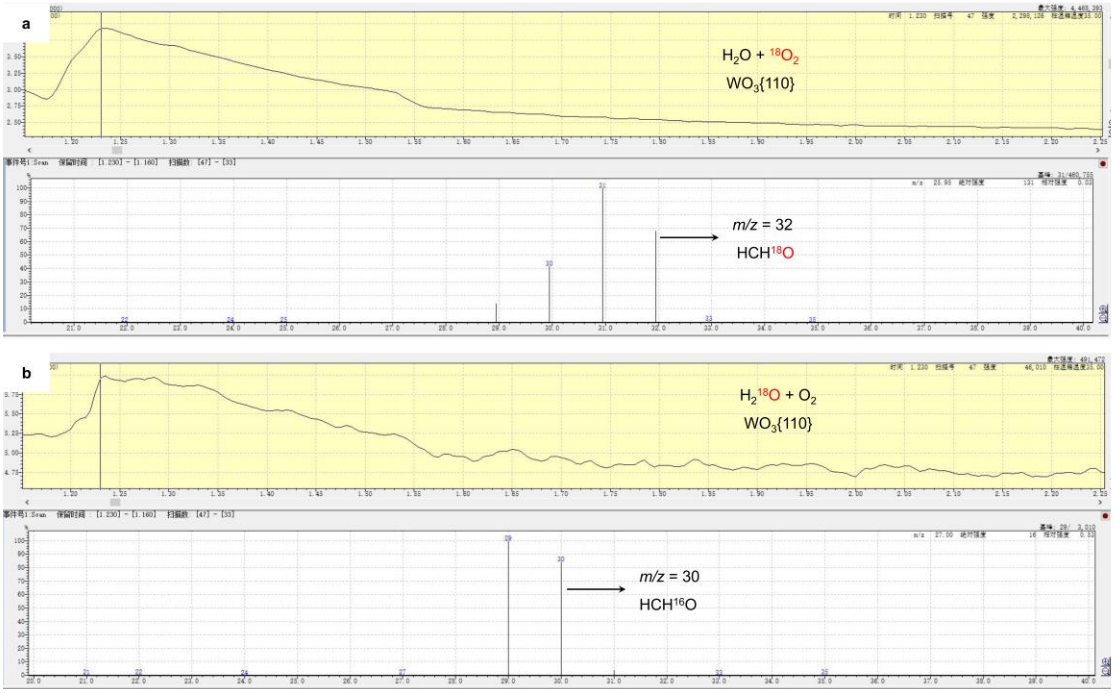

Supplementary Fig. 78 Screenshot on GC-MS spectra of HCHO obtained in $WO_3\{110\}$ system using (a) $^{18}O_2$ or (b) $H_2{}^{18}O$ as oxygen isotope.

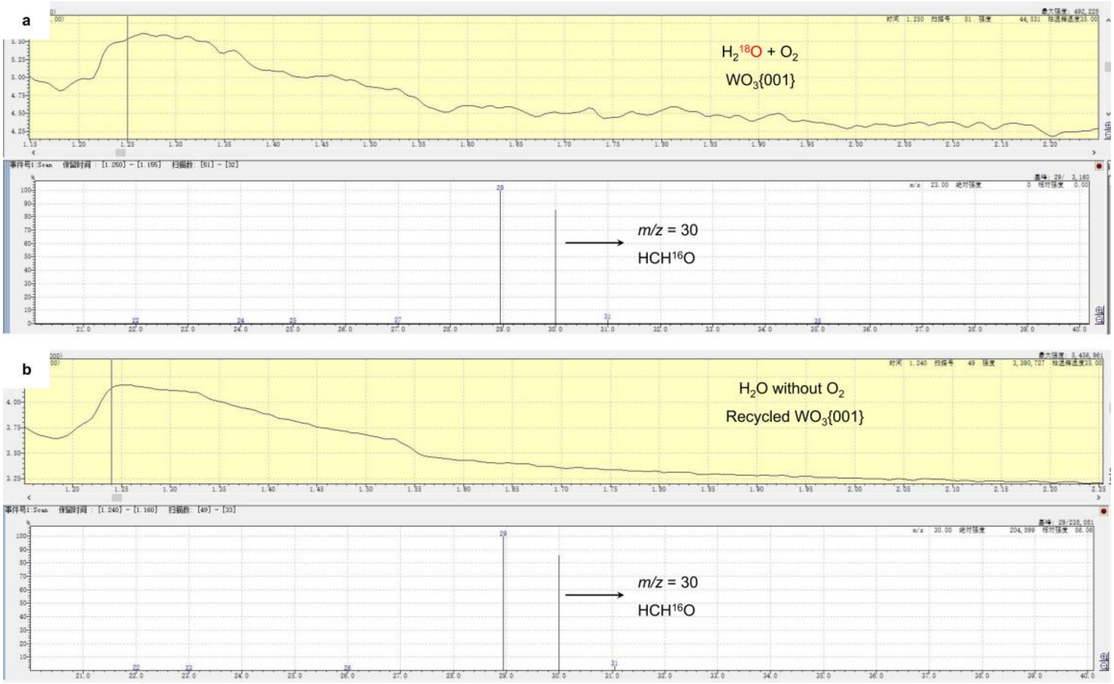

Supplementary Fig. 79 (a) Screenshot on GC-MS spectra of HCHO obtained in $WO_3\{001\}$ system using $H_2{}^{18}O$ as oxygen isotope and (b) the recycled $WO_3\{001\}$ as photocatalyst without O_2 addition.

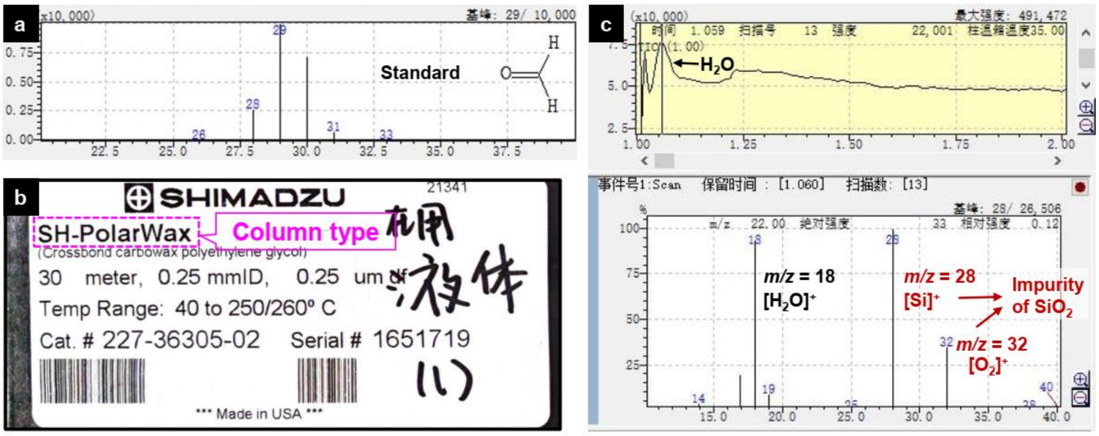

Supplementary Fig. 80 (a) Standard HCHO MS spectrum. (b) GCMS column type used in our work. (c) Two impurity peaks at *m/z* = 28 ($[Si]^+$) and 32 ($[O_2]^+$) observed in Wax column.

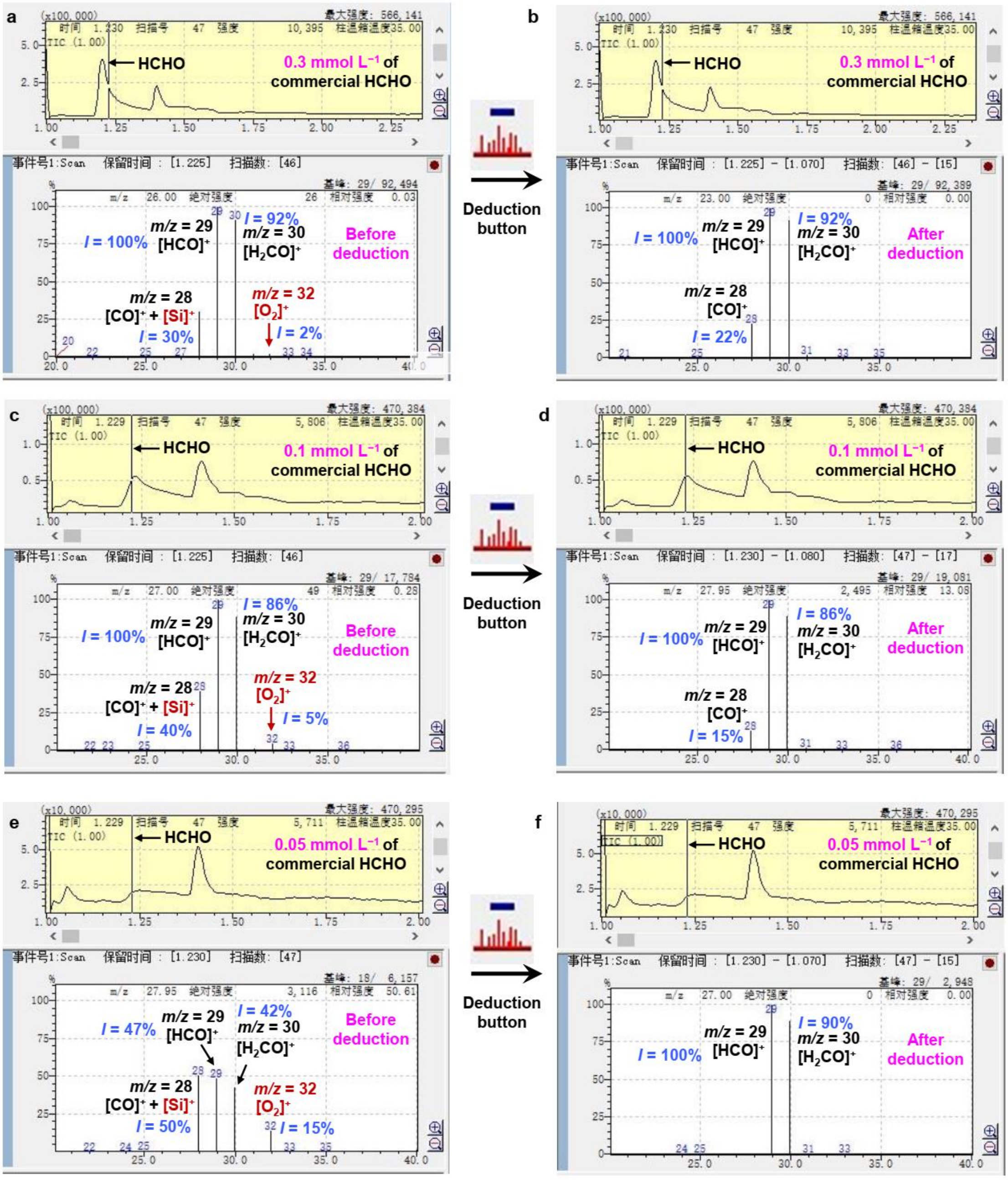

Supplementary Fig. 81 (a) GCMS spectra of commercial HCHO solution (0.3 mmol L^{-1}) before and (b) after impurity deduction. (c) GCMS spectra of commercial HCHO solution (0.1 mmol L^{-1}) before and (d) after impurity deduction. (e) GCMS spectra of commercial HCHO solution (0.05 mmol L^{-1}) before and (f) after impurity deduction.

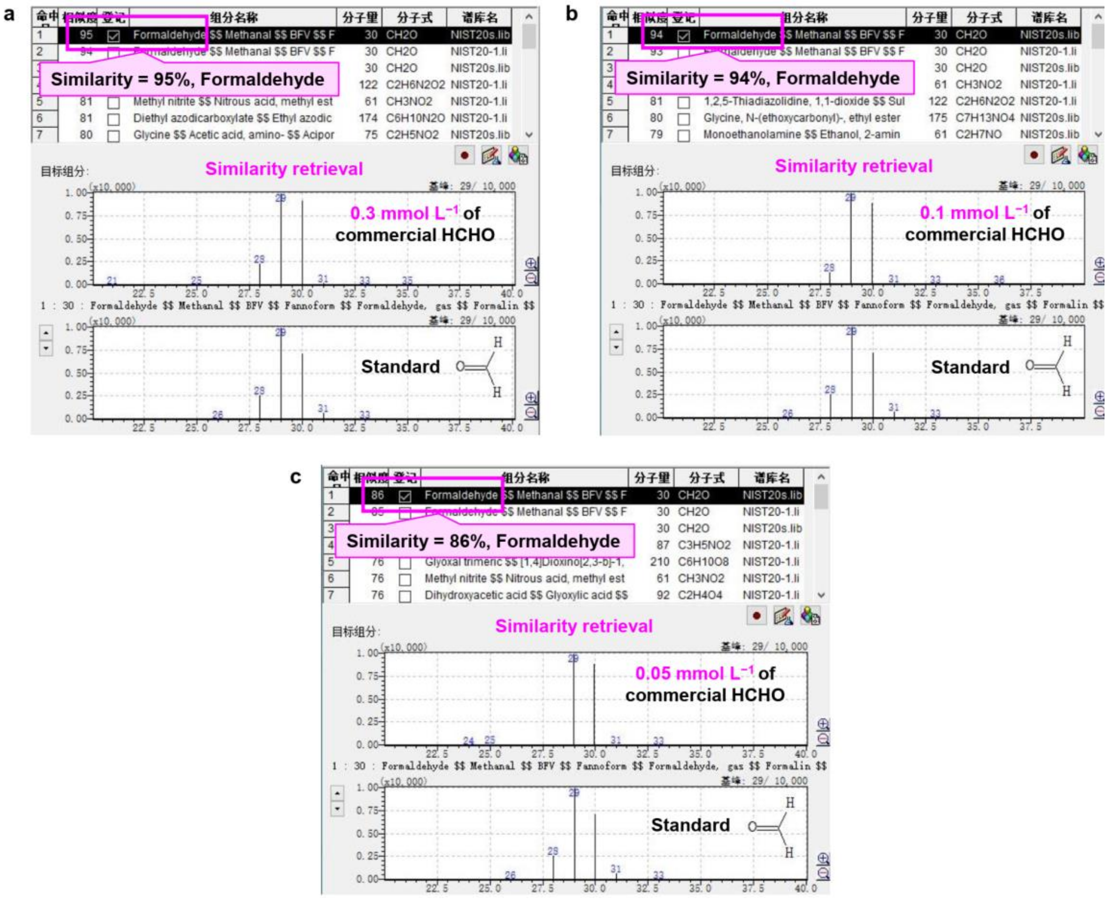

Supplementary Fig. 82 (a) Similarity retrieval for 0.3 mmol L^{-1}, (b) 0.1 mmol L^{-1}, (c) 0.05 mmol L^{-1} of commercial HCHO solution, respectively.

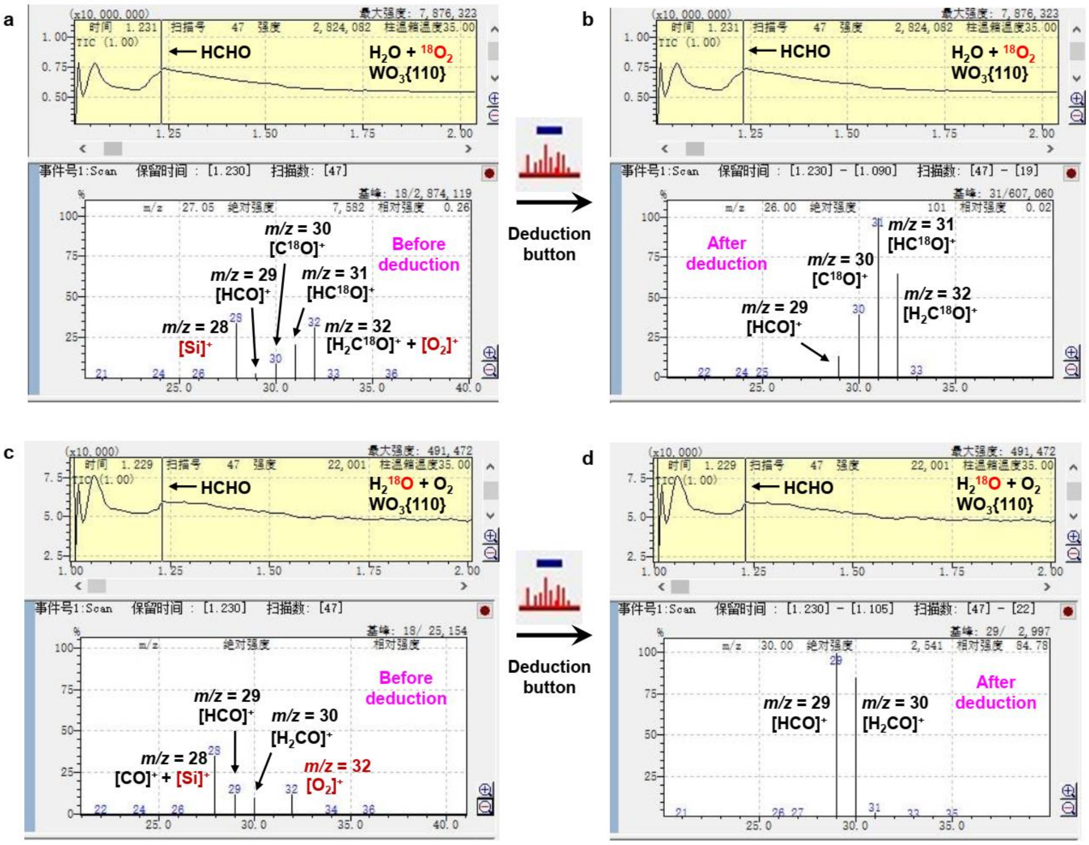

Supplementary Fig. 83 (a) GCMS spectra of HCHO obtained in $WO_3\{110\}$ system using $^{18}O_2$ as oxygen isotope before and (b) after impurity deduction. (c) GCMS spectra of HCHO obtained in $WO_3\{110\}$ system using $H_2{}^{18}O$ as oxygen isotope before and (d) after impurity deduction.

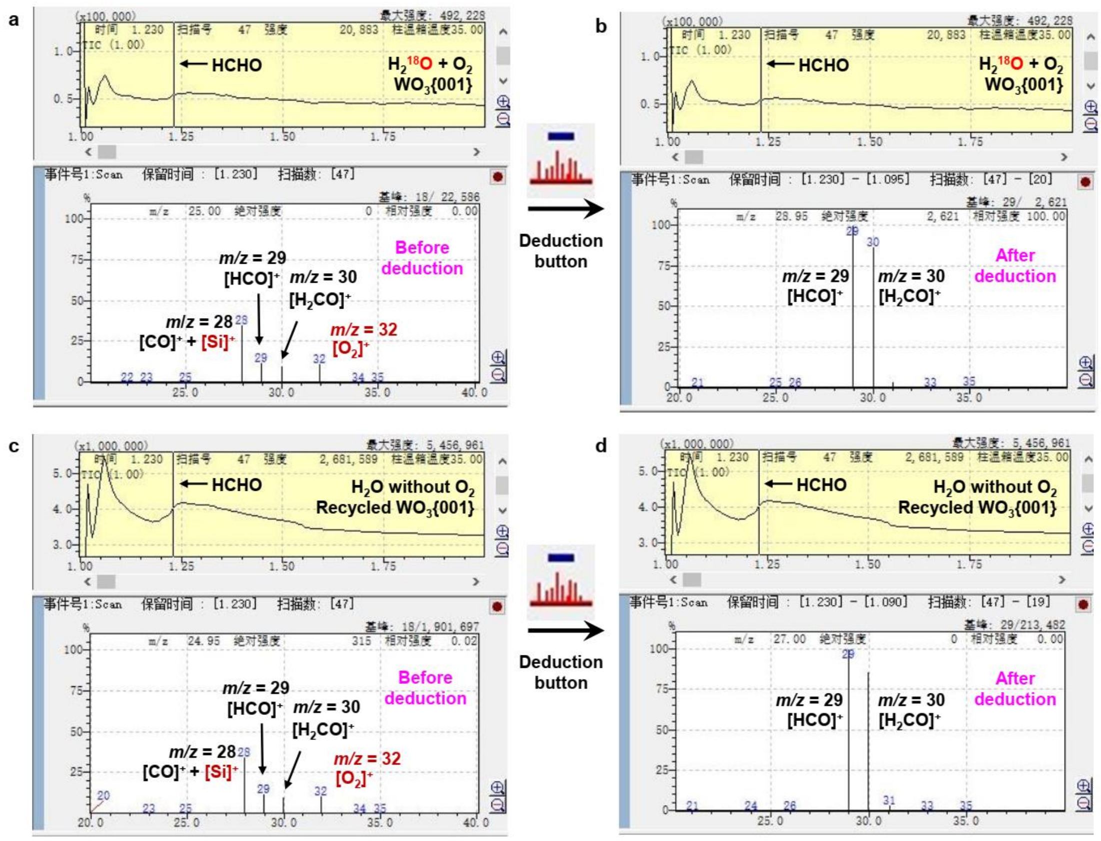

Supplementary Fig. 84 (a) GCMS spectra of HCHO obtained in WO_3 {001} system using $H_2{}^{18}O$ as oxygen isotope before and (b) after impurity deduction. (c) GCMS spectra of HCHO obtained in WO_3 {001} system using the recycled WO_3 {001} as photocatalyst without O_2 addition before and (d) after impurity deduction.

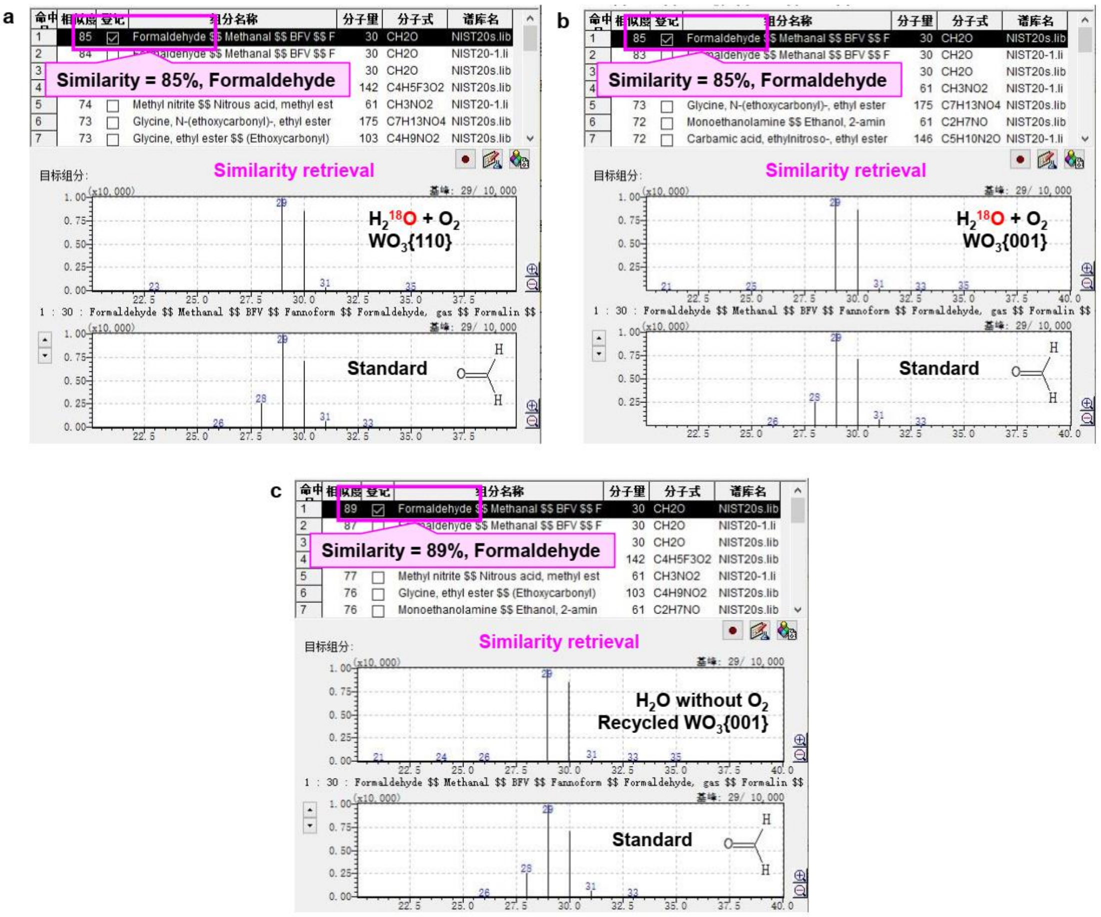

Supplementary Fig. 85 (a) Similarity retrieval of HCHO obtained in WO_3 {110} system using $H_2^{18}O$ as oxygen isotope. (b) Similarity retrieval of HCHO obtained in WO_3 {001} system using $H_2^{18}O$ as oxygen isotope. (c) Similarity retrieval of HCHO obtained in WO_3 {001} system using the recycled WO_3 {001} as photocatalyst without O_2 addition.

According to the standard GCMS spectrum of HCHO, a small peak exists at m/z = 28 (Supplementary Fig. 80a). Nevertheless, in real test, only high concentration instead of low concentration of HCHO displays the peak of m/z = 28. This is because the GCMS column for HCHO aqueous solution test is the Wax column (for example, SH-PolarWax in our work, Supplementary Fig. 80b) containing SiO_2 component, which inevitably brings two impurity peaks at m/z = 28 ($[Si]^+$) and 32 ($[O_2]^+$, Supplementary Fig. 80c). The impurity peak of $[Si]^+$ (m/z = 28) exactly overlaps that of $[CO]^+$ (m/z = 28) fragment of HCHO product. To obtain the convincing GCMS result, the impurity peak must be deducted during the GCMS spectroscopy observation, which will weaken or even erase the $[CO]^+$ peak (m/z = 28) in low concentration HCHO solution. For examples, as shown in Supplementary Fig. 81a and 81c, for high HCHO concentrations (0.3 mmol

L^{-1} and 0.1 mmol L^{-1}) before SiO_2 impurity peaks deduction, the GCMS spectra are almost identical to the standard spectra of HCHO (Supplementary Fig. 80a). However, through careful observation, one can find that compared with 0.3 mmol L^{-1} HCHO solution ($[H_2CO]^+$, I = 92%; $[CO]^+$ + $[Si]^+$, I = 30%; $[O_2]^+$, I = 2%), the peak of 0.1 mmol L^{-1} HCHO solution at m/z = 30 ($[H_2CO]^+$, I = 86%) is lower while the peak at m/z = 28 ($[CO]^+$ + $[Si]^+$, I = 40%) and the peak at m/z = 32 ($[O_2]^+$, I = 5%) become more obvious. Therein, the I is the percentage of peak intensity. This is because the lower concentration of 0.1 mmol L^{-1} HCHO solution leads to a decrease in the proportion of HCHO ion fragments and an increase in the proportion of impurity ion fragments. After impurity deduction, the peak intensity of m/z = 28 ($[Si]^+$) and m/z = 32 ($[O_2]^+$) (Fig. 81b and Fig. 81d) decreases and disappears, respectively. The corrected HCHO GCMS spectrum shows 95% (Supplementary Fig. 82a) and 94% (Supplementary Fig. 82b) similarity with the standard HCHO spectrum. While, for the HCHO solution of low concentration (0.05 mmol L^{-1}, Supplementary Fig. 81e), its ion fragment abundance would be lower than the impurity abundance ($[HCO]^+$, I = 47%; $[H_2CO]^+$, I = 30%; $[CO]^+$ + $[Si]^+$, I = 50%; $[O_2]^+$, I = 15%). Through impurity deduction, the impurity ion fragments of $[Si]^+$ and $[O_2]^+$ are removed, and the I value of $[HCO]^+$ and $[H_2CO]^+$ is enhanced to 100% and 90%, respectively. We also note that because the concentration of HCHO is lower than that of the impurity abundance and its $[CO]^+$ has the same m/z value with $[Si]^+$, no peak of $[CO]^+$ at m/z = 28 is observed for 0.05 mmol L^{-1} HCHO after the impurity deduction (Supplementary Fig. 81f). The absence of m/z = 28 does not negate the qualitative accuracy of HCHO, which is proved by its 86% similarity with the standard HCHO spectrum (Supplementary Fig. 82c). Similarly, for Fig. 9c and 9d in our work, the missing of m/z =28 in low HCHO concentration test is reasonable after impurity deduction (Supplementary Fig. 83a–83d and Supplementary Fig. 84a–84d) with the high similarity (Supplementary Fig. 85a–85c).

Supplementary Tables

Supplementary Table 1 Q.E. values of WO_3 {001} and WO_3 {110} irradiated by different monochromatic light of 365, 420, 470, 535 and 630 nm, respectively.

Catalyst	Wavelength (nm)	Light intensity (mW cm^{-2})	E_λ (J)	Number of products (μmol)		Q.E. (%)
				HCHO	CO_2	
WO_3 {001}	365	6.26	5.4×10^{-19}	38.70	2.00	6.54
	420	22.50	4.7×10^{-19}	13.47	0.36	0.53
	470	24.56	4.2×10^{-19}	0	0	0
	535	13.81	3.7×10^{-19}	0	0	0
	630	13.82	3.2×10^{-19}	0	0	0
WO_3 {110}	365	6.26	5.4×10^{-19}	32.16	0.11	4.96
	420	22.50	4.7×10^{-19}	11.084	0	0.41
	470	24.56	4.2×10^{-19}	0	0	0
	535	13.81	3.7×10^{-19}	0	0	0
	630	13.82	3.2×10^{-19}	0	0	0

Supplementary Table 2 Contrast experiment results.

Entry	Light	Catalyst	CH_4 (bar)	O_2 (bar)	H_2O (mL)	Products (μmol m^{-2})
1	--	WO_3\{001\}	13	7	5	0
2	--	WO_3\{110\}	11	9	5	0
3	Yes	--	13	7	5	0
4	Yes	--	11	9	5	0
5	Yes	WO_3\{001\}	--	20	5	0
6	Yes	WO_3\{110\}	--	20	5	0

Reaction condition: catalyst mass = 10 mg; light irradiation by Xenon lamp with wavelength of 300 nm < λ < 700 nm and light intensity of 150 mW cm^{-2}; cooling water = 25 ºC.

Supplementary Table 3 XPS peak intensities of O1*s* before and after reactions in CH_4 atmosphere on WO_3\{001\} and WO_3\{110\}.

Condition	Types of O1*s*	WO_3\{001\} (intensity × 10^4)	WO_3\{110\} (intensity × 10^4)
Before reaction	Lattice-O	5.39	6.26
	OH	1.41	2.04
	Adsorbed O_2	0	0
	C=O	0	0
After reaction	Lattice-O	4.03	4.06
	OH	1.41	1.49
	Adsorbed O_2	1.45	1.60
	C=O	1.29	0

Supplementary Table 4 XPS peak intensities of O1*s* before and after reactions in CH_4 + O_2 atmosphere on WO_3{001} and WO_3{110}.

Condition	Types of O1s	WO_3{001} (intensity × 10^4)	WO_3{110} (intensity × 10^4)
Before reaction	Lattice-O	5.39	6.26
	OH	1.41	2.04
	Adsorbed O_2	0	0
	C=O	0	0
After reaction	Lattice-O	5.33	5.39
	OH	1.41	1.72
	Adsorbed O_2	1.32	1.72
	C=O	1.33	1.59

The quantitative O1*s* XPS data of both WO_3{001} and WO_3{110} before and after photocatalytic reaction in a mixed CH_4 + O_2 atmosphere has been provided as shown in Supplementary Table S4. Before reaction, the peak intensity of lattice-O in the O1*s* XPS spectra of WO_3{110} is 6.26 × 10^4. However, after the reaction in CH_4 + O_2 atmosphere, the lattice-O intensity becomes 5.39 × 10^4. This indicates that even in the O_2 atmosphere, 13.9% of the lost lattice-O in the WO_3{110} system has not been repaired. On the contrary, nearly 100% of lost lattice-O in the WO_3{001} system has been fully restored.

Supplementary References

1. Lin, R. et al. Quantitative Study of Charge Carrier Dynamics in Well-Defined WO_3 Nanowires and Nanosheets: Insight into the Crystal Facet Effect in Photocatalysis. *J. Am. Chem. Soc.* **140**, 9078-9082 (2018).
2. Safo, I. et al. The role of Polyvinylpyrrolidone (PVP) as Capping and Structure-Directing Agent in the Formation of Pt Nanocubes. *Nanoscale Adv.* **1**, 3095-3106 (2019).
3. Wang, X. et al. Surface stabilization of hexagonal WO_3 by non-metallic atoms: A DFT study. *Comp. Mater. Sci.* **68**, 218-221 (2013).
4. Zhu, J. et al. Hexagonal single crystal growth of WO_3 nanorods along a [110] axis with enhanced adsorption capacity. *Chem. Commun.* **47**, 4403–4405 (2011).
5. Kresse, Furthmuller, Efficient iterative schemes for ab initio total-energy calculations using a plane-wave basis set. *Phys. Rev. B, Condens Matter* **54**, 11169-11186 (1996).
6. Steneteg, P. et al. Wave function extended Lagrangian Born-Oppenheimer molecular dynamics. *Phys. Rev. B* **82**, 075110 (2010).
7. Perdew, J. P., Burke, K., Ernzerhof, M., Generalized Gradient Approximation Made Simple. *Phys. Rev. Lett.* **77**, 3865-3868 (1996).
8. Blöchl, P. E., Projector augmented-wave method. *Phys. Rev. B* **50**, 17953-17979 (1994).
9. Kresse, G., Joubert, D., From ultrasoft pseudopotentials to the projector augmented-wave method. *Phys. Rev. B* **59**, 1758-1775 (1999).
10. Grimme, S. et al. A consistent and accurate ab initio parametrization of density functional dispersion correction (DFT-D) for the 94 elements H-Pu. *J. Chem. Phys.***132**, 154104 (2010).
11. Ji, Y. F., Luo, Y., New Mechanism for Photocatalytic Reduction of CO_2 on the Anatase TiO_2(101) Surface: The Essential Role of Oxygen Vacancy. *J. Am. Chem. Soc.* **138**, 15896-15902 (2016).
12. Nolan, M., Hybrid density functional theory description of oxygen vacancies in the CeO_2(110) and (100) surfaces. *Chem. Phys. Lett.* **499**, 126-130 (2010).
13. Castleton, C. W. M., Kullgren, J., Hermansson, K., Tuning LDA+U for electron localization and structure at oxygen vacancies in ceria. *J. Chem. Phys.* **127**, 244704

(2007).

14. Fabris, S. et al. Electronic and Atomistic Structures of Clean and Reduced Ceria Surfaces. *J. Chem. Phys. B* **109**, 22860-22867 (2005).
15. He, Y. et al. In Situ Identification of Reaction Intermediates and Mechanistic Understandings of Methane Oxidation over Hematite: A Combined Experimental and Theoretical Study. *J. Am. Chem. Soc.* **142**, 17119-17130 (2020).
16. Luo, L. et al. Synergy of Pd atoms and oxygen vacancies on In_2O_3 for methane conversion under visible light. *Nat. Commun.* **13**, 2930 (2022).
17. Nolan, M., Fearon, J. E. & Watson, G. W. Oxygen vacancy formation and migration in ceria. *Solid State Ionics* **177**, 3069–3074 (2006).
18. Mori-Sánchez, P., Cohen, A. J., Yang, W., Localization and Delocalization Errors in Density Functional Theory and Implications for Band-Gap Prediction. *Phys. Rev. Lett.* **100**, 146401 (2008).
19. Liang, Z., Li, T., Kim, M., Asthagiri, A. & Weaver, J. Low-temperature activation of methane on the IrO_2(110) surface. *Science* **356**, 298−301 (2017).
20. Zhang, W. et al. High-performance photocatalytic nonoxidative conversion of methane to ethane and hydrogen by heteroatoms-engineered TiO_2. *Nat. Commun.* **13**, 2806 (2022).
21. Lampert, J., Kazi, M. & Farrauto, R. Palladium catalyst performance for methane emissions abatement from lean burn natural gas vehicles. *Appl. Catal. B: Environ.* **14**, 211-223 (1997).
22. Myshakin, E., Jiang, H., Warzinski, R. & Jordan, K. Molecular Dynamics Simulations of Methane Hydrate Decomposition. *J. Phys. Chem. A* **113**, 1913−1921 (2009).
23. Dicks, A., Pointon, K. & Siddle, A. Intrinsic reaction kinetics of methane steam reforming on a nickel/zirconia anode. *J. Power Sources* **86**, 523−530 (2000).
24. Boukhalfa, N. Chemical Kinetic Modeling of Methane Combustion. *Procedia Eng.* **148**, 1130−1136 (2016).
25. Bonard, A., Daële, V., Delfau, J. & Vovelle, C. Kinetics of OH Radical Reactions with Methane in the Temperature Range 295−660 K and with Dimethyl Ether and Methyl-tert-butyl Ether in the Temperature Range 295−618 K. *J. Phys. Chem. A* **106**, 4384−4389 (2002).
26. Zavitsas, A. Energy Barriers to Chemical Reactions. Why, How, and How Much?

Non-Arrhenius Behavior in Hydrogen Abstractions by Radicals. *J. Am. Chem. Soc.* **120**, 6578−6586 (1998).

27. Pilgrim, J., McIlroy, A. & Taatjes, C. Kinetics of Cl Atom Reactions with Methane, Ethane, and Propane from 292 to 800 K. *J. Phys. Chem. A* **101**, 1873−1880 (1997).
28. Chen, C., Back, M. & Back, R. The thermal decomposition of methane. II. Secondary reactions, autocatalysis and carbon formation; non-Arrhenius behaviour in the reaction of CH_3 with ethane. *Can. J. Chem.* **54**, 3175−3184 (1976).
29. Luo, L. et al. Nearly 100% selective and visible-light-driven methane conversion to formaldehyde via. single-atom Cu and $W^{\delta+}$. *Nat. Commun.* **14**, 2690 (2023).
30. Zhou, W. et al. Highly selective aerobic oxidation of methane to methanol over gold decorated zinc oxide via photocatalysis. *J. Mater. Chem. A*, **8**, 13277-13284 (2020).
31. Fan Y., et al. Selective photocatalytic oxidation of methane by quantum-sized bismuth vanadate. *Nat. Sustain.* **4**, 509-515 (2021).
32. Pastor, E. et al. Electronic defects in metal oxide photocatalysts. *Nat. Rev. Mater.* **7**, 503–521 (2022).
33. Wang, Z.-T., Deskins, N. A., Lyubinetsky, I., Direct Imaging of Site-Specific Photocatalytical Reactions of O_2 on TiO_2(110). *J. Phys. Chem. Lett.* **3**, 102-106. (2012)
34. Anglada, J. M. et al. The atmospheric oxidation of CH_3OOH by the OH radical: the effect of water vapor. *Phys. Chem. Chem. Phys.* **19**, 12331-12342 (2017).
35. Tan, S. J. et al. Observation of Photocatalytic Dissociation of Water on Terminal Ti Sites of TiO_2(110)-1 x 1 Surface. *J. Am. Chem. Soc.* **134**, 9978−9985 (2012).
36. Yan, J. Q. et al. Single atom tungsten doped ultrathin α-$Ni(OH)_2$ for enhanced electrocatalytic water oxidation. *Nat. Commun.* **10**, 2149 (2019).
37. Li, H. et al. Oxygen Vacancy Associated Surface Fenton Chemistry: Surface Structure Dependent Hydroxyl Radicals Generation and Substrate Dependent Reactivity. *Environ. Sci. Technol.* **51**, 5685−5694 (2017).
38. Jiang, Y. et al. Enabling Specific Photocatalytic Methane Oxidation by Controlling Free Radical Type. *J. Am. Chem. Soc.* **145**, 2698−2707 (2023).
39. Luo, L. et al. Water enables mild oxidation of methane to methanol on gold single-atom catalysts. *Nat. Commun.* **12**, 1218 (2021).
40. Song, H. et al. Direct and Selective Photocatalytic Oxidation of CH_4 to Oxygenates with O_2 on Cocatalysts/ZnO at Room Temperature in Water. *J. Am. Chem. Soc.* **141**,

20507-20515 (2019).

41. Agarwal, N. et al. Aqueous Au-Pd colloids catalyze selective CH_4 oxidation to CH_3OH with O_2 under mild conditions. *Science* **358**, 223–227 (2017).
42. Nosaka, Y.; Nosaka, A. Y., Generation and Detection of Reactive Oxygen Species in Photocatalysis. *Chem. Rev.* **117**, 11302−11336 (2017).
43. Reed, J. J., Digitizing "The NBS Tables of Chemical Thermodynamic Properties: Selected Values for Inorganic and C1 and C2 Organic Substances in SI Units". *J. Res. Natl. Inst. Stand. Technol.* **125**, 125007 (2020).
44. Arnano, F. et al. Photoelectrochemical Homocoupling of Methane under Blue Light Irradiation. *ACS Energy Lett.* **4**, 502−507 (2019).
45. Iwamoto, M. et al. Study of metal oxide catalysts by temperature programmed desorption. 4. Oxygen adsorption on various metal oxides. *J. Phys. Chem.* **82**, 2564-2570 (1978).
46. Song, S. et al. A selective Au-ZnO/TiO_2 hybrid photocatalyst for oxidative coupling of methane to ethane with dioxygen. *Nat. Catal.* **5**,78 (2022)
47. Greczynski, G., Hultman, L., Compromising Science by Ignorant Instrument Calibration-Need to Revisit Half a Century of Published XPS Data. *Angew. Chem. Int. Ed.* **59**, 5002-5006 (2020).
48. Zhang, J. et al. Hydrothermally synthesized WO_3 nanowire arrays with highly improved electrochromic performance. *J. Mater. Chem.* **21**, 5492-5498 (2011).
49. Bard, A. J., Parsons, R., Jordan, J., Standard Potentials in Aqueous Solution. Standard Potentials in Aqueous Solution: *New York* (1985).
50. Wu, R. et al. CH4 activation and sensing on hexagonal WO_3 (001) and (110) surfaces. *Appl. Surf. Sci.* **481**, 1154-1159 (2019).
51. Zhang, N. et al. Monoclinic Tungsten Oxide with {100} Facet Orientation and Tuned Electronic Band Structure for Enhanced Photocatalytic Oxidations. *ACS Appl. Mater. Interfaces* **8**, 10367−10374 (2016).
52. Albanese, E., Di Valentin, C. & Pacchioni, G. H_2O Adsorption on WO_3 and WO_{3-x} (001) Surfaces. *ACS Appl. Mater. Interfaces* **9**, 23212−23221 (2017).
